# Heavy is the Crown: Crown Ether Modulation of Cobalt Porphyrin CO_2_ Electroreduction in Zero‐Gap Electrolyzers

**DOI:** 10.1002/anie.202525189

**Published:** 2026-02-02

**Authors:** Wiebke Wiesner, Christian Wilhelm, Rahel Cornelia Hoffmann, Peter Stahl, Kevinjeorjios Pellumbi, Julia Jökel, Ivana Ivanović‐Burmazović, Ulf‐Peter Apfel

**Affiliations:** ^1^ Lehrstuhl Für Anorganische Chemie I Ruhr‐Universität Bochum Bochum Germany; ^2^ Department Chemie Ludwig‐Maximilians‐Universität München München Germany; ^3^ Department Power‐to‐Chemicals Fraunhofer‐Institut Für Umwelt‐ Sicherheits‐ und Energietechnik UMSICHT Oberhausen Germany

**Keywords:** cobalt complexes, co_2_ reduction, electrochemistry, porphyrinoids, zero‐gap cell

## Abstract

Since decades, metalloporphyrins have been studied to catalyze the electrochemical CO_2_ reduction (eCO_2_R) with the most recent studies focusing on immobilized complexes aiming for heterogeneous, scalable catalysis. However, reports for the application in industrially relevant zero‐gap type electrolyzer cells (ZGEs) are especially rare. Herein we present the synthesis of four novel crown ether (CE) substituted cobalt porphyrins to benefit from an increased local cation concentration. Following their electrochemical characterization all catalysts have been tested in ZGEs. Experiments under laboratory‐scale conditions (≤100 mA/cm^2^) revealed that the positioning of the CE influences the catalytic performance in terms of Faradaic Efficiency for CO (FE_CO_) as well as cell voltage. A maximum selectivity for CO of 96% at 100 mA/cm^2^ is reached, ranking the *ortho* substituted complex among the best state of the art systems. Post‐mortem analysis of the prepared electrodes proved that the introduction of CEs enhances the complex stability significantly. At higher current densities (≤500 mA/cm^2^) the positioning of the CEs is less impactful. Instead, the type and concentration of cations in the reactor play a dominant role determining reaction performance, achieving up to 43% FE_CO_ at 300 mA/cm^2^ with a high potassium concentration.

## Graphical abstract

The implementation of crown ethers into the ligand of cobalt porphyrins benefits the electrochemical CO_2_ reduction in the several ways: (1) the reduction potential is lowered in presence of K^+^; (2) it boosts the CO generation in Zero‐Gap Electrolyzers and (3) ensures complex stability during electrolysis.

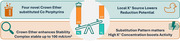

## 1 Introduction

**FIGURE 1:**
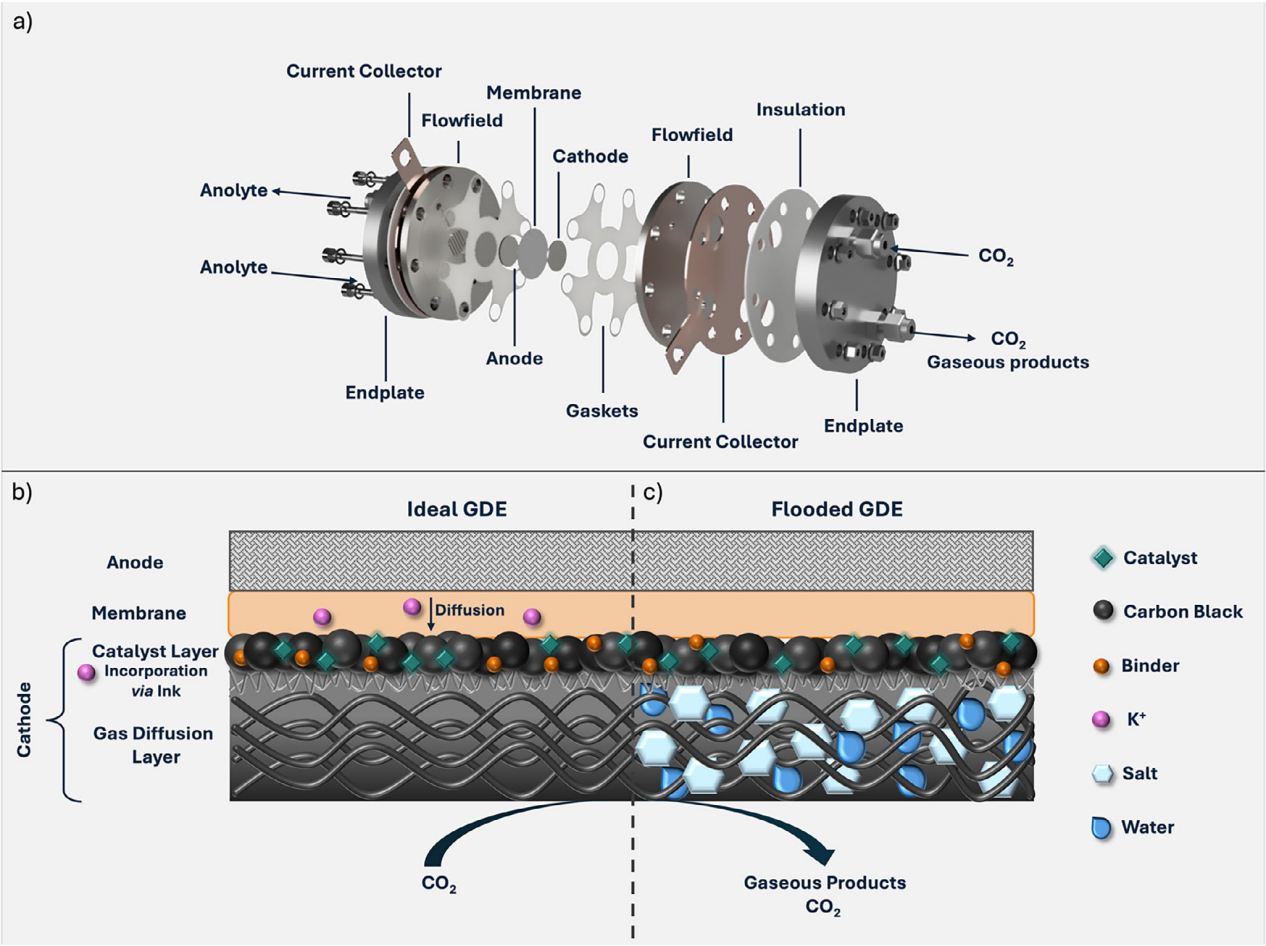
(a) Schematic representation of a ZGE highlighting all important components: endplates, current collectors, flow fields, cathode, anode, and membrane. This figure was modified from ref. [9] under the creative common license CC BY 4.0; Schematic representation of the membrane electrode assembly consisting of the GDE (comprising gas diffusion and catalyst layer), the solid‐polymer membrane and anode (b) in operational mode, and (c) flooded.

Metalloporphyrins have been studied as catalysts for electrochemical CO_2_ reduction (eCO_2_R) for several decades leading to numerous reports with a broad variety of tested metal centers and ligand systems. Though most reports focus on laboratory scale testing in solution and batch type reactor cells [1, 2, 3, 4, 5]. To drive research into a more application orientated direction scalable flow reactors, such as zero‐gap electrolyzer cells (ZGEs; Figure 1a) are needed. A ZGE comprises a gas diffusion electrode (GDE) a porous, layered carbon material, which ensures a direct delivery of gaseous CO_2_ to the catalyst (Figure 1b), and is in direct contact with the anode *via* a solid‐polymer membrane. This assembly offers several advantages for energy efficient eCO_2_R compared to other reactor types like H‐Type or Flow Cells [6, 7, 8, 9]. Due to the absence of liquid catholyte or anolyte compartments, no energy losses caused by the electrolyte resistances occur. Furthermore, the absence of a liquid catholyte increases the mass transport of CO_2_, which is commonly limited by its solubility in the electrolytes.

To combine the features of a ZGE with the unique tunability of molecular catalysts, the heterogenization of transition metal complexes onto carbon substrates emerged during recent years [7, 10, 11, 12, 13]. Therefore, complexes are commonly immobilized onto a GDE, alongside a conductive carbon support and an ionomeric binder (Figure 1b). The interplay of all components regulates key properties such as porosity, hydrophobicity and water transport within the catalyst layer, to enhance the eCO_2_R activity against the competing hydrogen evolution reaction (HER) [7, 12, 14, 15, 16].

Controlling this interplay becomes even more important when considering the degradation mechanisms that GDEs face during electrolysis. Notably, higher cation concentrations increase the chance of GDE flooding, a phenomenon in which the pores of a GDE are blocked by water molecules or formed salt crystals, consecutively interrupting the delivery of CO_2_ to the catalyst (Figure 1c) [17, 18, 19].

Metalloporphyrins, especially the iron‐based complexes, have been thoroughly investigated in the past for eCO_2_R: both homogenous and heterogenized. However, the catalytic activity of the iron tetraphenylporphyrin complex, when applied in a ZGE with comparably harsh conditions, does not come close to its efficiency in solution or immobilized in H‐Type cells [2, 7, 13, 20, 21, 22]. Investigations on potential ligand influences, as done in solution, are yet missing. In contrast to its negligible activity in homogenous eCO_2_R, the cobalt based tetraphenylporphyrin CoTPP was found to be highly active when immobilized and the best non‐noble metal based porphyrin tested in a ZGE to date, reaching a FE_CO_ of 75% at 50 mA/cm^2^ and 38% at 100 mA/cm^2^ [13, 23, 24]. Furthermore, X‐ray photoelectron spectroscopy (XPS) proved that the initial complex stayed intact during catalysis and does not degrade into metal particles or oxides as often observed for molecular complexes [13, 16, 25]. Hence, the structure of the CoTPP is an excellent basis for further tuning of the catalyst toward reaching higher FE_CO_, especially at more elevated current densities.

**FIGURE 2:**
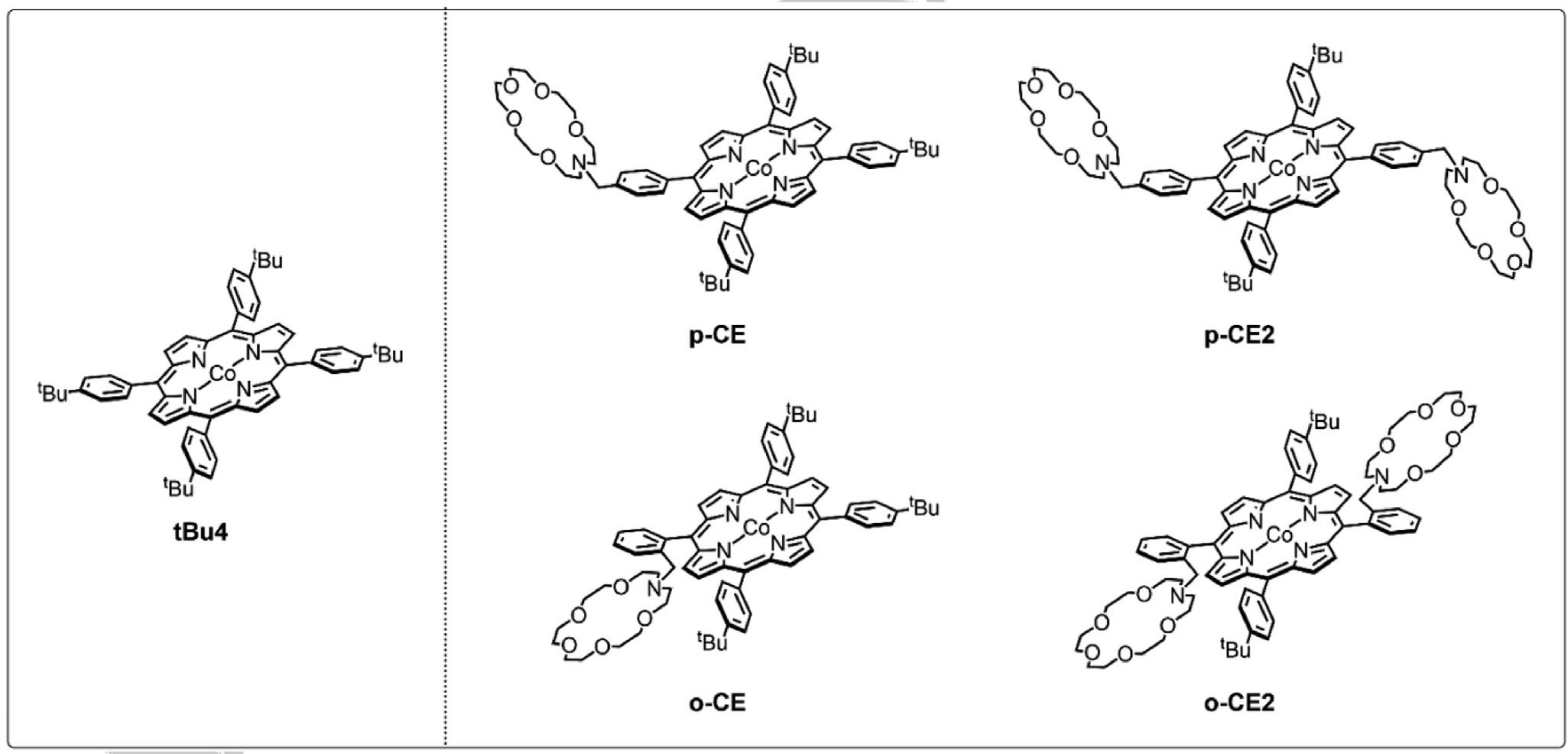
Chemical structures of the cobalt complexes **tBu4**, **p‐CE**, **p‐CE2**, **o‐CE**, and **o‐CE2**.

In H‐type cells employing immobilized porphyrin complexes, the introduction of electron‐donating substituents has shown to enhance eCO_2_R activity [24, 26]. Accordingly, *tert*‐butyl groups were introduced at the phenyl rings in the present study (tBu4, Figure 2). Besides electron donating groups, moieties that can host cations, such as crown ethers (CE), have also been shown to improve eCO_2_R activity of transition metal complexes in solution. The CE moieties host alkali cations that can stabilize reaction intermediates, as well as block the coordination sphere from water molecules to suppress HER [27, 28, 29, 30, 31]. In heterogenous eCO_2_R the presence of heavy alkali cations resulted in the same effects: CO_2_ activation *via* intermediate stabilization and promoted H_2_O dissociation from the electrode surface [32, 33, 34]. Furthermore, for GDEs it has been observed that the addition of alkali salts to the catalyst layer increased the eCO_2_R activity [35]. Here, ZGEs offer a unique control environment as alkali‐ions can cross to the catalytic layer through the membrane, but can also be directly impregnated into the catalytic layer prior to electrolysis (Figure 1b), offering two different control points compared to flow cells, in which only a bulk electrolyte is used.

Therefore, we set out to understand how molecular catalysts able to host alkali ions can allow for a more tailored control of the local cation concentration inside of the catalyst layer. Herein, we present four novel CoTPP based catalysts (Figure 2), which comprise electron donating *tert*‐butyl groups and either no, one, or two 1‐aza‐18‐crown‐6 moieties in the *para‐* or *ortho*‐position of the phenyl rings, in order to boost the catalytic eCO_2_R performance of CoTPP based systems in ZGEs, under both laboratory‐scale screening conditions (r.t., current densities ≤ 100 mA/cm^2^) and industrially‐focused ones (60 °C, current densities ≥ 300 mA/cm^2^). Our investigation provides insights into how molecular catalyst design and process control affect catalytic performance, laying focus on post‐mortem analysis to holistically evaluated molecular systems in ZGEs.

## 2 Results and Discussion

### 2.1 Synthesis

**FIGURE 3:**
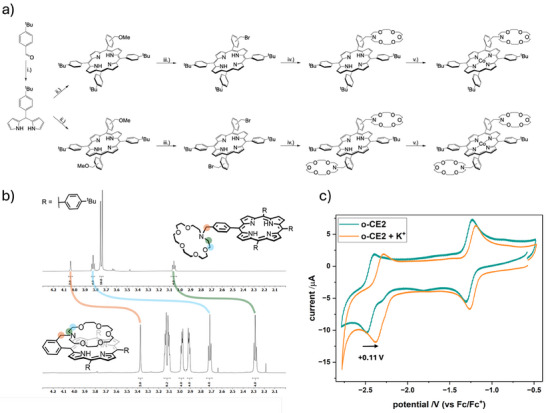
(a) Synthetic route of **p‐CE**, **o‐CE**, **p‐CE2**, and **o‐CE2**. (i) pyrrole, TFA; (ii) 4‐(metoxymethyl)benzaldehyde, BF_3_·OEt_2_, 2,3‐Dichloro‐5,6‐dicyano‐1,4‐benzoquinone, or 2‐(metoxymethyl)benzaldehyde, propionic acid; (iii) HBr in acetic acid; (iv) 1,4,10,13‐tetraoxa‐7,16‐diazacyclooctadecane, K_2_CO_3_, or NaHCO_3_; (v) Co(OAc)_2_·4H_2_O; (b) Display of the crown‐ether NMR‐signal splitting/shift of **H2(p‐CE)** (top) and **H2(o‐CE)** (bottom); (c) Cyclic voltammogram under Ar of **o‐CE2** with (orange) and without (green) added potassium ions.

In this work, we present the design and synthesis of four novel cobalt porphyrin molecules decorated with 1‐aza‐18‐crown‐6 (CE) moieties (for detailed synthesis and characterization see Supporting Information), coupled *via* a CH_2_‐bridge to the TPP base (Figure 3a), to investigate the impact of crown‐ethers on eCO_2_R, using **tBu4** as a reference compound. The porphyrin ring closure was based on the method established by Lindsey, for the *para*‐methoxy porphyrins and Adler–Longo, for their *ortho* analogues, using 2‐/ 4‐(methoxymethyl)benzaldehyde and 5‐(4‐*tert*‐Butylphenyl)dipyrromethane, which was synthesized out of freshly distilled pyrrole and 4‐*tert*‐Butylbenzaldehyde, using catalytic amounts of trifluoracetic acid (TFA) [36, 37, 38]. Since the bis(*ortho*‐methoxymethyl)‐porphyrin can exist as an αα‐ and αβ‐atropo‐isomer, the overall yield of the desired αβ‐isomer was lower compared to its *para* analogue, which does not exhibit atropisomerism. To introduce the crown ether moiety, the methoxymethyl‐porphyrins were deprotected with hydrogen bromide in acetic acid. The resulting bromomethyl porphyrins were reacted with 1‐aza‐18‐crown‐6, in the presence of potassium carbonate or sodium bicarbonate [39]. In the final step, the free base porphyrins were reacted with Co(OAc)_2_·4H_2_O in a chloroform/methanol mixture to yield the cobalt(II)‐loaded catalysts **p‐CE**, **o‐CE**, **p‐CE2**, and **o‐CE2**.

In contrast to the *para*‐substituted crown ether moieties in **p‐CE** and **p‐CE2**, which are spatially separated from the porphyrin metal center, the *ortho* analogues, **o‐CE** and **o‐CE2**, can adopt folded conformations over the macrocycle, enabling direct second sphere interactions with the cobalt center. The structural difference is evident in the free‐base (metal‐free ligand) forms of both the mono and bis crown‐ether derivatives, as illustrated for the mono analogues H2(**p‐CE**) and H2(**o‐CE**) in Figure 3b. In the proton NMR spectra (for complete spectra see SI), the *ortho*‐CE derivatives display increased signal splitting, indicative of a less symmetric environment compared to the *para*‐CE analogues. Moreover, the signals of the *ortho*‐CE derivatives are high field shifted relative to those of the *para*‐analogues, suggesting that the CE moieties are positioned within the diatropic ring current field of the aromatic porphyrin macrocycle (Figure 3b) [40, 41].

### 2.2 Homogenous Electrochemistry

To determine the redox properties of the catalysts, cyclic voltammetry measurements were performed in a one‐compartment cell using a platinum wire as the counter electrode, a silver wire as the pseudo‐reference electrode, and a glassy carbon disk as the working electrode. All measurements were carried out in 0.1 M NBu_4_PF_6_/DMF (dry) as the electrolyte solution and were referenced against the Fc/Fc^+^ redox couple. To probe a possible cation effect associated with the crown ether moieties, additional measurements were conducted with 5 equivalents of KPF_6_ added to the electrolyte (for cyclic voltammograms of the catalysts see Figures S1–S5). To maintain a constant ionic strength, the corresponding amount of NBu_4_PF_6_ was adjusted accordingly. Comparison of the Co^I/0^ redox potential (related to the catalytic CO_2_ reduction process, Figures S1–S5 and S6, as a control) of all studies complexes reveals only minor differences in their redox properties (Table 1). This indicates that neither the number nor the positioning of the crown ether substituents significantly influences the electronic properties of the cobalt center.

**TABLE 1:** Redox potentials (vs. Fc/Fc^+^) of cobalt porphyrin catalysts in dry DMF in the absence and presence of potassium ions.

			+5 eq. K^+^	
Complex	*E*(Co^II/I^) [V]	*E*(Co^I/0^) [V]	*E*(Co^II/I^) [V]	*E*(Co^I/0^) [V]
**tBu4**	−1.25	−2.41	−1.25	−2.41
**p‐CE**	−1.23	−2.39	−1.20	−2.34
**p‐CE2**	−1.24	−2.41	−1.22	−2.36
**o‐CE**	−1.23	−2.40	−1.22	−2.35
**o‐CE2**	−1.24	−2.41	−1.20	−2.30

However, upon addition of potassium ions, an anodic shift of the Co^I^/Co^0^ redox couple is observed for the crown‐ether containing catalysts. Notably, **tBu4** exhibits no detectable changes in its redox properties. For the mono‐crown ether complexes **p‐CE** and **o‐CE**, the anodic shift amounts to +0.05 V. A similar shift of +0.05 V is observed for the bis‐crown ether analogue **p‐CE2**. In contrast, **o‐CE2** exhibits a more pronounced shift of +0.11 V (Figure 3c and Table 1), indicating that the effect is sensitive to the number and position of the crown‐ether moieties. This tendency suggests an electronic influence induced by the coordination of potassium ions to these crown‐ether substituents. Since these substituents are electronically decoupled from the π‐system of the porphyrinic macrocycle, the effect can be attributed to secondary‐sphere interactions rather than direct inductive effects as observed for other CE substituted porphyrin complexes [27, 42]. For **o‐CE** and **o‐CE2**, the potassium‐bound crown ether can sterically approach the cobalt centre and thereby influence its redox potential more directly. In contrast, for the *para*‐substituted analogues **p‐CE** and **p‐CE2**, such direct interaction is geometrically precluded; instead, the crown ether may fold over one of the phenyl rings as it is observed for similar systems [43, 44]. Leading to a slight withdrawal of electron density from the macrocycle and thus indirectly affecting the cobalt center, resulting in a less pronounced shift.

Cobalt porphyrins are only rarely reported as homogeneous eCO_2_R catalysts, consistent with their intrinsically poor performance under such conditions [3, 23]. Controlled potential electrolysis (CPE), supported by pre‐ and post‐electrolysis CV analysis, likewise reveals negligible turnover numbers (TONs) and turnover frequencies (TOFs) for our crown‐ether–substituted complexes, with selectivity shifted toward hydrogen evolution (for details see Supporting Information discussion; Figures S7–S9), in stark contrast to their high activity when immobilized on a cathode (Figure 1b and sections below). Notably, the presence of K^+^ markedly enhances the catalytic performance of **o‐CE2** (Figures S8b and S9a), underscoring the key promoting role of alkali‐metal–mediated secondary‐sphere interactions, which becomes fully expressed within the zero‐gap electrolyzer architecture (vide infra).

### 2.3 Heterogenous Electrocatalysis at Low Current Densities ≤100 mA/cm^2^

To achieve the heterogenization of the presented cobalt complexes a catalytic ink consisting of the respective porphyrin complex, carbon black (ENSACO 250G) and an ionomeric binder (Sustainion XA‐9) dispersed in isopropanol was drop casted onto a gas diffusion layer (carbon cloth) until the desired complex loading was reached (0.5 mg/cm^2^ complex and 1 mg/cm^2^ carbon black). A detailed procedure can be found in the Supporting Information.

**FIGURE 4:**
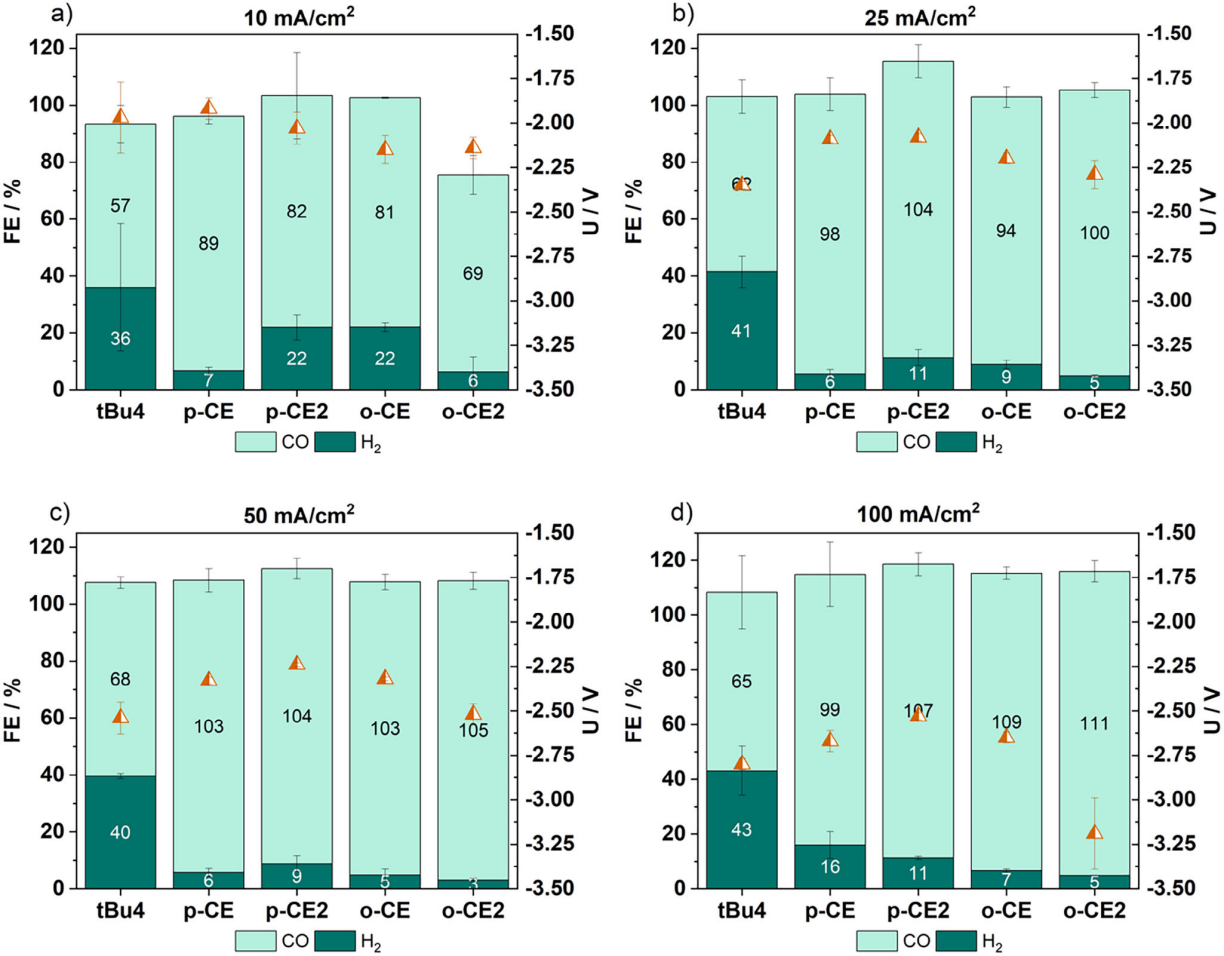
Bar diagram of the achieved FE_CO_ (light green)/FE_H2_ (dark green), exact values are given in numbers within the bars and the corresponding averaged cell voltages (orange triangle) of **tBu4**, **p‐CE**, **p‐CE2**, **o‐CE**, and **o‐CE2@GDE** after 30 min consecutive electrolysis at (a) 10 mA/cm^2^; (b) 25 mA/cm^2^; (c) 50 mA/cm^2^; (d) 100 mA/cm^2^. Total FE values >100% are caused by small air leakages within the *inline* GC set‐up.

Hence, the manufactured electrodes were implemented into an in‐house built 2 cm^2^ ZGE as previously described (Figure 1) [45]. The five catalysts have been tested under commonly applied conditions for heterogenized molecular complexes to ensure a comparability to previously reported systems [7, 11, 12, 13, 25, 46]. Thus, the ZGE was equipped with a nickel foam anode and an anion exchange membrane (here PiperION), which was soaked in 1 M KOH prior to use. During electrolysis 1 M KOH was cycled as anolyte with a speed of 20 mL/min and the cathode was fed with humidified CO_2_ gas at a flowrate of 20 mL/min. Following a short electrochemical electrode conditioning (details in Supporting Information) the electrolysis was consecutively performed at current densities of 10, 25, 50, and 100 mA/cm^2^ for 30 min each (Figures 4 and S10). The gas composition of the outlet stream was analyzed every 30 min *via* online gas chromatography.

At first, the **tBu4** complex was implemented into a GDE (**tBu4@GDE**) and directly showed an improved performance toward eCO_2_R, reaching a FE_CO_ of 60% at an applied current density of 100 mA/cm^2^ (Figure 4d), outperforming the unsubstituted **CoTPP**, which only reaches a FE_CO_ of 38% at the same current density [13]. This selectivity was further improved when the CE containing porphyrins at **GDE (p‐CE**, **p‐CE2, o‐CE**, or **o‐CE2@GDE)** were applied in the ZGE. All CE containing cobalt porphyrins show an even more enhanced activity for CO generation, likely caused by the binding of crossover K^+^ ions from the anode side by the CEs, which can stabilize the reduction intermediates, reaching the highest selectivity for CO of 96% with **o‐CE2@GDE** at 100 mA/cm^2^ (Figure 4d) [33, 47]. Obviously, the anodic shift of the Co^I/0^ redox couple in the presence of K^+^ observed for **o‐CE2** (Figure 3c), with two CE moieties capable of folding over the catalytic cobalt center, appears to be particularly beneficial for selective eCO_2_R. Interestingly, the number of active sites, which were determined *via* CV measurements of the catalyst coated GDEs are not proportional to the catalytic activity (Figures S11–S15) indicating that the local environment of the catalyst plays a more decisive role for efficient eCO_2_R in ZGEs [48].

Both performance parameters, FE_CO_ as well as cell voltage, clearly indicate that the positioning of the CE has a bigger impact on the electrolysis than the number of CEs present in the ligand. Comparison of the bis‐CE functionalized catalyst with its mono‐substituted counterpart shows that **o‐CE2** exhibits a slightly enhanced CO selectivity over **o‐CE** across all investigated current densities. This small improvement is likely attributed to its ability to coordinate two potassium cations in close proximity to the cobalt center thereby strengthening the stabilization of key intermediates. In contrast, the para‐substituted porphyrins display an opposite trend: **p‐CE** shows higher activity than **p‐CE2** at lower current densities, and their FE_CO_ values become comparable at 75 mA/cm^2^. Notably, **p‐CE2** exceeds the mono‐substituted variant only at the highest current density of 100 mA/cm^2^. Because the CE moieties are located farther from the cobalt center in the para‐substituted complexes, the local cation concentration effects are expected to be weaker than in the ortho analogues, rendering the benefit of a second CE less pronounced at low current densities. The enhanced performance of the bis‐CE variants at higher current densities may additionally reflect the higher density of cation‐binding sites, which becomes increasingly relevant at higher current densities, whereas at lower current densities a single CE appears sufficient to saturate the local cation concentration.

When the CE moieties are integrated in the *ortho* position (**o‐CE2@GDE**), the measured cell voltage is 0.63 V more negative compared to the *para*‐positioned analogue **p‐CE2@GDE**, which only reaches a selectivity of 90% (Figure 4d). The observed difference in cell voltage is likely attributable to the intrinsic electrochemical properties of the catalyst coated GDE: CV measurements of **o‐CE2@GDE** revealed the most negative reduction potential of −0.69 V versus Ag/Ag^+^ while all other CE bearing complexes exhibited similar reduction potentials of around −0.4 V versus Ag/Ag^+^ (Figure S16).

To verify that a covalent attachment of the CE to the ligand scaffold is needed to perform the most efficient catalysis, a set of experiments was performed in which **tBu4@GDE** accompanied with two equivalents of 1‐aza‐18‐crown‐6 was deposited on a GDE and tested under identical conditions (Figure S10b). Notably, in comparison to the pure **tBu4@GDE** containing electrode, the **tBu4**/CE impregnated one generated larger amount of CO, increasing the FE_CO_ to 80%. This effect again demonstrates the positive impact of cation migration through the membrane on CE bearing GDEs. Nevertheless, it cannot reach the FE_CO_ achieved by the four cobalt complexes synthesized herein, indicated that a local cation source, that is, it's close proximity to the catalytic center, is beneficial to the eCO_2_R activity.

As CEs are well known to generally bind all alkali cations, and it was shown that the type of alkali cation strongly influences the eCO_2_R in ZGEs, 1 M NaOH and 1 M CsOH have been tested as anolyte as well (Figure S17) [35, 47]. Due to the higher synthetic yields, **p‐CE2@GDE** was chosen for the following in depth studies, since it can capture the same number of cations as the best performing **o‐CE2@GDE**. Changing the anolyte led to a decreased selectivity for CO generation at 100 mA/cm^2^, yielding values of 72% and 83% for NaOH and CsOH, respectively. This is in line with the higher binding affinity of K^+^ to the CE compared to Cs^+^ and Na^+^ (Figure S17) [47]. To verify that the ligand scaffold is required for efficient catalysis and that the eCO_2_R is not catalyzed by cobalt salts, control experiments were conducted using electrodes with the same cobalt loading, prepared from cobalt acetate tetrahydrate as the metal source. Under the given conditions, these electrodes produced only hydrogen (Figure S18).

### 2.4 *Post Mortem* Analysis After Electrolysis at Low Current Densities ≤100 mA/cm^2^

One of the most crucial debates when immobilizing molecular complexes concerns the stability of the complex at elevated current densities: is the molecular system serving as catalyst or as a precursor for, that is, nanoparticles? [7, 12, 13] To answer this question for the cobalt complexes presented herein, a series of analytical techniques (UV/vis/NIR spectroscopy, Scanning Electron Microscopy (SEM) coupled with Energy‐dispersive X‐ray spectroscopy (EDX), XPS and X‐ray computer tomography (CT)) were combined in the form of pre‐ and post‐electrolysis analysis of the utilized GDEs to provide the broadest possible picture, since in situ spectroscopy in ZGEs is limited. To analyze the complex stability using UV/vis/NIR spectroscopy a small part of the GDE was added to DMSO and sonicated for 10 min to extract the complex from the catalyst layer. After filtration of the suspension over Celite, qualitative UV/vis/NIR spectra were recorded and differences between the electrodes before and after electrolysis were compared.

**FIGURE 5:**
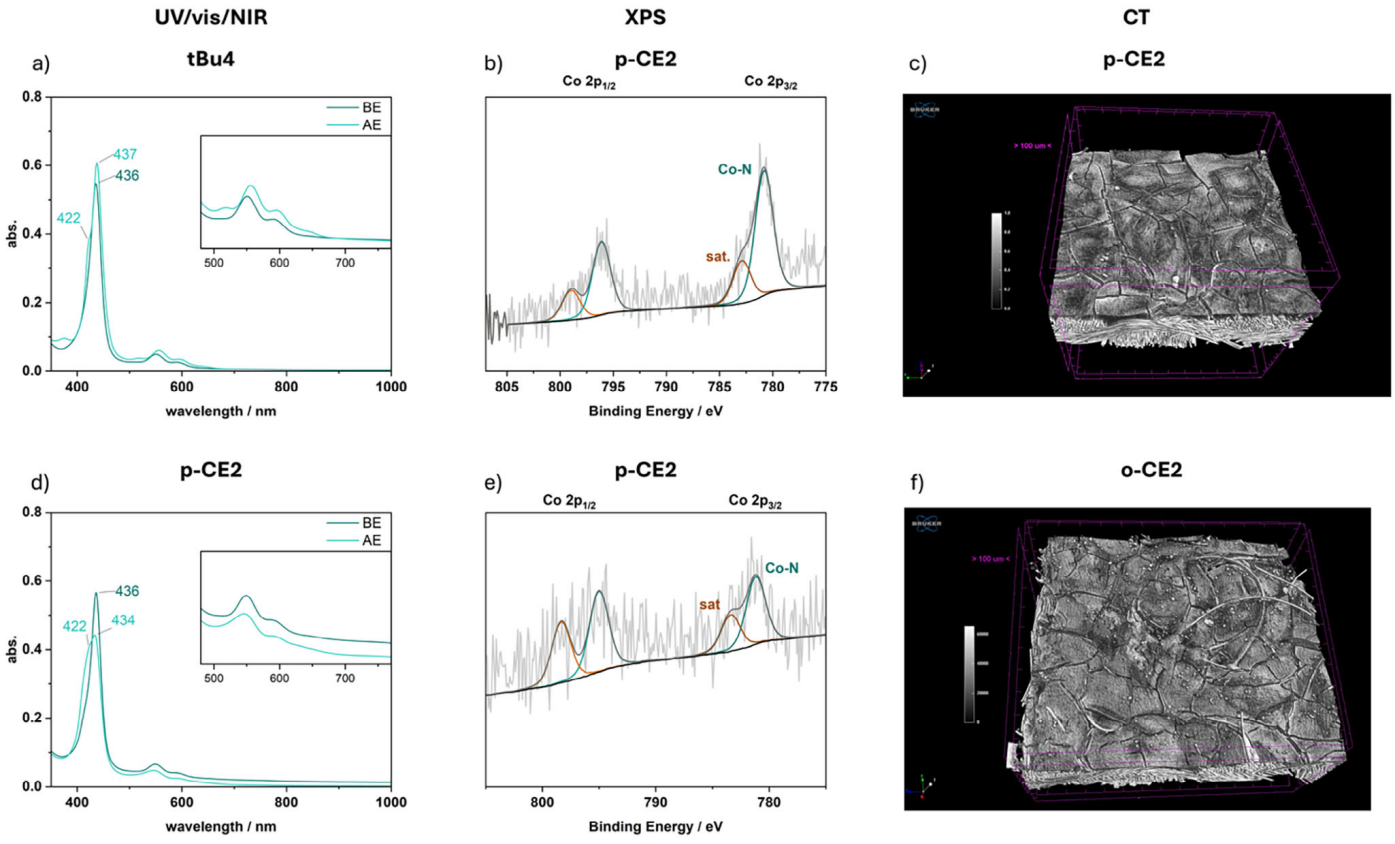
GDE Analysis after catalysis at r.t.: UV/vis/NIR spectra of redissolved complexes from GDEs of (a) **tBu4** and (d) **p‐CE2** before‐ (BE) and after‐electrolysis (AE) in DMSO; Co 2p orbital XPS spectra of a **p‐CE2** containing GDE (b) before and (e) after electrolysis; CT images of pristine GDEs containing (c) **p‐CE2** or (f) **o‐CE2**.

The first noticeable change in structure of the complex is already visible in the spectra recorded before electrolysis: the cobalt center already oxidizes to Co(III) during the GDE fabrication process, as it can be assigned using the obtained results from the UV/vis spectro‐electrochemical measurements (Figures 5, S19, and S20). Additional UV/vis/NIR experiments from different stages of the electrode preparation process indicate that the oxidation occurs during the drop‐casting step (Figures S21–S25). The four CE‐bearing complexes remain stable during electrolysis, as indicated by the UV/vis/NIR spectra, with the Co(II) species being retained, as evidenced by the Soret‐band shifting to shorter wavelengths in the post‐electrolysis spectra. Only the non‐CE bearing **tBu4** shows a partial decomposition as evidenced by the characteristic four Q‐bands of a free tetraphenyl porphyrin in the post‐electrolysis spectrum (inset in Figures 5a and S19). SEM/EDX and XPS analysis revealed that no remaining Co species were left after sonication on the GDE surface, except small traces encapsulated in KOH or in the Celite filter after filtration (Figures S26–S29).

Further SEM/EDX images have been taken of pre‐ and post‐electrolysis **tBu4@GDEs** and **p‐CE2@GDEs** (Figures S30–S37). Regarding the pristine electrode of **p‐CE2@GDE** a rather layered and cracked surface structure of the catalyst is observed, whereas the surface structure of the **tBu4@GDE** electrode appears smoother, comprising bigger particle agglomerates with lengths around 10 µm. The observed larger and more porous surface of **p‐CE2** electrodes accompanied by the smaller particle size, potentially leads to its higher activity due to a higher surface area and beneficial mass transport. Since **o‐CE2** exhibits even higher activity, GDEs coated with **o‐CE2** and **p‐CE2** were analyzed using CT, showing that the distribution of small particle agglomerates is even more extensive for the *ortho*‐complex, which may enhance its catalytic activity (Figures 5c,f and S38–S41). In case of the post‐electrolysis GDEs, the overlap between Co and nitrogen in the EDX maps is much less pronounced for **tBu4** than for **p‐CE2,** for which a clear elemental overlap is retained after electrolysis (Figures S42,S43).

Lastly, the measured Co 2p XPS spectra are also in line with the above findings (Figures 5b,e and S42–S45). On the surface of the pristine electrodes sharp signals for the Co(III) complexes accompanied by their satellite peaks are visible at binding energies of 780.0 and 780.8 eV for **tBu4** and **p‐CE2,** respectively (Figures 5b, S42a, and S43a). Additionally, an iminic Co−N species is observed in the N 1s spectrum of both complexes (Figures S42b and S43b) [13, 49, 50, 51]. In the case of **tBu4,** no Co or iminic N is observed on the electrode surface after electrolysis, whereas the spectra of **p‐CE2** remain unaltered (Figures S44 and S45). Thus, the incorporation of CEs does not only increase the activity toward eCO_2_R in ZGEs, but also drastically improves its stability.

### 2.5 Heterogenous Electrocatalysis at High Current Densities ≥300 mA/cm^2^

In order to approach industrially relevant operating conditions, which have been strongly limited in the community until now, experiments at elevated current densities of up to 500 mA/cm^2^ were performed, representing a unique example in the literature [7, 8, 10]. All following experiments were performed at 60 °C using a humidified CO_2_ gas stream (50 mL/min, 80% relative humidity) and an IrO_2_ (1 mg/cm^2^) coated titanium felt as anode. Like before, besides **tBu4**, the **p‐CE2** complex was chosen for parameter studies due to its higher synthesis yields. Optimized conditions for molecular complexes in ZGEs, previously described by Apfel et al., were applied. Accordingly, a 0.1 M CsOH anolyte was used to reduce the likelihood of carbonate formation, together with an anion‐exchange membrane conditioned in KOH and rinsed with ultrapure water prior to electrolysis [12, 17, 18].

**FIGURE 6:**
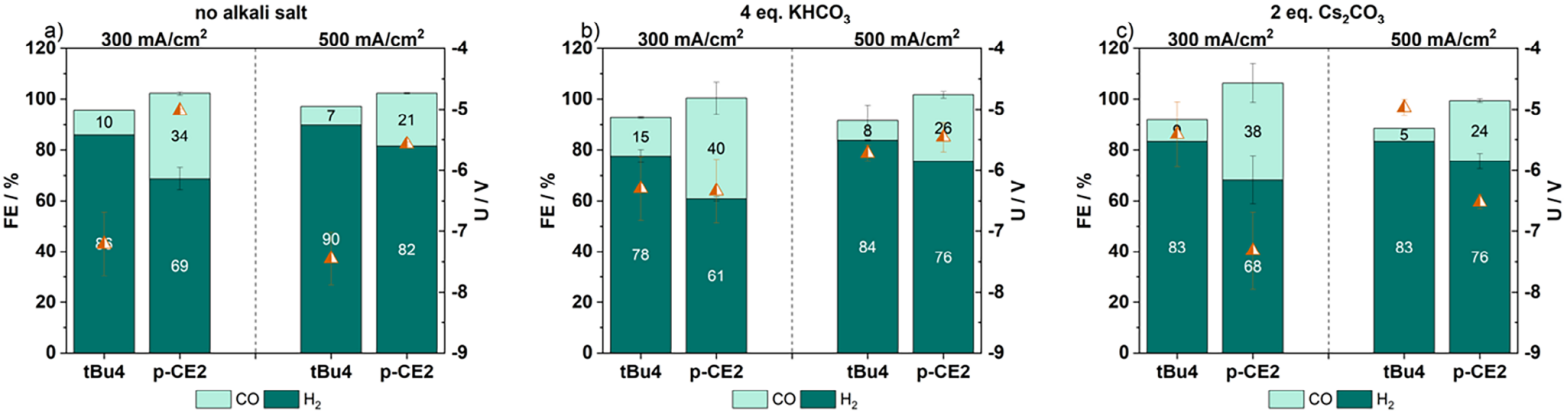
Bar diagram of the achieved FE_CO_ (light green)/FE_H2_ (dark green), exact values are given in numbers within the bars and the corresponding averaged cell voltages (orange triangle) of **tBu4@GDE** and **p‐CE2@GDE** after 30 min electrolysis at 300 and 500 mA/cm^2^ with GDEs comprising either (a) no alkali salt addition; (b) 4 eq. KHCO_3_; (c) 2 eq. Cs_2_CO_3_. Total FE values >100% are caused by small air leakages within the *inline* GC set‐up.

Probably due to the high instability of **tBu4@GDE** under these conditions, experiments reached a cell voltage of −10 V, in five out of six cases, leading to abortion of electrolysis. In the remaining experiment, a low FE_CO_ of 10% at 300 mA/cm^2^ was reached, further indicating a collapse of reactivity (Figures 6a, S46, and S47). UV/vis/NIR analysis supported the progressing decomposition of the pristine complex as the shift of the Soret‐ and Q‐bands is not in accordance with intact complex structure (Figure S48). In contrast **p‐CE2@GDE** showed stable cell voltages in single experiments, however with a deviation of 4 V between the different trials (Figure S49). Just as for the experiments at r.t. the FE_CO_ increased compared to **tBu4@GDE** remarkably, reaching FE_CO_ values of 34% and 21% at 300 and 500 mA/cm^2^, respectively (Figure 6a). Most impressively, no decomposition of the complex is observed using UV/vis/NIR analysis (Figure S48). We hypothesize that the reasons for the high and unstable voltages might have been caused by the low number of cations present in the system, due to low conductivity at elevated current density [52].

Therefore, in the next experiments, the alkali cation concentration was increased directly within the catalytic layer, as this has previously been shown to enhance the eCO_2_R performance of immobilized molecular complexes [35]. Consequently, we added alkali cations to the GDEs catalytic layer *via* the addition of KHCO_3_ (4 eq.) and Cs_2_CO_3_ (2 eq.) to the catalytic ink. These alkali ions have shown a good performance as part of the anolyte at r.t., as well as the highest activation of CO_2_ in literature reports [33, 34, 35]. The (bi)carbonate salts were chosen since the counterion is ubiquitous in the GDE and is not expected to interfere with the catalyst. For both cobalt porphyrins the electrocatalytic performance was improved in terms of FE_CO_ and cell voltage (Figure 6b,c). Especially the addition of KHCO_3_ to **p‐CE2@GDE** resulted in a remarkable FE_CO_ of 40% at 300 mA/cm^2^ (Figure 6b). Nevertheless, the FE_CO_ decreased by 15% at 500 mA/cm^2^. The increased FE_CO_ compared to **tBu4@GDE** might be attributed to a stabilization of the Co(II) species by the alkali cations. Indeed, the UV/Vis/NIR spectra of **p‐CE2@GDE** indicate the presence of this species before electrolysis and remain essentially unchanged after electrocatalysis (Figure S48). As for the measurements at r.t., electrolysis performed with GDEs comprising cobalt acetate tetrahydrate did not show eCO_2_R activity, demonstrating that the complex structure is required to perform the reduction of CO_2_ to CO (Figure S18).

### 2.6 Electrolyte Influence at High Current Densities ≥300 mA/cm^2^

**FIGURE 7:**
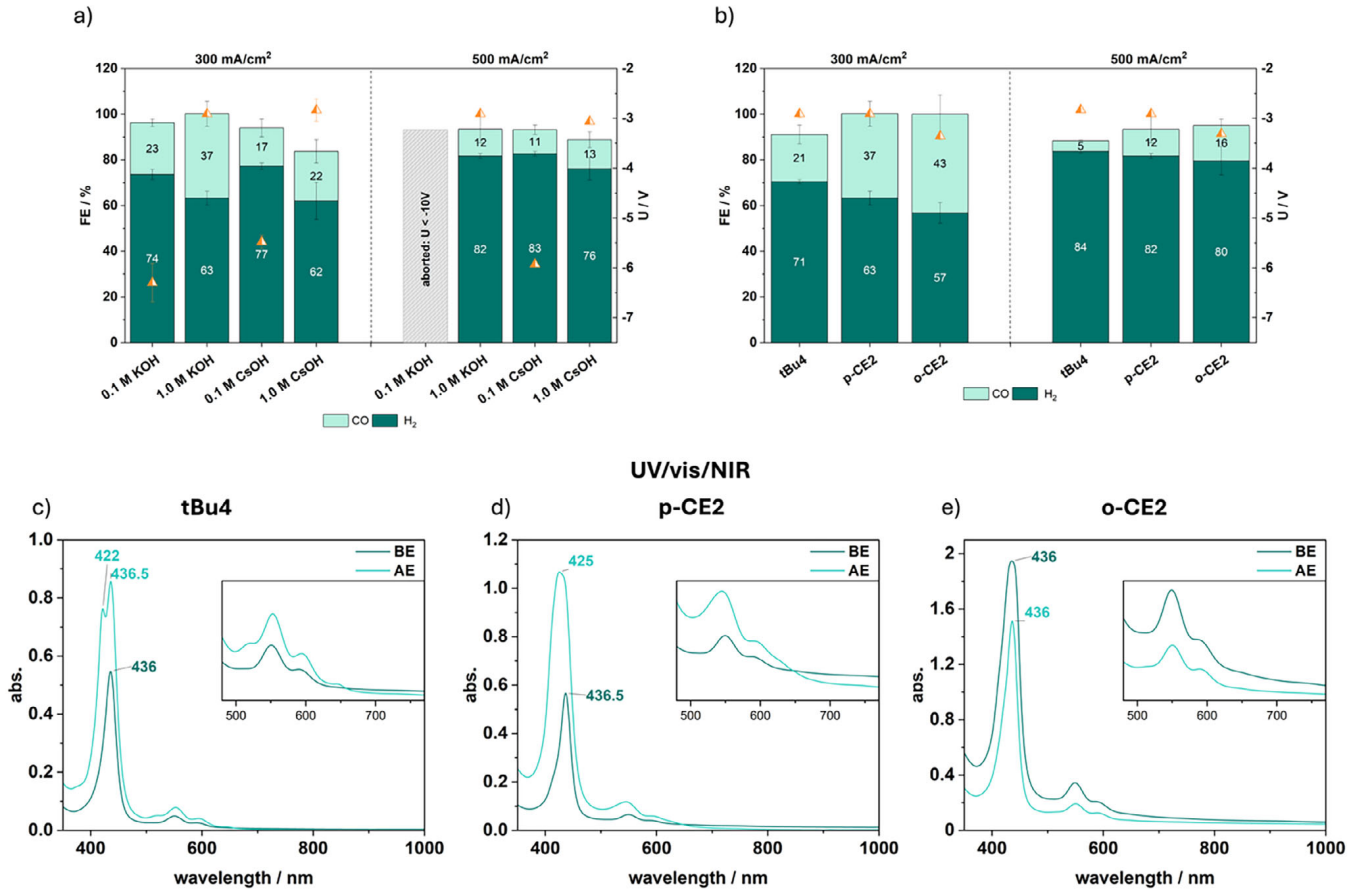
Bar diagram of the achieved FE_CO_ (light green)/FE_H2_ (dark green), exact values are given in numbers within the bars and the corresponding averaged cell voltages (orange triangle) (a) of the **p‐CE2@GDE** after 30 min electrolysis at 300 and 500 mA/cm^2^ in combination with the given anolyte and (b) of **tBu4**, **p‐CE2**, and **o‐CE2@GDE** after 30 min electrolysis at 300 and 500 mA/cm^2^ in combination with 1 M KOH as anolyte. Total FE values >100% are caused by small air leakages within the *inline* GC set‐up; UV/vis/NIR spectra in DMSO of the pre‐ and post‐electrolysis GDEs of (c) **tBu4**, (d) **p‐CE2**, and (e) **o‐CE2**.

Even though a local increase of cations already showed an improved eCO_2_R activity, the obtained cell voltages still need to be finetuned to more energy efficient values. For further optimization of the ion balance, **p‐CE2@GDE** was chosen as catalyst. At first, the ion concentration of the anolyte was increased to 1 M CsOH, and the implemented membrane, soaked in CsOH, was not washed prior to use (Figures 7a and S50). Immediately a positive impact on the cell voltage was observed, resulting in averaged values of −3 V at 500 mA/cm^2^, however, a FE_CO_ of only 13% is achieved (Figure 7a). Even though CsOH is typically employed in eCO_2_R at high current densities, as it is less prone to form carbonate salts compared to KOH, KOH was nevertheless tested here at concentrations of 0.1 and 1 M due to the CEs’ binding affinity for K^+^ [17, 18, 47]. At the 0.1 M concentration, the electrolysis was aborted due to high cell voltages (−10 V) while applying a current density of 500 mA/cm^2^ (Figure S50). 1 M KOH as anolyte resulted in FE_CO_ of 37%, comparable to those achieved using KHCO_3_ as additive on the cathode (Figure 7a), but the cell voltage shows a noteworthy improvement to averaged values of −3 and −2.9 V at 300 and 500 mA/cm^2^, respectively. The rapidly decreasing FE_CO_ at 500 mA/cm^2^ is likely caused by a combination of GDE flooding due to the enhanced formation of carbonate salts as well as catalyst degradation [17, 18].

Even though UV/vis/NIR analysis of the GDE revealed that non‐decayed complex is present on the GDE and no traces of free ligand are observed, XPS analysis showed signs of degradation (Figures 7d and S51). Images taken *via* SEM/EDX have shown that cobalt‐based particles with lengths above 10 µm have agglomerated during catalysis, whereas an overlap with nitrogen is still observed in EDX (Figures S52 and S53). Post‐mortem XPS analysis of the GDE, however, indicates that these particles are mainly a mixture of elemental cobalt and Co(OH)_2_, as the spectra of the Co 2p orbital matches these species (Figure S51a) [53]. Nevertheless, it should be considered that **p‐CE2** accounts for only a small portion of the GDE surface, and since XPS is a very surface‐sensitive technique, the Co 2p signal of the complex might be obscured. A clear iminic N‐Co peak in the N 1s spectrum also indicates stable complex left on the surface (Figure S51b). This GDE sample serves as an excellent example to show that a single type of analysis, such as UV/vis/NIR spectroscopy, is not sufficient to draw definitive conclusions about the stability of molecular complexes in ZGEs. The combination of as many complementary techniques as possible is required to make reliable assessments.

To investigate if the comparably low FE_CO_–with respect to measurements at low current densities–is caused by the partial catalyst transformation to other cobalt species, the gas composition of the outlet gas stream was additionally analyzed after 5 min and 15 min. Indeed, a decay of FE_CO_ over the time span from 62% after 5 min to 37% after 30 min (Figure S54) was observed. These results indicate that while the high intrinsic activity of **p‐CE2@GDE** remains high even at 300 mA/cm^2^, the lower FE_CO_ at this current density arises from progressive catalyst degradation rather than diminished catalytic capability.

### 2.7 Catalyst Comparison at High Current Densities ≥300 mA/cm^2^

Lastly, electrolysis at 300 and 500 mA/cm^2^ employing 1 M KOH as optimal anolyte was performed testing **tBu4** and the highest performing complex **o‐CE2,** to investigate the ligand influence under more industrially relevant conditions (Figures 7b and S55). The non‐CE substituted complex **tBu4** reached a FE_CO_ of 20% at 300 mA/cm^2^, which decreased to 5% at 500 mA/cm^2^, accompanied by an average cell voltage of −2.8 V. In accordance with the previous experiments with **tBu4**, a decomposition toward the free base porphyrin was observed in the recorded UV/vis/NIR spectrum, showing the typical four Q‐bands of a tetraphenyl porphyrin (Figure 7d). In the SEM/EDX images of the post‐electrolysis GDE, cobalt‐based agglomerates exceeding several tens of micrometers are observed. No overlap with nitrogen is detected in the elemental mapping, whereas overlap with oxygen indicates the formation of cobalt oxides or hydroxides (Figures S56 and S57). However, XPS analysis in the Co 2p region did not show any cobalt species. Instead, an intense signal of the K 2p orbital is found at 295 eV, which indicates a highly KOH coated surface that potentially may heavily overlay the Co particles, as also seen in the elemental mapping (Figure S58). Thus, the cobalt species could not be reached *via* XPS. Nevertheless, the missing iminic Co‐N species in the 1 Ns XPS spectrum, also indicates the catalyst degradation (Figure S58b).

At higher current densities, the *ortho*‐substituted **o‐CE2** still shows a higher tendency toward CO generation compared to **p‐CE2**, reaching a FE_CO_ of 43% at 300 mA/cm^2^, accompanied by a more negative cell voltage of −3.7 V (Figure 7b), once again demonstrating the influence of the electrochemical properties of the complex itself. UV/vis/NIR analysis of the post‐mortem GDE verifies that the cobalt porphyrin complex is still observed, indicating a stable catalyst system (Figure 7e). The decreasing FE_CO_ at 500 mA/cm^2^ might, as in the case of **p‐CE2**, be caused by decomposition of the complex into metal particles. To determine if the performance can be increased by variation of the catalyst loading – what can influence the thickness and density of the CL as well we the number of active sites – we performed electrolysis under identical conditions with **o‐CE2** loadings of 0.25 and 0.75 mg/cm^2^ (Figure S59). Therein it was observed that a higher loading, likely accompanied by more intact complex particles achieved the highest FE_CO_ of 50% at 300 mA/cm^2^. Nevertheless, at high current densities, the influence of the ligand is not as decisive as under milder conditions; here, the reaction efficiency is predominantly governed by processes such as carbonate formation, cation crossover, or catalyst aggregation and decomposition.

## 3 Perspectives for Scalable CO_2_ Electrolysis

While the CE‐comprising cobalt porphyrins achieve remarkable results under laboratory scale conditions, ranking them among the best reported state of the art catalysts, their performance under industrial relevant current densities is not yet able to compete with established state of the art systems such as nickel based single atom catalysts, silver complexes or silver nanoparticles. These systems often achieve FE_CO_ values >90% at high current densities of ≥500 mA/cm^2^ while employing comparably cheap and accessible materials [12, 16, 54, 55, 56, 57, 58]. We are conscious, that the herein performed cumbersome, multistep synthesis of CE‐functionalized cobalt porphyrins is not compatible with near‐term large‐scale deployment.

From our viewpoint the presented study is not positioned as a competing catalyst system, but rather as a molecularly well‐defined platform to isolate and quantify the role of local cation concentration on eCO_2_R in ZGEs. Importantly, the CE motif should be viewed as an example for cation scavenging/modulating rather than a mandatory structural element: the underlying design principle – stabilization of catalytically relevant intermediates *via* increasing the local alkali cation concentration – could be translated to simpler and more scalable catalyst architectures. In that regard, we have demonstrated within this work that the sole presence of CEs within the CL improves the eCO_2_R performance without tedious attachment to the actual catalyst. Future efforts will focus on two major aspects:

i) the design and preparation of easier scalable molecular catalyst systems comprising host moieties for alkali cations.
ii) integration of unlinked alkali cation scavengers within the CL of state‐of‐the‐art catalysts to regulate the local cation concentration.

## 4 Conclusion

Aiming for an enhanced catalysis of heterogenized cobalt porphyrin complexes in ZGEs, the four novel cobalt porphyrin complexes **p‐CE**, **p‐CE2**, **o‐CE**, and **o‐CE2** were designed and successfully synthesized. Homogeneous electrochemistry revealed **o‐CE2** experiencing the biggest anodic shift upon K^+^ addition, which highlights the pivotal role of secondary‐sphere interactions and the position of the functional groups in tuning the redox potential of cobalt porphyrins for eCO_2_R. Following homogenous electrochemistry, the cobalt porphyrins were implemented into GDEs for electrolysis in ZGEs. Already at moderate current densities ≤100 mA/cm^2^ and ambient conditions, it was shown that the incorporation of CEs into the porphyrin scaffold drastically improves the eCO_2_R performances in comparison to the non‐CE bearing **tBu4@GDE**, indicating stabilizing interactions of alkali cations in eCO_2_R [28, 32, 33]. A maximum selectivity of 96% for CO at 100 mA/cm^2^ was reached with **o‐CE2@GDE**, which ranks it among the best state‐of‐the‐art systems [7]. Even more, the attachment of CEs does not only enhance the eCO_2_R performance, but it also stabilizes the complex as shown *via* post‐mortem analysis of the GDEs utilizing UV/vis/NIR spectroscopy, SEM/EDX and XPS analysis.

Notably, it was demonstrated that an increase of cation concentration, either local within the catalyst layer on the GDE surface or in electrolyte within the overall system, drives the eCO_2_R toward CO formation at high current densities (300 and 500 mA/cm^2^). Such cation sensitivity arises from the presence of the CE functionality. At an applied current density of 300 mA/cm^2^ a FE_CO_ of 43% is achieved by **o‐CE2@GDE,** which is to date the highest FE_CO_ reported for a non‐noble metal complex in a ZGE at the given current density [7]. As well as for electrolysis at moderate current densities, the CE comprising complexes have proven to be more stable on the GDE during catalysis, according to the used analysis methods, than **tBu4@GDE**. Therefore, the implementation of local cation chelators/scavengers, such as the CE moieties employed herein, represents a promising strategy to enhance both the efficiency and stability of catalysts in application‐oriented systems.

## Conflicts of Interest

The authors declare no conflicts of interest.

## Supporting information




**Supporting File 1**: The authors have cited additional references within the Supporting Information [1–6].

## Data Availability

The data that support the findings of this study are available from the corresponding author upon reasonable request.
