# Supplementary material for: Heavy is the Crown: Crown Ether Modulation of Cobalt Porphyrin CO2 Electroreduction in Zero‐Gap Electrolyzers
Source: Angew Chem Int Ed Engl. 2026 Feb 2;65(11):e25189. doi: 10.1002/anie.202525189 (PMC12970506; doi:10.1002/anie.202525189)
Supplement: Supplementary file 1 — Supporting File 1: The authors have cited additional references within the Supporting Information [1–6]. [file ANIE-65-e25189-s001.pdf]

Supplementary Information

Heavy is the Crown: Crown Ether Modulation of Cobalt Porphyrin CO_2 Electroreduction in Zero-Gap Electrolyzers

Wiebke Wiesner,[a]§ Christian Wilhelm,[b]§ Rahel Cornelia Hoffmann,[b]§ Peter Stahl,[b] Kevinjeorjios Pellumbi,[c] Julia Jökel,[c] Ivana Ivanović-Burmazović*[b] and Ulf-Peter Apfel*[a,c]

[a] W. Wiesner, Prof. Dr. U.-P. Apfel
Lehrstuhl für Anorganische Chemie I
Ruhr-Universität Bochum
Universitätsstr. 150, 44801 Bochum, Germany
E-mail: ulf.apfel@rub.de

[b] C. Wilhelm, R. C. Hoffmann, P. Stahl, Prof. Dr. I. Ivanović-Burmazović
Department Chemie
Ludwig-Maximilians-Universität München
Butenandtstr. 5-13, Haus D, 81377 München, Germany
E-mail: ivana.ivanovic-burmazovic@cup.uni-muenchen.de

[c] Dr. K. Pellumbi, Dr. J. Jökel, Prof. Dr. U.-P. Apfel
Department Electrosynthesis
Fraunhofer-Institut für Umwelt-, Sicherheits- und Energietechnik UMSICHT
Osterfelder Straße 3, 46047 Oberhausen, Germany
E-mail: ulf.apfel@umsicht.fraunhofer.de

Contents

Synthesis2

Homogenous Electrochemistry11

Electrode Preparation11

Cyclic Voltammetry of GDEs12

Heterogenous electrochemistry12

Gas Chromatography13

UV/vis Spectroscopy of redissolved Complexes13

UV/vis/NIR spectroelectrochemistry13

Scanning Electron Microscopy14

X-Ray Photoelectron Spectroscopy14

X-Ray Computer tomography14

Additional Figures14

NMR-spectra41

ESI-MS-spectra53

References55

Synthesis

General materials and methods: Dimethylformamide (DMF, Aldrich 99.9%), tetra-n-butyl ammonium hexafluorophosphate ($[NBu_4]PF_6$, Aldrich 99%) were used as received. All other chemical reagents used in the synthetic route were obtained from commercial sources as guaranteed-grade reagents and used without further purification. **tBu4** and 2-(methoxy-methyl)benzaldehyde were prepared following previously reported procedures.[1–3] The electrospray ionization mass spectrometry (ESI-HRMS) experiments were performed on UHR-TOF Bruker Daltonik maXis plus, an ESI-quadrupole time-of-flight (qToF) mass spectrometer) with an ESI+ method. NMR spectra were recorded with a Bruker Avance Neo 500 spectrometer. The spectrometer operates at 500 MHz for the proton nuclei. The chemical shifts of the NMR spectra are reported in ppm relative to the shift of the standard tetramethylsilane. ^{1}H NMR shifts are calibrated to the residual solvent resonances. In the report of the ^{1}H spectroscopic data, the multiplicity of the signals is abbreviated with s (singlet), d (doublet), t (triplet), and m (multiplet).

General procedure A

Porphyrin **X** (1.0 eq.) was dissolved in DCM in a round bottom flask. Subsequently a hydrogen bromide solution in acetic acid was added. The round bottom flask was connected to two washing flasks, whereas one is filled with 5M $NaOH_{aq.}$ to quench evaporating hydrogen bromide. The reaction solution was stirred overnight at room temperature. To stop the reaction, the solution was quenched with 1M NaOH. The organic layer was washed thrice with H_2O, dried over $MgSO_4$, filtered and the solvent was removed.

General procedure B

In a flask equipped with a reflux condenser, porphyrin **X** (1.0 eq.), 1,4,10,13-tetraoxa-7,16-diazacyclooctadecane (1.1 – 2.1 eq.) and $NaHCO_3$ or K_2CO_3 (2.1 – 4.2 eq.) were dissolved in dry toluene under argon atmosphere. The reaction mixture was stirred for 24 hours at 120 °C. The toluene was removed under reduced pressure and the residue was extracted with DCM. The organic layer was washed thrice with H_2O, dried over $MgSO_4$, filtered and the solvent was removed.

General procedure C

In a small flask equipped with a reflux condenser, porphyrin **3** (1.0 eq.) in $CHCl_3$ was mixed with $Co(OAc)_2{\cdot}4H_2O$ (15 eq.), dissolved in dry MeOH. The reaction mixture was stirred for 24 hours at room temperature. The solvent was removed under reduced pressure. The residue was dissolved in 50 mL DCM, washed thrice with H_2O, dried over $MgSO_4$, filtered and the solvent was removed.

Synthesis of 5-(4-(*tert*-butyl)phenyl)dipyrromethane 1[4]

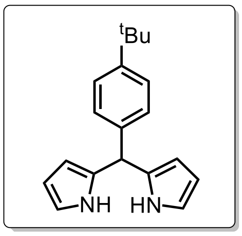

In a 100 mL round-bottom flask, freshly distilled pyrrole (31 mL, 0.45 mol, 15 eq.) was placed, and 4-(tert-butyl)benzaldehyde (5.0 mL, 30 mmol, 1.0 eq.) was added under stirring. The solution was degassed with argon at room temperature for 15 min. After the addition of 0.6 mL TFA, it was stirred for 15 min at room temperature and then quenched with an aqueous NaOH (1.0 M) solution. The organic phase was extracted with DCM and dried over Na_2SO_4. Solvent and excess pyrrole were removed under vacuum, and the resulting brown liquid was purified on a silica plug with DCM. After removal of the solvent under reduced pressure, the brown solid was washed with iso-hexane to give compound **1** as a white solid (5.0 g, 18 mmol, 60% yield).

^{1}H-NMR (500 MHz, RT, $CDCl_3$): δ [ppm] = 7.91 (s, 2H); 7.33 (d, 2H, ^3J = 8.5 Hz); 7.15 (d, 2H, ^3J = 8.1 Hz); 6.70 – 6.68 (m, 2H); 6.17 – 6.15 (m, 2H); 5.95 – 5.93 (m, 2H); 5.45 (s, 1H); 1.31 (s, 9H).

Synthesis of 2 and 3

In a Schlenk flask, 5-(4-(*tert*-butyl)phenyl)dipyrromethane **1** (2.8 g, 10.0 mmol, 1.0 eq.) and 4-(methoxy-methyl)benzaldehyde (1.5 g, 10.0 mmol, 1.0 eq.) were dissolved in DCM under argon atmosphere. Subsequently, $BF_3 \cdot Et_2O$ (0.2 ml) was added and the mixture was stirred for 2 h in the dark. Afterwards, DDQ (3.4 g, 15.0 mmol, 1.5 eq.) was added and the reaction was stirred for 1 h at room temperature. The reaction mixture was filtrated over a silica plug and the solvent was evaporated. The crude product was purified via column chromatography (silica/DCM/hex; 7:3). Product **2** was obtained as purple solid. Product **3** was obtained as a purple side product.

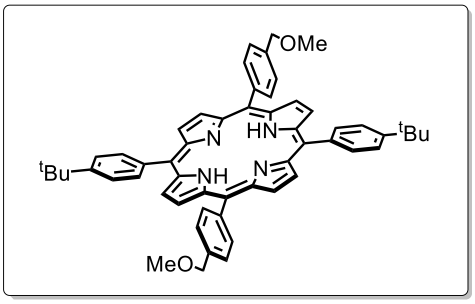

<u>Porphyrin **2**</u>

(0.51 g, 0.62 mmol, 13% yield).

^{1}H-NMR (500 MHz, RT, $CDCl_3$): δ [ppm] = 8.94 – 8.87 (m, 8H, β-pyrrole); 8.24 (d, 4H, ^3J = 8.5 Hz, *o*-aryl-*H*); 8.18 (d, 4H, ^3J = 8.5 Hz, *o*-^{t}Bu-aryl-*H*); 7.78 (d, 4H, ^3J = 8.0 Hz, *m*-^{t}Bu-aryl-*H*); 7.73 (d, 4H, ^3J = 8.0 Hz, *m*-aryl-*H*); 4.81 (s, 4H, Ar-CH_2-OMe); 3.67 (s, 6H, -OCH_3); 1.64 (s, 18H, C(CH_3)$_3$); -2.69 (s, 2H, N-*H*).
^{13}C-NMR (126 MHz, RT, $CDCl_3$): δ [ppm] = 150.7 (*p*-^{t}Bu-aryl-*Cq*); 141.8 (*Cq1*-aryl); 139.3 (*Cq1*- ^{t}Bu-aryl); 137.8 (*p*-aryl-*Cq*); 134.8 (*o*-aryl-*C*); 134.6 (*o*-^{t}Bu-aryl-*C*); 131.3 (β-pyrrol-*C)*; 126.2 (*m*-aryl-*C*); 123.8 (*m*-^{t}Bu-aryl-*C)*; 120.6 (bridg*Cq*-^{t}Bu-aryl); 119.9 (bridg*Cq*-aryl); 75.0 (Ar-*C*H_2-OMe); 58.8 (O-*C*H_3); 35.1 (*C*(CH_3)$_3$); 31.9 (C(*C*H_3)$_3$).

ESI-MS (m/z): [M + Na]$^+$ calcd $C_{56}H_{54}N_4O_2$ for 837.4139, found 837.4137.
UV/Vis (DMSO): λ nm (ε) 421 (171800), 516 (6480), 552 (3780), 592 (2000), 647 (2160).

Porphyrin **3**

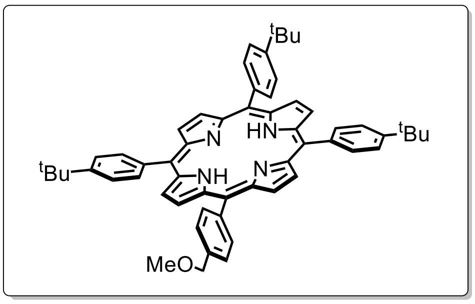

(0.37 g, 0.45 mmol, 9% yield).

^{1}H NMR (500 MHz, $CDCl_3$) δ [ppm] = 8.90 – 8.89 (m, 6H, β -pyrrole-*H*); 8.85 – 8.84 (m, 2H, β-pyrrole-H); 8.23 (m, 2H, *o*-aryl-*H*); 8.17 – 8.15 (m, 6H, *o*-^{t}Bu-aryl-*H*); 7.77 (d, 6H, ^3J = 8.2 Hz, *m*-^{t}Bu-aryl-*H*); 7.73 (d, 2H, ^3J = 7.9 Hz, *m*-aryl-*H*); 4.81 (s, 2H, Ar-CH_2-OMe); 3.66 (s, 3H, -OCH_3); 1.63 (s, 27H, C(CH_3)$_3$); -2.72 (s, 2H, N-H).
^{13}C-NMR (126 MHz, RT, $CDCl_3$): δ [ppm] = 150.6 (*p*-^{t}Bu-aryl-*Cq*); 141.9 (*Cq1*-aryl); 139.4 (*Cq1*- ^{t}Bu-aryl); 137.7 (*p*-aryl-*Cq*); 134.8 (*o*-aryl-*C*); 134.6 (*o*-^{t}Bu-aryl-*C*); 131.1 (β-pyrrol-*C)*; 126.2 (*m*-aryl-*C*); 123.8 (*m*-^{t}Bu-aryl-*C)*; 120.4 (bridg*Cq*-^{t}Bu-aryl); 119.6 (bridg*Cq*-aryl); 75.1 (Ar-*C*H_2-OMe); 58.8 (O-*C*H_3); 35.1 (*C*(CH_3)$_3$); 31.9 (C(*C*H_3)$_3$).

ESI-MS (m/z): [M + Na]$^+$ calcd $C_{58}H_{58}N_4O$ for 849.4503, found 849.4496.
UV/Vis (DMSO): λ nm (ε) 421 (183600), 517 (7180), 553 (3920), 593 (1880), 647 (2220).

Synthesis of 4

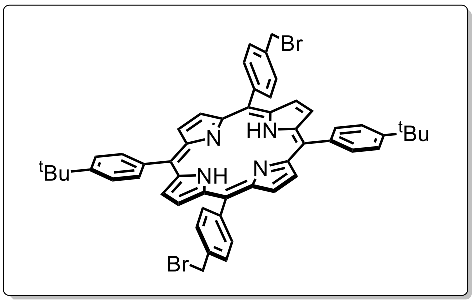

Synthesis following general procedure **A**. Porphyrin **2** (0.37 g, 0.45 mmol, 1.0 eq.), DCM (15 mL) HBr (30 mL, 33% in AcOH). The product was obtained as purple solid (0.36 g, 0.39 mmol, 87% yield).

^{1}H-NMR (500 MHz, RT, $CDCl_3$): δ [ppm] = 8.92 – 8.82 (m, 8H, β-pyrrole); 8.20 – 8.12 (m, 8H, *o*-aryl-*H, o*-^{t}Bu-aryl-*H*); 7.78 – 7.75 (m, 8H, *m*-aryl-*H, m*-^{t}Bu-aryl-*H*); 4.85 (s, 4H, Ar-CH_2-Br); 1.62 (s, 18H, C(CH_3)$_3$); -2.75 (s, 2H, N-*H*).
^{13}C-NMR (126 MHz, RT, $CDCl_3$): δ [ppm] = 150.8 (*p*-^{t}Bu-aryl-*Cq*); 142.6 (*Cq1*-aryl); 139.2 (*Cq1*- ^{t}Bu-aryl); 137.5 (*p*-aryl-*Cq*); 135.1 (*o*-aryl-*C*); 134.6 (*o*-^{t}Bu-aryl-*C*) 131.1 (β-pyrrol-*C*); 127.6 (*m*-aryl-*C*); 123.8 (*m*-^{t}Bu-aryl-*C)*; 120.9 (bridg*Cq*-^{t}Bu-aryl*)*; 119.1 (bridg*Cq*-aryl); 35.1 (*C*(CH_3)$_3$) 33.7 (Ar-*C*H_2-Br); 31.8 (C(*C*H_3)$_3$).

ESI-MS (m/z): [M + H]$^+$ calcd $C_{54}H_{48}N_4Br_2$ for 911.2318, found 911.2327.
UV/Vis (DMSO): λ nm (ε) 421 (189800), 516 (9680), 552 (6120), 592 (4000), 647 (3800).

Synthesis of 5

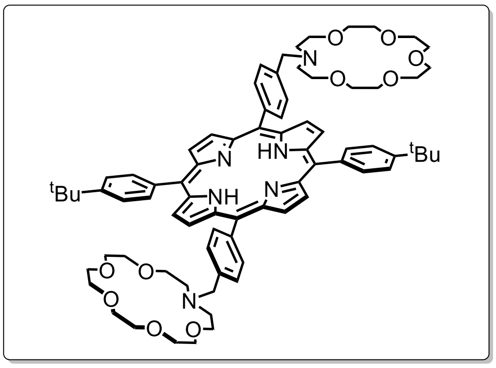

Synthesis following general procedure **B**. Porphyrin **4** (0.29 g, 0.32 mmol, 1.0 eq.), 1,4,10,13-tetraoxa-7,16-diazacyclooctadecane (0.18 g, 0.67 mmol, 2.1 eq.) and K_2CO_3 (0.19 g, 1.35 mmol, 4.2 eq.) and dry toluol (40 mL). The product was obtained as purple powder (0.41 g, 0.32 mmol, 97% yield).

^{1}H-NMR (500 MHz, RT, $CDCl_3$): δ [ppm] = 8.90 – 8.84 (m, 8H, β-pyrrole); 8.17 – 8.14 (m, 8H, *o*-aryl-*H, o*-^{t}Bu-aryl-*H*); 7.79 – 7.72 (m, 8H, *m*-aryl-*H, m*-^{t}Bu-aryl-*H*); 4.05 (s, 4H, Ar-C*H2*-N); 3.83 (t, 8H, 3J = 5.8 Hz, NCH2-C*H2*); 3.74 (d, 32H, 3J = 6.9 Hz, -O-C*H2*-); 3.06 (t, 8H, 3J = 5.8 Hz, N-C*H2*); 1.62 (s, 18H, C(C*H3*)3); -2.73 (s, 2H, N-*H*).
^{13}C-NMR (126 MHz, RT, $CDCl_3$): δ [ppm] = 150.6 (*p*-^{t}Bu-aryl-*Cq*); 140.9 (*Cq1*-aryl); 139.4 (*Cq1*- ^{t}Bu-aryl); 134.7 (*o*-aryl-*C, o*-^{t}Bu-aryl-*C*); 131.1 (β-pyrrol-*C*); 127.3 (*m*-aryl-*C)*; 123.7 (*m*-^{t}Bu-aryl-*C*); 120.4 (bridg*Cq*-^{t}Bu-aryl); 120.1 (bridg*Cq*-aryl); 71.1 – 70.4 (-O-*C*H2-); 60.3 (Ar-*C*H2-N); 54.4 (N-*C*H2); 35.1 ((*C*(CH3)3); 31.9 (C(*C*H3)3).

ESI-MS (m/z): $[M + Na]^+$ calcd $C_{78}H_{96}N_6O_{10}$ for 1299.7080, found 1299.7050.
UV/Vis (DMSO): λ nm (ε) 421 (193600), 517 (7400), 552 (3980), 592 (2040), 647 (2120).

Synthesis of p-CE2

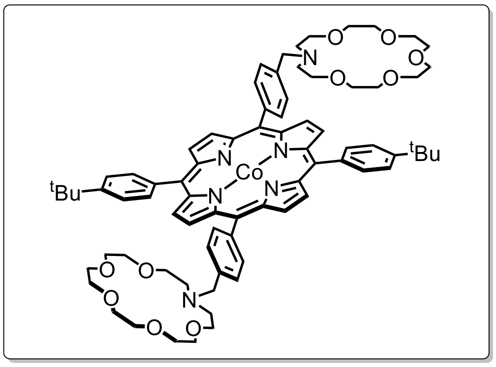

Synthesis following general procedure **C**. Porphyrin **5** (0.19 g, 0.15 mmol, 1.0 eq.) in $CHCl_3$ (50 ml) was mixed with $Co(OAc)_2{\cdot}4H_2O$ (0.41 g, 2.25 mmol, 15 eq.), dissolved in MeOH (15 ml). The product remains as purple solid, yielding 0.16 g (0.12 mmol, 80% yield).

ESI-MS (m/z): $[M + H]^{2+}$ calcd $C_{78}H_{94}N_6O_{10}Co$ for 667.8254, found 667.8253.
UV/Vis (DMSO): λ nm (ε) 418 (184000), 533 (13000).

Synthesis of 6

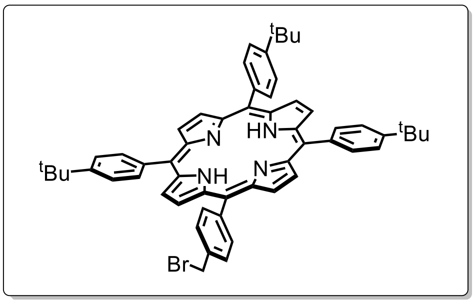

Synthesis following general procedure **A**. Porphyrin **3** (200 mg, 0.24 mmol, 1.0 eq.), DCM (8 mL), HBr (16 mL, 33% in AcOH). The product was obtained as purple solid (0.19 g, 0.22 mmol, 91% yield).

^{1}H-NMR (500 MHz, RT, $CDCl_3$): δ [ppm] = 8.90 – 8.88 (m, 6H, β-pyrrole); 8.82 – 8.81 (m, 2H, β -pyrrole-*H*); 8.21 (d, 2H, ^{3}J = 8.0 Hz, *o*-aryl-*H*); 8.16 – 8.14 (m, 6H, *o*-^{t}Bu-aryl-*H*); 7.79 – 7.75 (m, 8 H, *m*-aryl-*H, m*-^{t}Bu-aryl-*H*); 4.85 (s, 2H, Ar-C*H*$_2$-Br); 1.62 (s, 27H, C(C*H*$_3$)$_3$); -2.74 (s, 2H, N-*H*).
^{13}C-NMR (126 MHz, RT, $CDCl_3$): δ [ppm] = 150.7 (*p*-^{t}Bu-aryl-*Cq*); 142.7 (*Cq1*-aryl); 139.3 (*Cq1*- ^{t}Bu-aryl); 137.3 (*p*-aryl-*Cq*); 135.1 (*o*-aryl-*C*); 134.6 (*o*-^{t}Bu-aryl-*C*) 131.5 (β-pyrrol-*C*); 127.5 (*m*-aryl-*C*); 123.8 (*m*-^{t}Bu-aryl-*C)*; 120.5 (bridg*Cq*-^{t}Bu-aryl*)*; 118.9 (bridg*Cq*-aryl); 35.1 (*C*(CH$_3$)$_3$) 33.8 (Ar-*C*H$_2$-Br); 31.8 (C(*C*H$_3$)$_3$).

ESI-MS (m/z): [M + H]$^+$ calcd $C_{57}H_{55}N_4Br$ for 875.3683, found 875.3678.
UV/Vis (DMSO): λ nm (ε) 421 (180000), 517 (7120), 553 (4380), 593 (2180), 647 (2520).

Synthesis of H2(p-CE) (*7)*

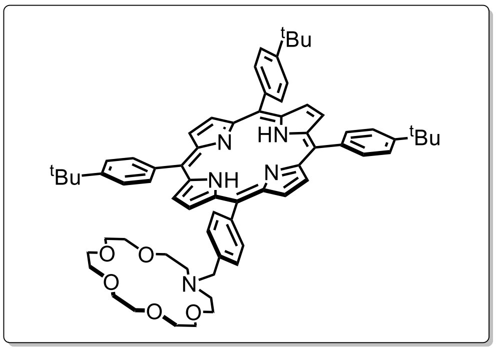

Synthesis following general procedure **B**. Porphyrin **6** (100 mg, 0.11 mmol, 1.0 eq.), 1,4,10,13-tetraoxa-7,16-diazacyclooctadecane (32.0 mg, 0.12 mmol, 1.1 eq.) and K_2CO_3 (33.2 mg, 0.24 mmol, 2.1 eq.) and dry toluol (15 mL). The product was obtained as purple powder (105 mg, 0.10 mmol, 90% yield).

^{1}H-NMR (500 MHz, RT, $CDCl_3$): δ [ppm] = 8.95 – 8.9 (m, 8H, β-pyrrole); 8.21 – 8.18 (m, 8H, *o*-aryl-*H, o*-^{t}Bu-aryl-*H*); 7.8 – 7.73 (m, 8H, *m*-aryl-*H, m*-^{t}Bu-aryl-*H*); 4.03 (s, 2H, Ar-C*H*$_2$-N); 3.83 (t, 4H, 3J = 5.9 Hz, NCH$_2$-C*H*$_2$); 3.75 (d, 16H, 3J = 7.6 Hz, -O-C*H*$_2$-); 3.06 (t, 4H, 3J = 5.9 Hz, N-C*H*$_2$); 1.65-1.64 (m, 27H, C(C*H*$_3$)$_3$); -2.65 (s, 2H, N-*H*).
^{13}C-NMR (126 MHz, RT, $CDCl_3$): δ [ppm] = 150.6 (*p*-^{t}Bu-aryl-*Cq*); 140.9 (*Cq1*-aryl); 139.4 (*Cq1*- ^{t}Bu-aryl); 134.6 (*o*-aryl-*C, o*-^{t}Bu-aryl-*C*); 131.3 (β-pyrrol-*C*); 127.3 (*m*-aryl-*C)*; 123.7 (*m*-^{t}Bu-aryl-*C*); 120.3 (bridg*Cq*-^{t}Bu-aryl); 120.0 (bridg*Cq*-aryl); 71.1 – 70.3 (-O-*C*H$_2$-); 60.3 (Ar-*C*H$_2$-N); 54.3 (N-*C*H$_2$); 35.0 ((*C*(CH$_3$)$_3$); 31.8 (C(*C*H$_3$)$_3$).

ESI-MS (m/z): [M+H]$^+$ calcd $C_{69}H_{79}N_5O_5$ for 1058.6154, found 1058.6165.
UV/Vis (DMSO): λ nm (ε) 421 (189200), 517 (8440), 553 (5840), 593 (3860), 647 (3980).

Synthesis of p-CE

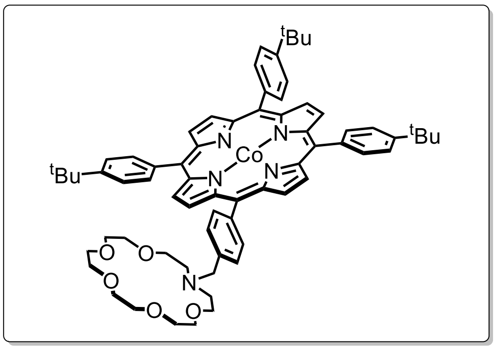

Synthesis following general procedure **C**. Porphyrin **7** (80.0 mg, 0.076 mmol, 1.0 eq.) in $CHCl_3$ (25 ml) was mixed with $Co(OAc)_2{\cdot}4H_2O$ (285 mg, 1.14 mmol, 15 eq.), dissolved in MeOH (8 ml). The product remains as purple solid, yielding 73 mg (0.065 mmol, 86% yield) of **9**.

ESI-MS (m/z): $[M+H]^+$ calcd $C_{69}H_{77}N_5O_5Co$ for 1115.5329, found 1115.5311.
UV/Vis (DMSO): λ nm (ε) 418 (162000), 534 (11340).

Synthesis of 8 and 9

5-(4-(*tert*-Butyl)phenyl)dipyrromethane (2.78 g, 10.0 mmol, 1.0 eq.) and 2-(methoxymethyl)-benzaldehyde (1.50 mg, 10.0 mmol, 1.0 eq.) were dissolved in 120 mL propionic acid and stirred under reflux for 3 h. The solvent was removed under vacuum and washed with hot water. The dark residue was quenched with $NaHCO_3$, extracted with DCM, washed with water and dried over $MgSO_4$. To avoid an overload of the column, the mixture was precleaned by a silica-plug with DCM/Hex (7:3). The crude product was purified via column chromatography (silica/DCM/hex; 7:3). Product **8** was obtained as purple solid. Product **9** was obtained as a purple side product.

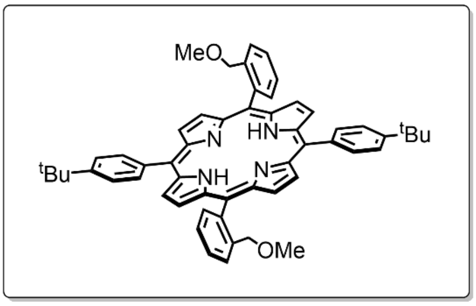

<u>Porphyrin **8**</u>
Isomeric structure α/β (380 mg, 0.47 mmol, 9% yield)
^{1}H NMR (500 MHz, RT, $CDCl_3$): δ [ppm] = 8.87 (d, ^3J = 4.7 Hz, 4H, β-pyrrole); 8.68 (d, ^3J = 4.7 Hz, 4H, β-pyrrole); 8.16 – 8.13 (m, 4H, *o*-^{t}Bu-aryl-*H*); 8.03 (dd, ^3J = 7.4, 1.3 Hz, 2H, *o*-aryl-*H*); 7.94 (dd, ^3J = 8.0, 0.7 Hz, 2H, *m*-aryl-*H*); 7.83 (td, ^3J = 7.7, 1.4 Hz, 2H, *p*-aryl-*H*); 7.78 – 7.75 (m, 4H, *m*-^{t}Bu-aryl-*H*); 7.63 (td, ^3J = 7.5, 1.4 Hz, 2H, *m*-aryl-*H*); 4.13 (s, 4H, Ar-CH_2-OMe); 2.86 (s, 6H, -OCH_3); 1.61 (s, 18H, C(CH_3)$_3$); -2.69 (s, 2H, N-*H*).
^{13}C NMR (126 MHz, RT, $CDCl_3$) δ [ppm] = 150.8 (*p*-^{t}Bu-aryl-*Cq*); 140.4 (*Cq1*-aryl); 140.3 (*o*-aryl-*Cq*); 138.9 (*Cq1*-^{t}Bu-aryl); 134.6 (*o*-^{t}Bu-aryl-*C*); 134.2 (*o*-aryl-*C*); 131.9 (β-pyrrole); 128.8 (*p*-aryl-*C*); 126.5 (*m*-aryl-*C*); 125.6 (*m*-aryl-*C*); 123.8 (*m*-^{t}Bu-aryl-*C*); 120.5 (bridg*Cq*-^{t}Bu-aryl); 117.4 (bridg*Cq*-aryl); 72.9 (Ar-*C*H_2-OMe); 58.3 (O-*C*H_3); 35.1 (*C*(CH_3)$_3$); 31.8 (C(*C*H_3)$_3$).

ESI-MS (m/z): $[M + H]^+$ calcd $C_{56}H_{54}N_4O_2$ for 815.4320, found 815.4335.
UV/Vis (DMSO): λ nm (ε) 420 (248186), 516 (9641), 551 (3845), 591 (2500), 647 (2420).

Porphyrin **9**

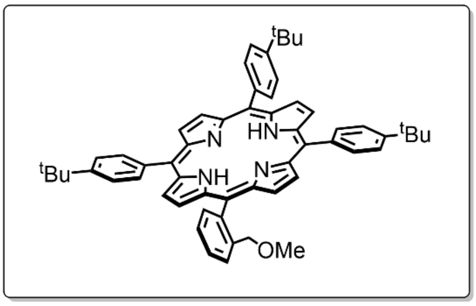

(800 mg, 0.97 mmol, 20 % yield)
^{1}H NMR (500 MHz, RT, $CDCl_3$): δ [ppm] = 8.91 – 8.84 (m, 6H, β-pyrrole); 8.68 (d, ^3J = 4.7 Hz, 2H, β-pyrrole) ; 8.21 – 8.09 (m, 6H, *o*-^{t}Bu-aryl-*H*); 8.07 (dd, ^3J = 7.5, 1.3 Hz, 1H, *o*-aryl-*H*); 7.94 (d, ^3J = 7.9 Hz, 1H, *m*-aryl-*H*); 7.83 (td, ^3J = 7.7, 1.4 Hz, 1H, *p*-aryl-*H*); 7.79 – 7.73 (m, 6H, *m*-^{t}Bu-aryl-*H*); 7.64 (td, ^3J = 7.5, 1.4 Hz, 1H, *m*-aryl-*H*); 4.11 (s, 2H, Ar-C*H*$_2$-OMe); 2.85 (s, 3H, -OC*H*$_3$); 1.62 (d, ^3J = 2.6 Hz, 27H, C(C*H*$_3$)$_3$); -2.70 (s, 2H, N-*H*).
^{13}C NMR (126 MHz, RT, $CDCl_3$) δ [ppm] = 150.7 (*p*-^{t}Bu-aryl-*Cq*); 140.4 (*Cq1*-aryl); 140.4 (*o*-aryl-*Cq*); 139.4 (*Cq1*-^{t}Bu-aryl); 139.2 (*Cq1*-^{t}Bu-aryl); 134.6 (*o*-^{t}Bu-aryl-*C*); 134.2 (*o*-aryl-*C*); 131.3 (β-pyrrol-*C*); 128.7(*p*-aryl-*C*); 126.4 (*m*-aryl-*C*); 125.6 (*m*-aryl-*C*); 123.8 (*m*-^{t}Bu-aryl-*C)*; 120.7 (bridg*Cq*-^{t}Bu-aryl); 120.4 (bridg*Cq*-^{t}Bu-aryl); 117.0 (bridg*Cq*-aryl); 72.9 (Ar-*C*H$_2$-OMe); 58.3 (O-*C*H$_3$); 35.1 (*C*(CH$_3$)$_3$); 31.8 (C(*C*H$_3$)$_3$).

ESI-MS (m/z): [M + H]$^+$ calcd $C_{58}H_{58}N_4O$ for 827.4683, found 827.4695.
UV/Vis (DMSO): λ nm (ε) 420 (237600), 516 (9605), 551 (5543), 592 (3418), 646 (3652).

Synthesis of 10

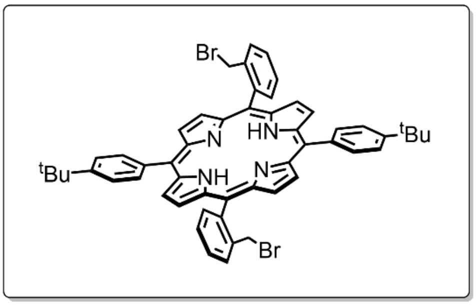

Synthesis following general procedure **A**. Porphyrin **8** (230 mg, 0.28 mmol, 1.0 eq.), DCM (60 mL), HBr (17 mL, 33% in AcOH). The product was obtained as purple solid (230 mg, 0.26 mmol, 94% yield).
^{1}H NMR (500 MHz, RT, $CDCl_3$): δ [ppm] = 8.96 (d, ^3J = 4.8 Hz, 4H, β-pyrrole); 8.73 (d, ^3J = 4.8 Hz, 4H, β-pyrrole); 8.23 – 8.18 (m, 4H, *o*-^{t}Bu-aryl-*H*); 8.08 (dd, ^3J = 7.5, 1.4 Hz, 2H, *o*-aryl-*H*); 7.95 (dd, ^3J = 8.0, 1.4 Hz, 2H, *m*-aryl-*H*); 7.84 – 7.79 (m, 6H, *m*-^{t}Bu-aryl-*H*, *p*-aryl-*H*); 7.66 (td, ^3J = 7.5, 1.4 Hz, 2H, *m*-aryl-*H*); 4.34 (s, 4H, Ar-C*H*$_2$-Br); 1.65 (s, 18H, C(C*H*$_3$)$_3$); -2.59 (s, 2H, N-*H*).
^{13}C NMR (126 MHz, RT, $CDCl_3$) δ [ppm] = 150.8 (*p*-^{t}Bu-aryl-*Cq*); 141.7 (*Cq1*-aryl); 139.3 (*o*-aryl-*Cq*); 138.9 (*Cq1*-^{t}Bu-aryl); 134.8 (*o*-^{t}Bu-aryl-*C*); 134.6 (*o*-aryl-*C*); 131.0 (β-pyrrole); 129.9 (*m*-aryl-*C*); 129.4 (*p*-aryl-*C*); 126.7 (*m*-aryl-*C*); 123.8 (*m*-^{t}Bu-aryl-*C*); 120.8 (bridg*Cq*-^{t}Bu-aryl); 116.5 (bridg*Cq*-aryl); 35.0 (*C*(CH$_3$)$_3$); 32.1 (Ar-*C*H$_2$-Br); 31.8 (C(*C*H$_3$)$_3$).

ESI-MS (m/z): [M + H]$^+$ calcd $C_{54}H_{48}Br_2N_4$ for 913.2305, found 913.2334.
UV/Vis (DMSO): λ nm (ε) 421 (205440), 516 (11599), 551 (5979), 592 (4979), 648 (4598).

Synthesis of 11

Synthesis following general procedure **B**. Porphyrin **10** (120 mg 0.13 mmol, 1.0 eq.), 1,4,10,13-tetraoxa-7,16-diazacyclooctadecane (76 mg, 0.29 mmol, 2.2 eq.), $NaHCO_3$ (24 mg, 0.29 mmol, 2.2 eq.) and dry toluol (12 mL). The product was obtained as purple powder (110 mg, 0.09 mmol, 66% yield).

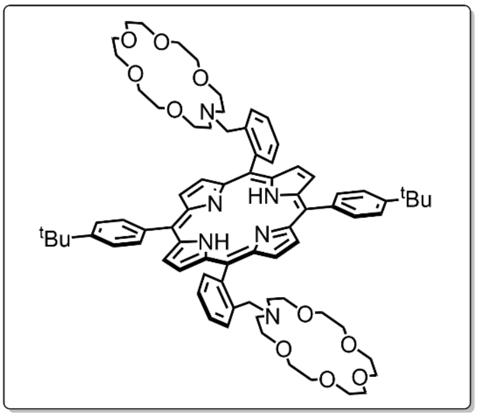

^{1}H NMR (500 MHz, RT, $CDCl_3$): δ [ppm] = 8.84 (d, ^3J = 4.8 Hz, 4H, β-pyrrole); 8.64 (d, ^3J = 4.8 Hz, 4H, β-pyrrole); 8.19 (d, ^3J = 8.0 Hz, 2H, *o*-^{t}Bu-aryl-*H*); 8.10 (t, ^3J = 7.1 Hz, 4H, *o*-^{t}Bu-aryl-*H*, *m*-aryl-*H*); 8.02 (dd, ^3J = 7.6, 1.4 Hz, 2H, *o*-aryl-*H*); 7.80 – 7.73 (m, 6H, *m*-^{t}Bu-aryl-*H*, *p*-aryl-*H*); 7.57 (td, ^3J = 7.5, 1.4 Hz, 2H, *m*-aryl-*H*); 3.38 (d, ^3J = 3.4 Hz, 16H, -O-CH_2-); 3.32 (s, 4H, Ar-CH_2-N), 3.31 – 3.25 (m, 8H; -O-CH_2-); 3.25 – 3.17 (m, 8H; -O-CH_2-); 3.08 (t, ^3J = 5.9 Hz, 8H, NCH_2-CH_2); 2.33 (t, ^3J = 5.9 Hz, 8H, N-CH_2); 1.61 (s, 18H, C(CH_3)$_3$); -2.66 (s, 2H, N-*H*).
^{13}C NMR (126 MHz, RT, $CDCl_3$) δ [ppm] = 150.6 (*p*-^{t}Bu-aryl-*Cq*); 141.8 (*o*-aryl-*Cq*); 141.2 (*Cq1*-aryl); 139.0 (*Cq1*-^{t}Bu-aryl); 134.5 (*o*-^{t}Bu-aryl-*C*); 134.1 (*o*-aryl-*Cq*); 131.4 (β-pyrrole); 128.5 (*p*-aryl-*C*); 128.2 (*m*-aryl-*C*); 124.9 (*m*-aryl-*C*); 123.8 (*m*-^{t}Bu-aryl-*C*); 120.3 (bridg*Cq*-^{t}Bu-aryl); 118.4 (bridg*Cq*-aryl); 70.6 (-O-*C*H_2-); 70.1 (-O-*C*H_2-); 69.6 (NCH_2-*C*H_2); 58.5 (Ar-*C*H_2-N); 53.9 (N-*C*H_2); 35.0 (*C*(CH_3)$_3$); 31.8 (C(*C*H_3)$_3$).

ESI-MS (m/z): $[M + H]^+$ calcd $C_{78}H_{96}N_6O_{10}$ for 1277.7261, found 1277.7251.
UV/Vis (DMSO): λ nm (ε) 424 (161694), 518 (9964), 555 (5789), 595 (4463), 650 (4109).

Synthesis of o-CE2

Synthesis following general procedure **C**. Porphyrin **11** (100 mg, 0.08 mmol, 1.0 eq.) in $CHCl_3$ (20 ml) was mixed with $Co(OAc)_2·4H_2O$ (292 mg, 1.17 mmol, 15 eq.), dissolved in MeOH (5 ml). The product was obtained as purple powder (88 mg, 0.07 mmol, 85 %).

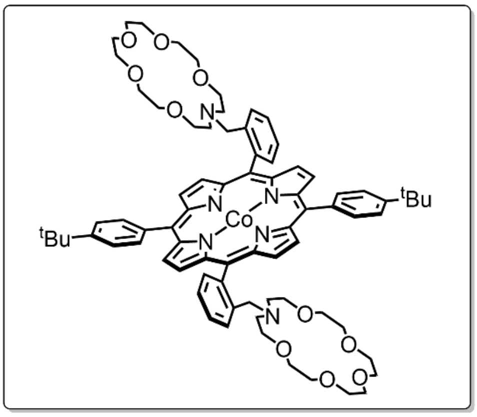

ESI-MS (m/z): $[M + 2K]^{2+}$ calcd $C_{78}H_{94}CoN_6O_{10}$ for 705.7813, found 705.7802.
UV/Vis (DMSO): λ nm (ε) 418 (177824), 535 (13402).

Synthesis of 12

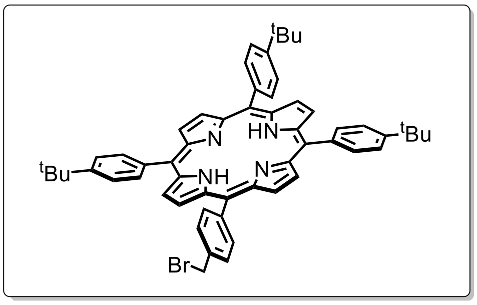

Synthesis following general procedure **A**. Porphyrin **9** (800 mg, 0.97 mmol, 1.0 eq.), DCM (200 mL), HBr (32 mL, 33% in AcOH). The product was obtained as purple solid (750 mg, 0.86 mmol, 97% yield).

^{1}H NMR (500 MHz, RT, $CDCl_3$): δ [ppm] = 8.89 (d, ^3J = 6.1 Hz, 6H, β-pyrrole); 8.66 (d, ^3J = 4.7 Hz, 2H, β-pyrrole) ; 8.23 – 8.10 (m, 6H, *o*-^{t}Bu-aryl-*H*); 8.08 (dd, ^3J = 7.5, 1.4 Hz, 1H, *o*-aryl-*H*); 7.92 (dd, ^3J = 8.1, 1.4 Hz, 1H, *m*-aryl-*H*); 7.83 – 7.79 (m, 1H, *p*-aryl-*H*); 7.80 – 7.73 (m, 6H, *m*-^{t}Bu-aryl-*H*); 4.26 (s, 2H, Ar-C*H*2-Br); 1.62 (d, ^3J = 3.5 Hz, 27H, C(C*H*3)3); -2.69 (s, 2H, N-*H*).
^{13}C NMR (126 MHz, RT, $CDCl_3$) δ [ppm] = 150.7 (*p*-^{t}Bu-aryl-*Cq*); 141.7 (*Cq1*-aryl); 139.4 (*o*-aryl-*Cq*); 139.1 (*Cq1*-^{t}Bu-aryl); 134.8 (*o*-aryl-*C)*; 134.6 (*o*-^{t}Bu-aryl-*C*); 131.4 (β-pyrrol-*C*);129.9 (*m*-aryl-*C*); 129.3 (*p*-aryl-*C)*; 126.7 (*m*-aryl-*C)*; 123.7 (*m*-^{t}Bu-aryl-*C*); 120.9 (bridg*Cq*-^{t}Bu-aryl); 120.5 (bridg*Cq*-^{t}Bu-aryl); 115.8 (bridg*Cq*-aryl); 35.1 (*C*(CH3)3); 32.1 (Ar-*C*H2-Br); 31.8 (C(*C*H3)3).
ESI-MS (m/z): [M + H]$^+$ calcd $C_{57}H_{55}BrN_4$ for 877.3677, found 877.3670.
UV/Vis (DMSO): λ nm (ε) 420 (284122), 516 (11876), 551 (6123), 591 (3994), 646 (4116).

Synthesis of H2(o-CE) (13)

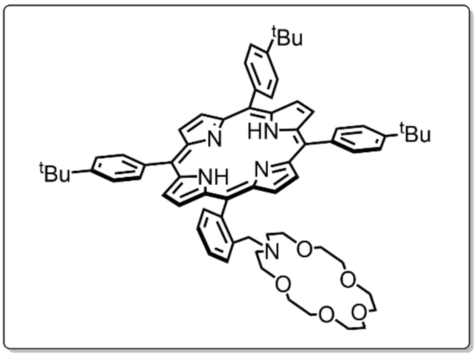

Synthesis following general procedure **B**. Porphyrin **12** (200 mg, 0.23 mmol, 1.0 eq.), 1,4,10,13-tetraoxa-7,16-diazacyclooctadecane (73 mg, 0.28 mmol, 1.2 eq.), $NaHCO_3$ (24 mg, 0.28 mmol, 1.2 eq.) and dry Toluol (25 mL) The product was obtained as purple powder (150 mg, 0.14 mmol, 61 %).

^{1}H NMR (500 MHz, RT, $CDCl_3$): δ [ppm] = 8.93 – 8.88 (m, 6H, β-pyrrole); 8.70 (d, ^3J = 4.8 Hz, 2H, β-pyrrole); 8.27 – 8.19 (m, 3H, *o*-^{t}Bu-aryl-*H*); 8.17 – 8.10 (m, 4H, *o*-^{t}Bu-aryl-*H, o*-aryl-*H*); 8.03 (dd, ^3J = 7.9, 1.4 Hz, 1H, *m*-aryl-*H*); 7.80 (dq, ^3J = 11.8, 6.9 Hz, 7H, *m*-^{t}Bu-aryl-*H, p*-aryl-*H*); 7.63 (td, ^3J = 7.5, 1.4 Hz, 1H, *m*-aryl-*H*); 3.37 (s, 2H, Ar-C*H*2-N); 3.15 – 3.09 (m, 8H, -O-C*H*2-); 3.00 – 2.96 (m, 4H, -O-C*H*2-); 2.93 – 2.89 (m, 4H, -O-C*H*2-); 2.72 (t, *J* = 6.0 Hz, 4H, NCH2-C*H*2); 2.29 (t, ^3J = 6.0 Hz, 4H, N-C*H*2); 1.65 (s, 27H, C(C*H*3)3); -2.62 (s, 2H, N-*H*).
^{13}C NMR (126 MHz, RT, $CDCl_3$) δ [ppm] = 150.7 (*p*-^{t}Bu-aryl-*Cq)*; 141.9 (*o*-aryl-*Cq*); 141.5 (*Cq1*-aryl); 139.4 (*Cq1*-^{t}Bu-aryl); 139.2 (*Cq1*-^{t}Bu-aryl); 134.6 (*o*-^{t}Bu-aryl-*C)*; 134.3 (*o*-aryl-*C*); 131.5 (β-pyrrol-*C*); 128.6 (*m*-aryl-*C*, *p*-aryl-*C*); 125.1 (*m*-aryl-*C*); 123.8 (*m*-^{t}Bu-aryl-*C*) 120.5 (bridg*Cq*-^{t}Bu-aryl); 120.3 (bridg*Cq*-^{t}Bu-aryl); 118.4 (bridg*Cq*-aryl); 70.3 (-O-*C*H2-); 70.1 (-O-*C*H2-); 69.9 (-O-*C*H2-); 69.4 (NCH2-*C*H2); 58.6 (Ar-*C*H2-N); 53.6 (N-*C*H2); 35.1 (*C*(CH3)3); 31.8 (C(*C*H3)3).

ESI-MS (m/z): [M + H]$^+$ calcd $C_{69}H_{79}N_5O_5$ for 1058.6154, found 1058.6176.
UV/Vis (DMSO): λ nm (ε) 423 (233051), 516 (9868), 553 (5482), 593 (2949), 648 (3193).

Synthesis of o-CE

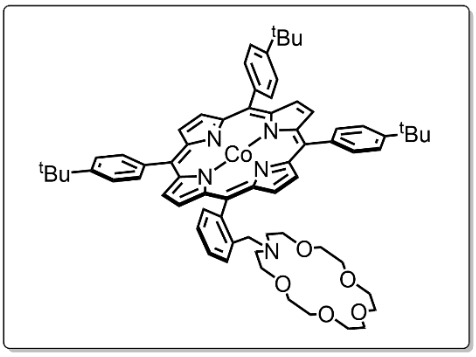

Synthesis following general procedure **C**. Porphyrin **13** (100 mg, 0.09 mmol, 1.0 eq.) in $CHCl_3$ (20 ml) was mixed with $Co(OAc)_2 \cdot 4H_2O$ (292 mg, 1.17 mmol, 15 eq.), dissolved in MeOH (5 ml). The product was obtained as purple powder (90 mg, 0.08 mmol, 85 %).

ESI-MS (m/z): $[M + K]^+$ calcd $C_{69}H_{77}CoN_5O_5$ for 1153.4888, found 1153.4914.
UV/Vis (DMSO): λ nm (e) 418 (227437), 536 (17633).

Homogenous Electrochemistry

Cyclic voltammetry measurements were carried out in a three-electrode configuration using a 5 mL electrochemical cell. A glassy carbon working electrode (3 mm diameter, geometric surface area = 7 mm², AUTOLAB) and a platinum wire counter electrode were employed. Potentials were measured versus an Ag/Ag^+ pseudoreference electrode and subsequently referenced to the Fc/Fc^+ couple in dry DMF at a scan rate of 100 mV s^{-1}. Prior to each measurement, the glassy carbon electrode was polished with 1 µm alumina, rinsed thoroughly with deionised water, and dried with a lint-free tissue. In a typical experiment, a 0.5 mM solution of the catalyst in DMF containing 0.1 M $N(nBu_4)PF_6$ (or KPF_6) as supporting electrolyte was purged with argon or CO_2 before measurement. Controlled potential electrolysis (CPE) using a custom-made electrochemical cell where the counter electrode is separated from electrolyte by a glass frit. Measurements were performed using 0.5 mM catalyst as well as 1M Trifluoroethanol (TFE) as proton source.

Electrode Preparation

To prepare the catalyst coated gas diffussion electrodes as cathodes a catalytic ink was prepared which consisted of 5 mg synthesized cobalt complex,10 mg carbon black (ENSACO 250 G) and 50 µL Sustainion XA-9 solution (5 wt% in ethanol, purchased from dioxide materials) per 10 mL isopropanol used. The catalytic ink was dispersed *via* sonication for 30 min in an ultrasonic bath. Afterwards the ink was constantly stirred at r.t. during the further electrode preparation process. The ink was applied to the gas diffusion layer (Carbon Cloth, W1S1011 purchased from Quintech, electrode area: 2 cm^2) *via* dropcasting at 95 °C in steps of 100 µL until the desired loading (0.5 mg/cm^2 catalyst and 1 mg/cm^2 carbon black) was reached. The electrodes were dried for at least an hour under ambient conditions prior to catalysis. The electrodes which consisted of additional alakali salt were prepared likewise wherein the given equivalent of salt was added to the ink prior to sonication. GDE with different loadings were prepared equally, solely the amount of weight in catalyst was adjusted respectively.
For the preparation of IrO_2 coated titanium felts as anodes a catalytic ink was prepared. The herein given values equal for 21 2 cm^2 circular titanium felts. IrO_2 (120 mg) was dispersed in 2 mL HPLC grade water and 5.8 mL isopropanol. Then Triton X-100 (23.3 µL) was added as surfactant and the mixture was sonicated for 15 min. After sonication 60 µL of a PTFE dispersion (30 wt% in ethanol) was added and the mixture

was stirred at r.t. during the following process. The anodes were spray coated manually at 110 °C using an Iwata Eclipse airbrush until the desired loading (1 mg/cm^2) was reached. The Triton X-100 was burned out at 300 °C for 10 min.

Cyclic Voltammetry of GDEs

To study the electrochemical properties of the as prepared GDEs CV measurements were performed in a standard three electrode set up using a PalmSens4 Potentiostat. The catalyst coated GDEs (area of 0.5 cm^2 in solution) were utilized as working electrode, an Ag/AgCl (sat. KCl solution) electrode as reference electrode and a platinum mesh as counter electrode. The measurements were performed in a degassed 0.1 M $KHCO_3$ solution. Measurements in presence of CO_2 were conducted after purging the solution with CO_2 for 15 min. The number of active sites was determined according to a previously published procedure using the following equation: [5]

$$\Gamma = \frac{i_p}{v} \times \frac{4RT}{n^2F^2S}$$

Wherein Γ is the number of active sites, i_p the peak current at the applied scan rate v, R the universal gas constant, T the temperature, n the number of transferred electrons, F the faraday constant and S the surface area.

Heterogenous electrochemistry

Electrolysis was performed using an in house built ZGE reported previously consisting of two stainless steel endplate, PTFE insulation plates, copper based current collectors and titanium based flow fields (linear for the anode site, serpiternal for the cathode site). The electrode area is 2 cm^2 (circular) and electrodes are held in place using PTFE gaskets of the corresponding thickness.[6] All experiments were performed using a Gamry 1010B potentiostat and Bronkhorst EL-FLOW mass flow controllers to manage the gas flows. All presented electrolysis results show the averaged values of two separate experiments.

For the experiments performed at r.t. the ZGE was equipped with a blank Ni Foam anode (2cm^2, purchased at goodfellow) and a 40 µm thick PiperION membrane (2.5cmx5cm, purchased at Versogen) which was marinated in the corresponding anolyte over night prior to use. The anolyte was cycled with a speed of 20 mL/min using a Gilson minipuls 3 peristaltic pump. A humidifed CO_2 stream of 20 mL/min was diluted with 2 mL/min of N_2 as internal standard. Prior to electrolysis the system was conditioned *via* a stepwise increase of the applied current density starting from -10 mA/cm^2 up to -50 mA/cm^2 in steps of 10 mA/cm^2 for 30 s each. Afterwards electrolysis was started at current densities of 10 mA/cm^2, 25 mA/cm^2, 50 mA/cm^2 and 100 mA/cm^2 for 30 min consecutively. The product gas stream was analyzed every 30 min of electrolysis *via* online gas chromatography (details below).

The experiments at a temperature of 60 °C were conducted in an oven. The anolyte reservoir and the ZGE have been placed inside the oven whereas the humidifier was placed in an external water bath to keep a temperature of 55 °C (equal to 80% relative humidity). The CO_2 flow of 50 mL/min was diluted with 1 mL/min Ar as internal standard. During all experiments an IrO_2 coated Ti felt (loading of 1 mg/cm^2, area of 2 cm^2) was used as anode and the anolyte was cycled at a speed of 20 mL/min using a Gilson minipuls 3 peristaltic pump. A 40 µm thick PiperION anion exchange membrane (2.5 cmx5 cm, purchased from Versogen) was conditioned *via* soaking in the base given in the description of the text or figure captions for at least 24 h.

Conditioning of the electrodes was performed by applying consecutively increasing current densities starting from -20 mA/cm^2 up to -300 mA/cm^2 in steps of 20 mA/cm^2 which were kept for 30 s each, following a current density of -300 mA/cm^2 was applied for 30 min comprising an analysis of the outlet gas stream *via* Online GC at the end. Subsequently the applied current density was increased stepwise (20 mA/cm^2 30 s each) until -500 mA/cm^2 is reached. This current density was kept for 30 min following a product gas analysis *via* online gas chromatography.
The Faradaic Efficiency (FE) of gaseous products was calculated using the following equation:

$$FE(\%)=\frac{znF}{Q}\times 100\%$$

Herein z is the number of transferred electrons during the process, n the amount of substance, F the faraday constant and Q the total charge passed.

Gas Chromatography

For electrolysis experiments at r.t. the outlet gas stream of the ZGE was directly connected to an Agilent Technologies 7820A gas chromatograph equipped with two columns (HP-Plot Q and a HP-Molsieve 5 Å column for product separation) and a flame ionization detector (FID), a thermal conductivity detector (TCD) and a Ni-based methanizer. Herein Argon was used as carrier gas. The sample flow towards the column was regulated by a Bronkhorst EL-FLOW mass flow controller (1.67 mL/min). GC Samples of the cell outlet gas stream were analyzed every 30 min and the resulting chromatograms were evaluated using the software UniChrom V. Detection of the following compounds was possible using the FID/TCD: H_2, N_2, CO, CH_4, C_2H_4 and C_2H_6.
Online gas chromatography for the electrocatalysis performed at 60°C was done using a Shimadzu Nexis GC-2030 which is equipped with two columns (a SH-I-1MS column and a Carboxen 1010 Plot column) followed by a particle trap in front of the Barrier Ion Discharge (BID) detector. Helium was used as carrier gas. Detection of the following compounds was possible *via* GC-BID: H_2, Ar, CO, CH_4, C_2H_4 and C_2H_6.

UV/vis Spectroscopy of redissolved Complexes

UV/vis spectra of the redissolved complexes from the GDE surface were recorded on a Shimadzu UV-1900i. The samples were prepared by placing a cut off piece of the GDE in 2 mL of DMSO and sonicating the samples for 10 min. After sonication the suspension was filtered over celite to remove the carbon black. UV/vis spectra of the filtrate were recorded.

UV/vis/NIR spectroelectrochemistry

UV/vis/NIR spectroelectrochemical measurements were done under nitrogen atmosphere in a MBRAUN Labstar in a quartz cuvette with Pt honeycomb electrode (PINE research) and a Pt wire pseudo-reference electrode. To probe the sample the spectrophotometer Avaspec-ULS2048CI-EVO-RS with the AvaLight-DH-S-BAL light source was used. The potentials were applied through the potentiostat Metrohm Autolab PGSSTAT204 in the chronoamperometry method.

Scanning Electron Microscopy

Scanning electron microscopy (SEM) was either performed on ZEISS Gemini 2 Merlin equipped with an OXFORD AZtecEnergy X-ray microanalysis system for energy dispersive X-ray spectroscopy (EDX). SEM images were recorded with an acceleration voltage of 20.1 or 21 kV. GDE samples have been washed carefully with HPLC grade water prior to analysis to remove anolyte and carbonate salts.

X-Ray Photoelectron Spectroscopy

The X-ray photoelectron spectra of the electrodes used in this work were acquired using a Nexsa G2 Surface Analysis System (ThermoFischer) with monochromated and microfocused Al Ka-rays. The detector was 128-channel together with a 180°, double-focusing, hemispherical analyzer. For all samples a Shirely-type background and a Lorentzian lineshape were applied. Every spectrum was calibrated against adventitious carbon (set to 284.8 eV). Peaks that were not assigned as satellite peaks were limited to a full width at half maximum of 2.0 eV. GDE samples have been washed carefully with HPLC grade water prior to analysis to remove anolyte and carbonate salts.

X-Ray Computer tomography

X-Ray Computer Tomography (CT) was performed with a Bruker Skyscan 2214 CMOS edition scanner equipped with a Hamamatsu L10711 tungsten source. Electrode samples of roughly 2 mm width were cut and clamped in a custom sample holder for scanning. X-ray images were recorded at 55 kV and 90 µA with an image pixel size of 0.91 µm. A flat field correction was conducted before each scan including an automatic adjustment of the exposure time to values of 846 – 939 ms. Each scan acquired 1801 frames and averaged 4 of them for each position of a full rotation in 0.2° steps. With these settings, a scanning duration of about 3 h, 40 min was reached. A region of interest from the middle of each sample was selected for 3D reconstruction and subsequent analysis using Bruker software.

Additional Figures

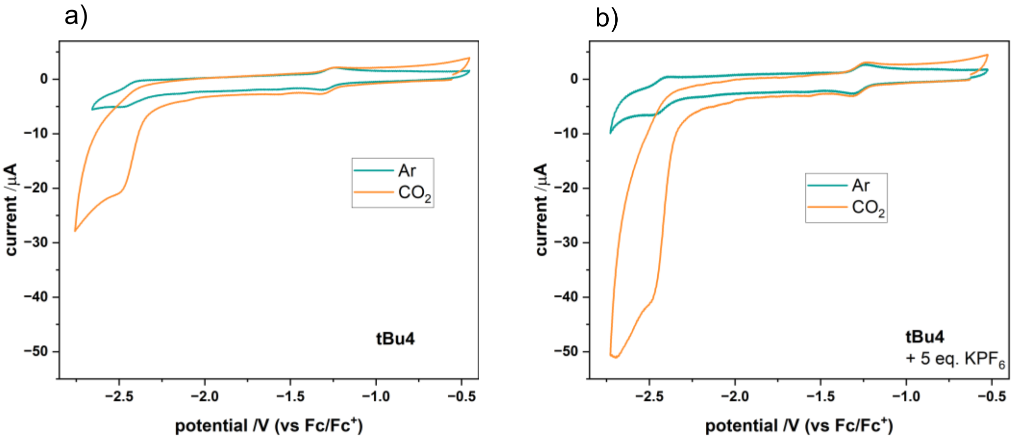

Figure S1. Cyclic voltammograms of **tBu4** recorded under argon (green) and carbon dioxide (orange) using NBu_4PF_6 as the supporting electrolyte (a), and with the addition of 5 equivalents of KPF_6 relative to the catalyst concentration (b).

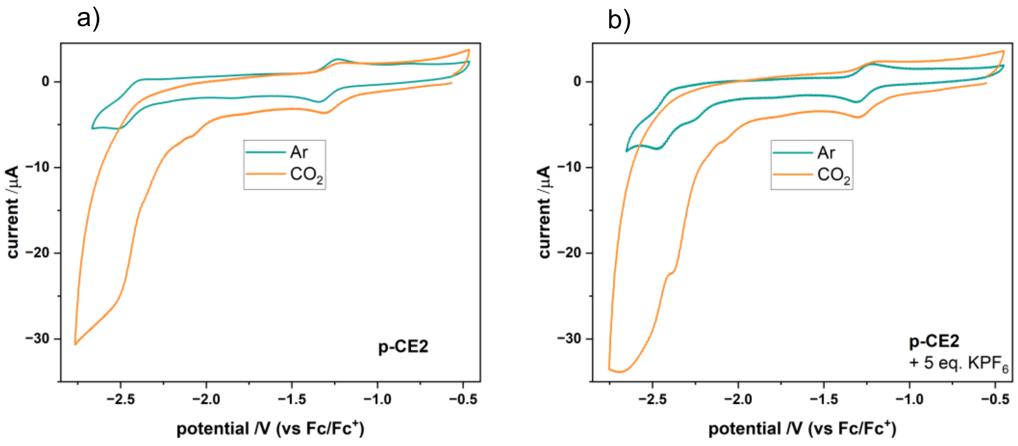

Figure S2. Cyclic voltammograms of **p-CE** recorded under argon (green) and carbon dioxide (orange) using NBu_4PF_6 as the supporting electrolyte (a), and with the addition of 5 equivalents of KPF_6 relative to the catalyst concentration (b).

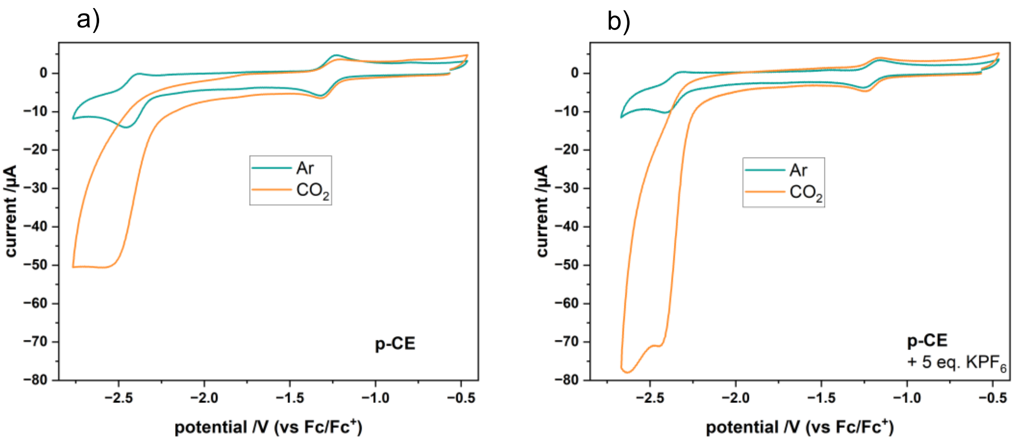

Figure S3. Cyclic voltammograms of **p-CE2** recorded under argon (green) and carbon dioxide (orange) using NBu_4PF_6 as the supporting electrolyte (a), and with the addition of 5 equivalents of KPF_6 relative to the catalyst concentration (b).

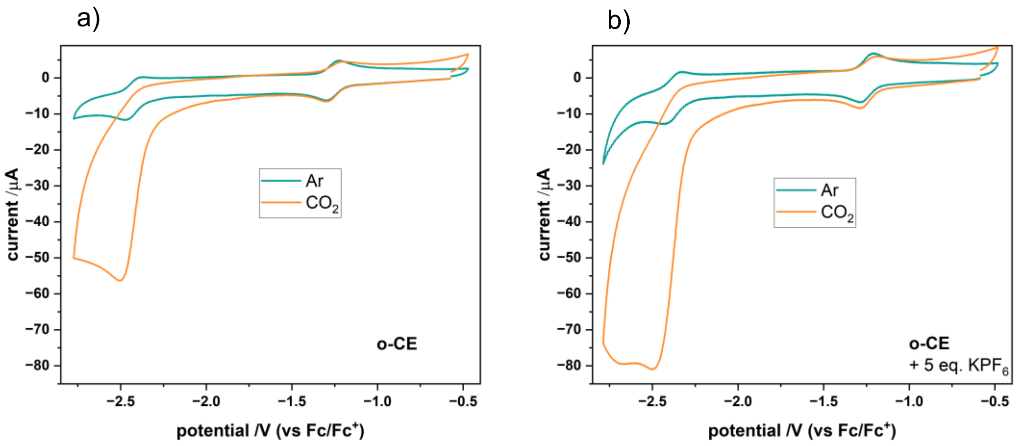

Figure S4. Cyclic voltammograms of **o-CE** recorded under argon (green) and carbon dioxide (orange) using NBu_4PF_6 as the supporting electrolyte (a), and with the addition of 5 equivalents of KPF_6 relative to the catalyst concentration (b).

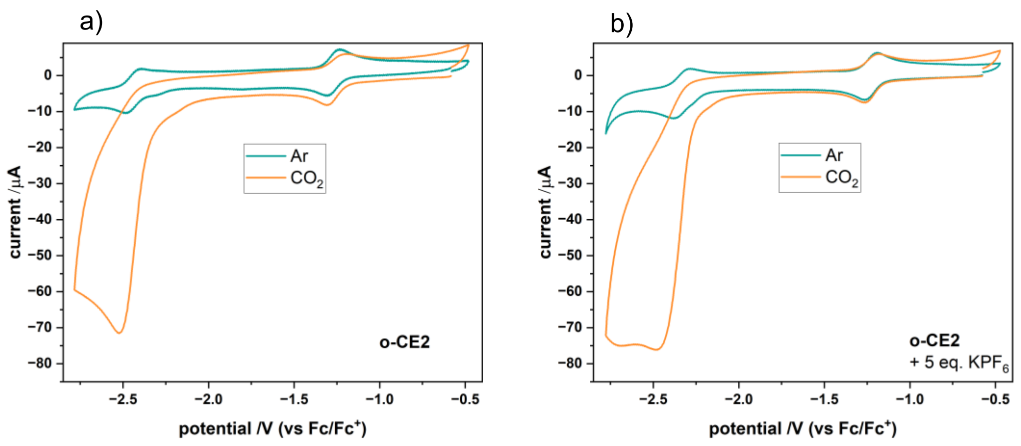

Figure S5. Cyclic voltammograms of **o-CE2** recorded under argon (green) and carbon dioxide (orange) using NBu_4PF_6 as the supporting electrolyte (a), and with the addition of 5 equivalents of KPF_6 relative to the catalyst concentration (b).

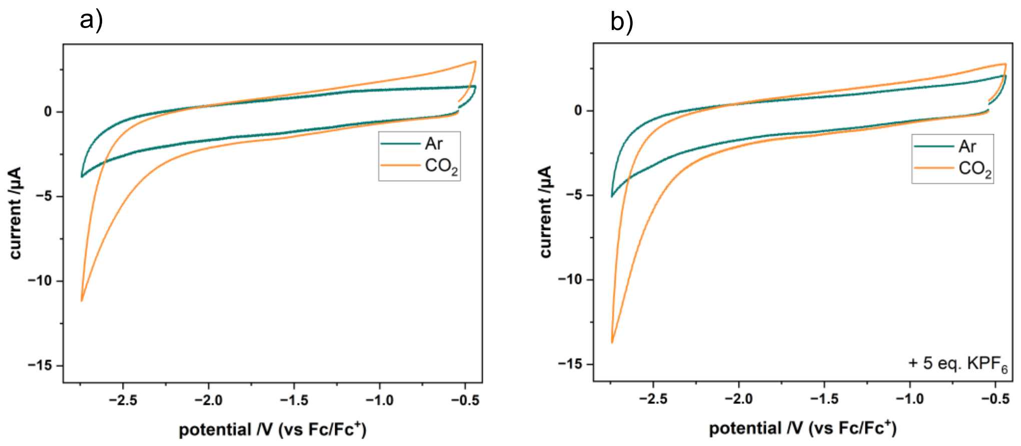

Figure S6. Cyclic voltammograms of the blank electrolyte solution recorded under argon (green) and carbon dioxide (orange) using NBu_4PF_6 as the supporting electrolyte (a), and with the addition of 5 percent of KPF_6 (b).

For comparison with the zero-gap electrolysis, which is the main focus of this work, we evaluated the general performance of selected complexes under homogeneous conditions by conducting controlled potential electrolysis (CPE) supported by pre- and post-electrolysis CV analysis.

Our CPE measurements reveal that both **p-CE2** and **o-CE2** are nearly inactive for homogeneous CO_2RR. After 30 minutes of electrolysis at −2.55 V, only trace amounts of CO and H_2 were detected, in stark contrast to their pronounced activity under heterogeneous conditions (see related sections in the main text).

For **p-CE2**, the overall Faradaic efficiency (FE) is only 15%, split into FE_{CO} = 8% and FE_{H_2} = 7%. The remaining ~85% of the charge is attributed to catalyst deactivation and the initial Co(II)/Co(I) conversion. Accordingly, the catalytic metrics are negligible (TON_{CO} = 0.005, TON_{H_2} = 0.023; TOF_{CO} = 0.009 h^{-1}, TOF_{H_2} = 0.047 h^{-1}), in line with the very low total charge passed (0.02 C). Post-electrolysis CV measurements show complete loss of the cobalt redox features (Figure S7a), which reappear only after polishing the working electrode. This behavior indicates electrode passivation by deposition of inactive material rather than reversible molecular catalysis.

A similar trend is observed for **o-CE2**, which displays a slightly higher total FE of 32% (FE_{CO} = 5%, FE_{H_2} = 27%) and a higher total charge passed (0.03 C). Nevertheless, its overall activity remains very low compared to heterogeneous operation (see related sections in the main text). The selectivity is shifted toward hydrogen evolution (TON_{CO} = 0.003, TON_{H_2} = 0.006; TOF_{CO} = 0.007 h^{-1}, TOF_{H_2} = 0.006 h^{-1}), likely due to the closer proximity of the crown-ether (CE) units to the cobalt center, which facilitates proton shuttling from the liquid phase and thus enhances competition from HER. As with **p-CE2**, catalyst deposition and deactivation are evident (Figure S7b).

In contrast, the **tBu4**-substituted cobalt porphyrin exhibits higher homogeneous activity and stability. CPE experiments show a total charge of 0.05 C with FE_{CO} = 85% and FE_{H_2} = 1%, corresponding to TON_{CO} = 0.101, TON_{H_2} = 0.001 and TOF_{CO} = 0.201 h^{-1}, TOF_{H_2} = 0.003 h^{-1}. Importantly, CV features remain unchanged after electrolysis (Figure S8a), indicating durability of the active molecular species in solution.

The critical role of secondary-sphere interactions is further demonstrated by CPE experiments in the presence of 5 equivalents of KPF_6. For **o-CE2**, potassium binding to the crown ether suppresses efficient proton shuttling and thereby reduces HER competition. As a result, the total charge passed increases substantially to 0.12 C, with FE_{CO} = 76% and FE_{H_2} = 3%. Both activity and selectivity improve markedly (TON_{CO} = 0.229, TON_{H_2} = 0.008; TOF_{CO} = 0.458 h^{-1}, TOF_{H_2} = 0.016 h^{-1}). Notably, no deactivation is observed (Figure S8b); instead, a gradual increase in current density during electrolysis is detected, accompanied by preserved reversibility of the Co(II)/Co(I) redox couple (Figures S9a and S8b). This behavior may indicate deposition of an active material on the electrode surface whose catalytic properties are enhanced by potassium chelation, an observation that correlates well with the behavior seen in heterogeneous zero-gap electrolysis systems.

In contrast, the performance of **tBu4** deteriorates upon addition of KPF_6 (Figures S9b). The total charge decreases to 0.02 C, FE_{CO} drops to 53% (FE_{H_2} = 7%), and the catalytic metrics decline significantly (TON_{CO} = 0.027, TON_{H_2} = 0.003; TOF_{CO} = 0.055 h^{-1}, TOF_{H_2} = 0.007 h^{-1}).

Taken together, these additional homogeneous measurements underscore the importance of matching catalyst structure with the appropriate electrochemical environment. While crown-ether-functionalized cobalt porphyrins are poorly suited for homogeneous CO_2RR due to deactivation and proton-shuttling effects, their secondary-sphere interactions, particularly potassium chelation, become highly advantageous under heterogeneous conditions (see related sections in the main text). This synergy is fully realized in the zero-gap electrolyzer architecture, where immobilization and cation-mediated interactions enable a complete reversal of catalytic performance compared to homogeneous operation.

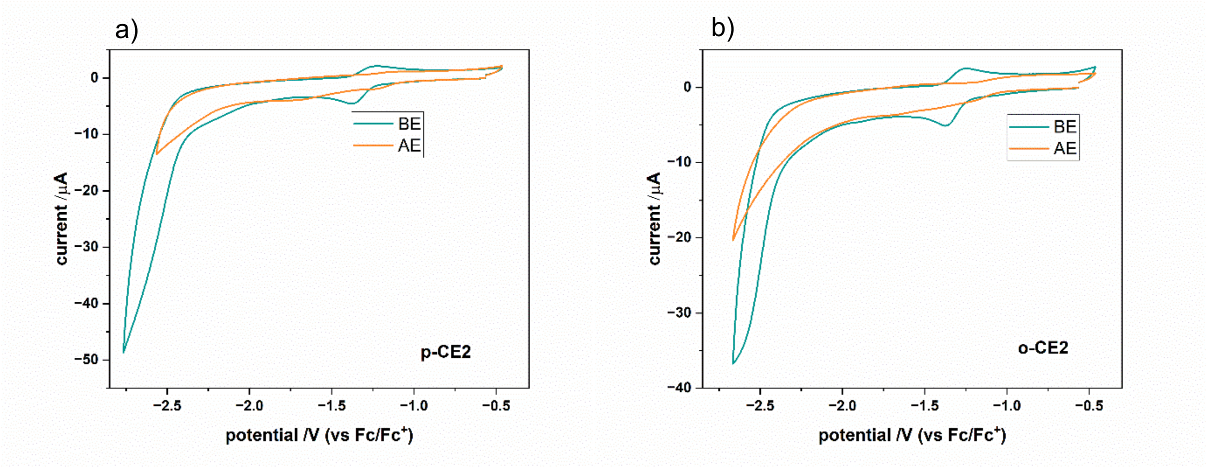

Figure S7. CVs before (green) and after (orange) homogeneous CPE for a) **p-CE2** and b) **o-CE2** under CO_2-atmosphere and 1M TFE as proton source.

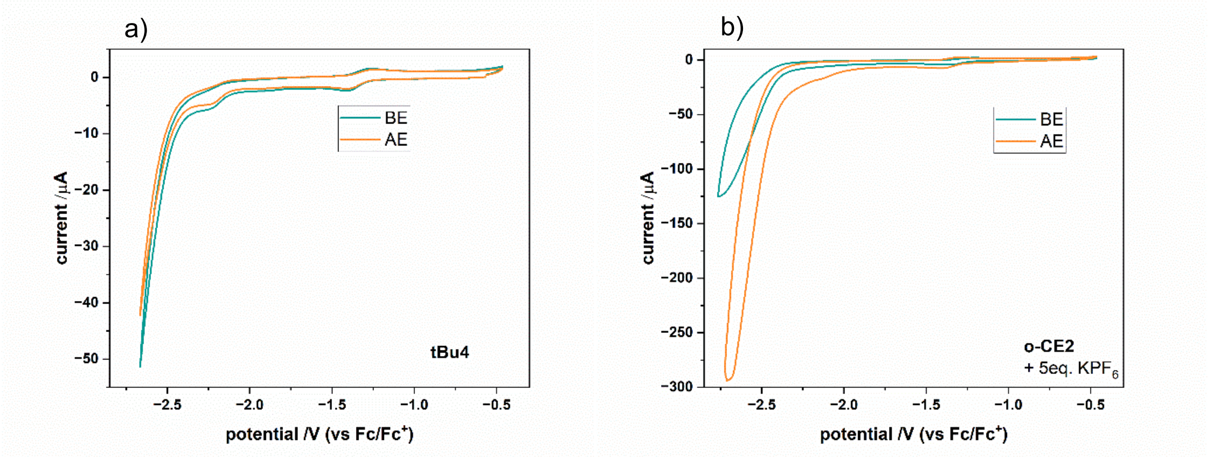

Figure S8. CVs (green) and after (orange) homogeneous CPE for a) **tBu4** and b) **o-CE2** with 5eq. of KPF_6 added under CO_2-atmosphere and 1M TFE as proton source**.**

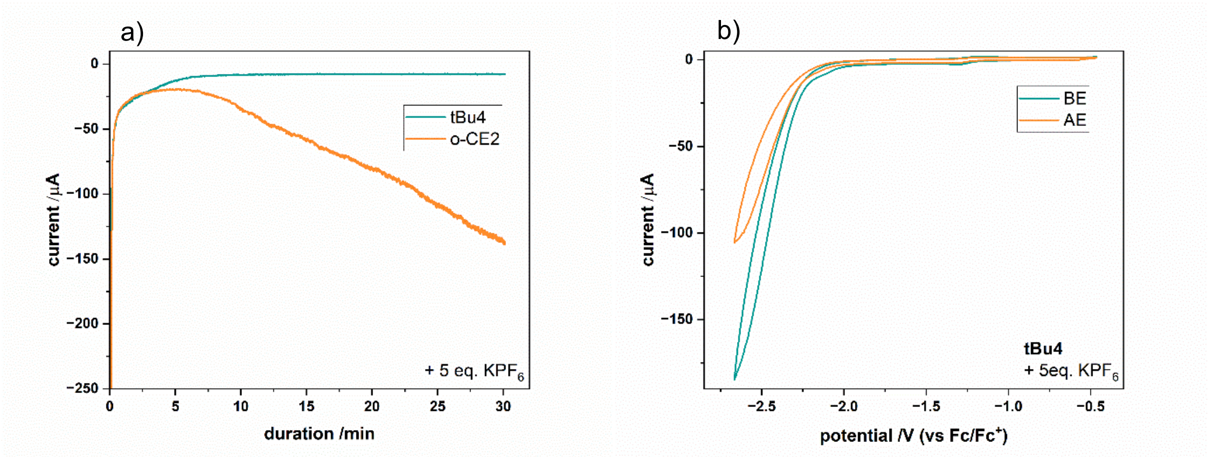

Figure S9. a) CPE curves at -2.55V (vs. Fc/Fc^+) of **o-CE2** (orange) and **tBu4** (green) with 5eq. of KPF_6 added under CO_2-atmosphere and 1M TFE as proton source and b) CVs before (green) and after (orange) homogeneous CPE for **tBu4** with 5eq. of KPF_6 added.

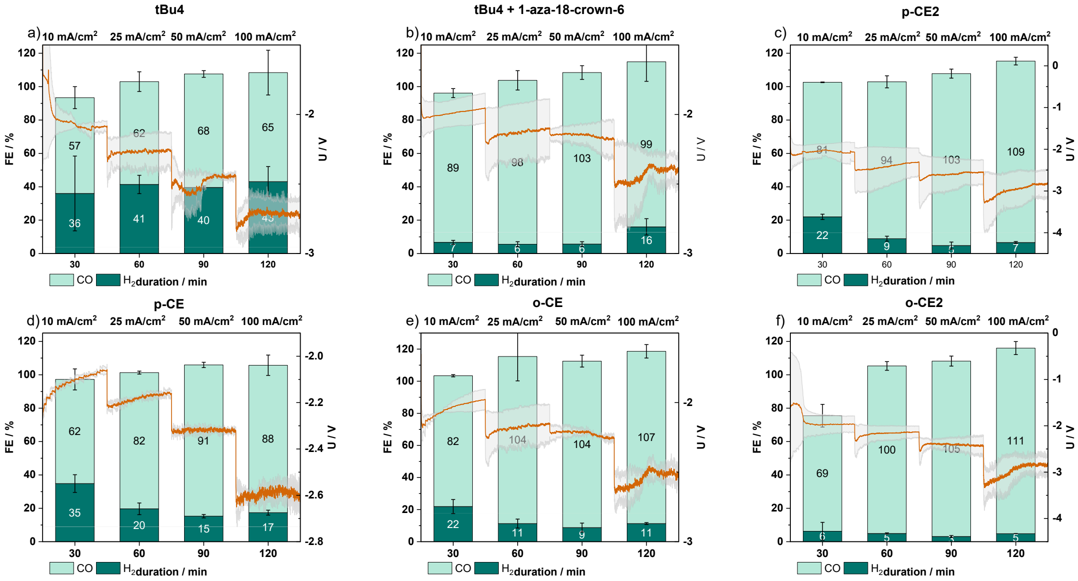

Figure S10. Detailed overview of the achieved FE_{CO} (dark green) and FE_{H2} (light green) pictured as bar chart with the corresponding cell voltage (orange line) achieved at r.t. catalysis for GDE coated with the respective catalyst: a) **tBu4**; b) **tBu4** in presence of 2 eq. 1-aza-18-crown-6; c) **p-CE2**; d) **p-CE**; e) **o-CE**; f) **o-CE2**.

Following the determination of active sites of the GDEs is presented. For all complexes a much lower value than the theoretical values (187 nmol/cm^2 for **tBu4**, 448 nmol/cm^2 for mono-CE complexes and 558 nmol/cm^2 for bis-CE complexes) are found. Strikingly it was found that the number of active sites is not proportional to the catalytic activity observed in ZGE catalysis. Nevertheless, a trend towards a higher number of active sites for the bis-CE complexes is observed. However, the **tBu4** complex also comprises a high number of active sites, while exhibiting the lowest eCO_2R performance. This indicates that the number of active sites is not as decisive as the local environment and intrinsic catalyst activity. Yet, it needs to be considered that the environment present in these CV measurements differs strongly from those in a ZGE. Unfortunately, it is not possible to conduct reliable CV measurements in a ZGE and concomitantly give a more precise determination of active sites. The hydrophobic properties of a GDE might render some catalyst centers embedded within the three-dimensional catalyst layer untouched by the electrolyte and thus "invisible" within these experiments. Thus, these values should rather be considered as trend of active centers than absolute values as a complex multilayered structure is active as cathode and not only the surface of it. Measurements in hydrophobic, non-aqueous solvents were not performed to prevent catalyst leaching into the electrolyte.

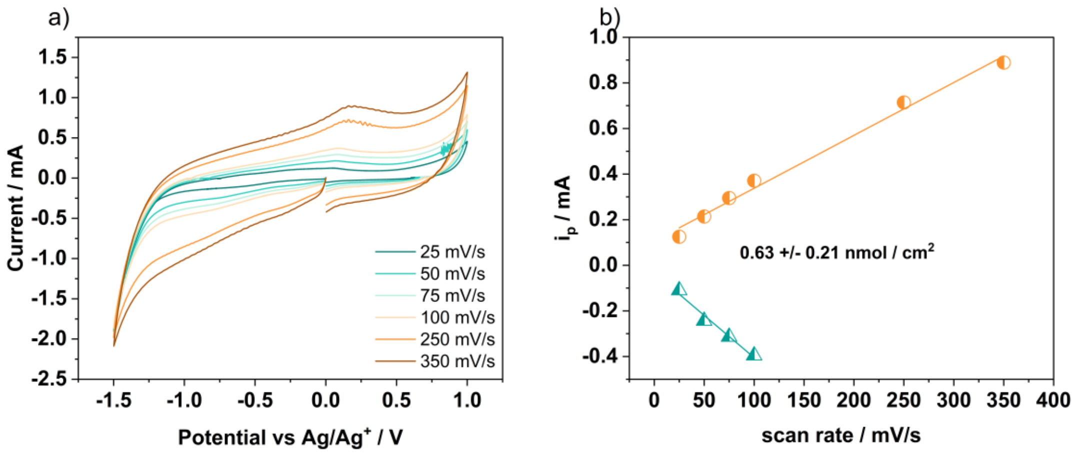

Figure S11. a) CVs of **tBu4@GDE** in 0.1 M $KHCO_3$ under an Ar atmosphere at the given scan rates; b) Plot of the peak currents i_p against the applied scan rate with a trendline that was used to calculate the number of active sites, which is displayed in the graph.

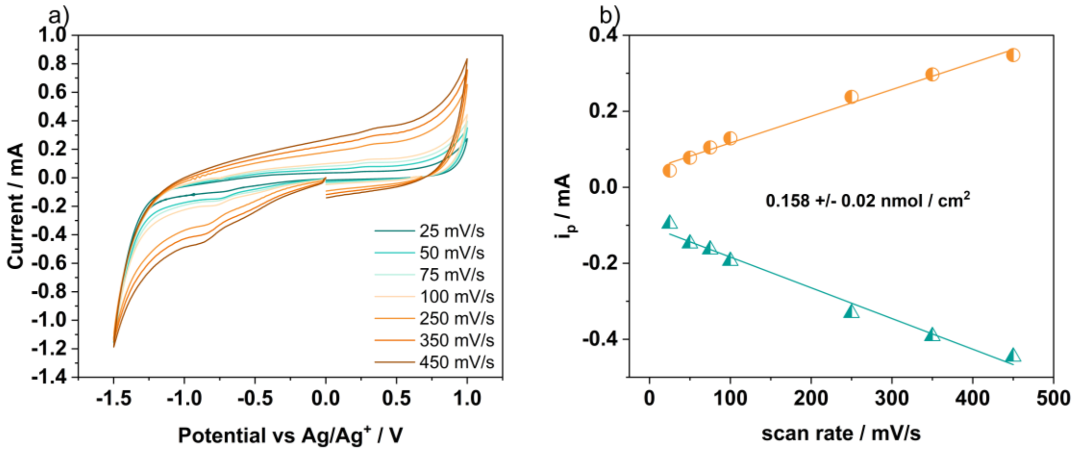

Figure S12. a) CVs of **p-CE@GDE** in 0.1 M $KHCO_3$ under an Ar atmosphere at the given scan rates; b) Plot of the peak currents i_p against the applied scan rate with a trendline that was used to calculate the number of active sites, which is displayed in the graph.

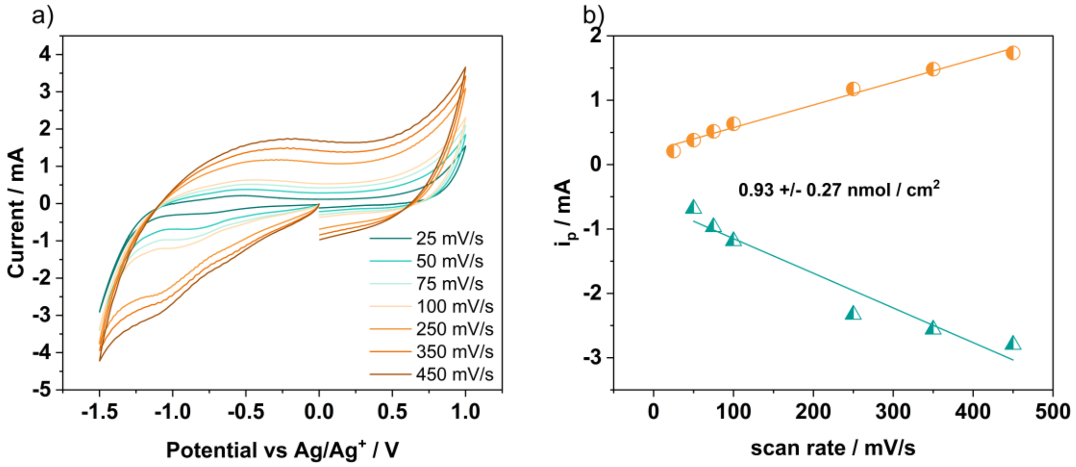

Figure S13. a) CVs of **p-CE2@GDE** in 0.1 M $KHCO_3$ under an Ar atmosphere at the given scan rates; b) Plot of the peak currents i_p against the applied scan rate with a trendline that was used to calculate the number of active sites, which is displayed in the graph.

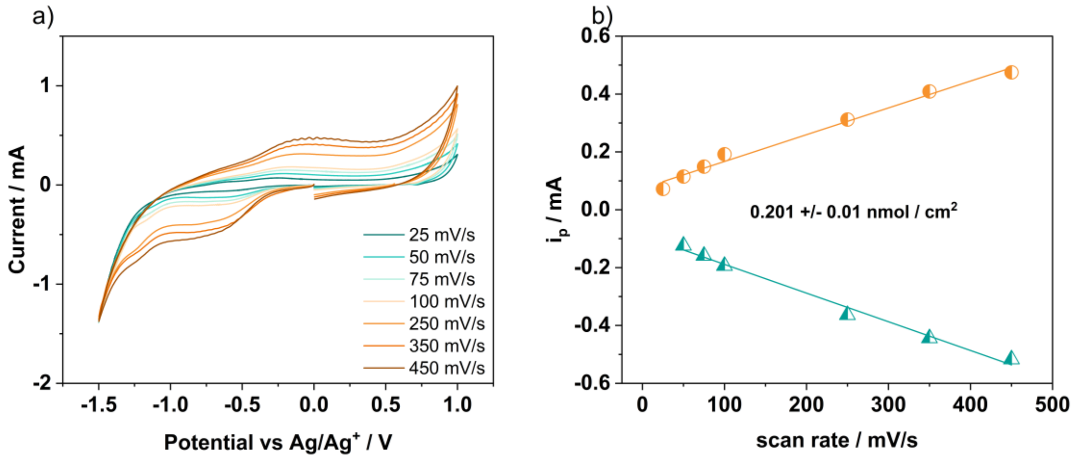

Figure S14.: a) CVs of **o-CE@GDE** in 0.1 M $KHCO_3$ under an Ar atmosphere at the given scan rates; b) Plot of the peak currents i_p against the applied scan rate with a trendline that was used to calculate the number of active sites, which is displayed in the graph.

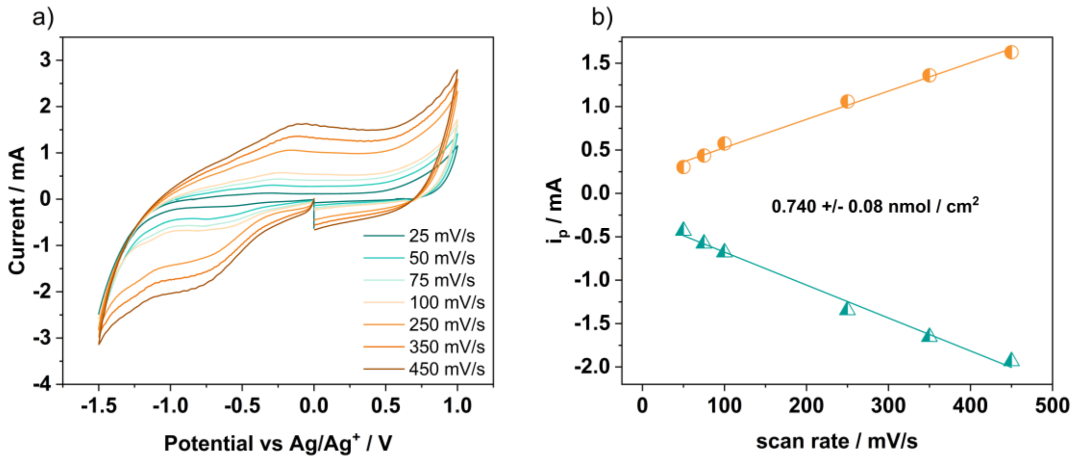

Figure S15.: a) CVs of **o-CE2@GDE** in 0.1 M $KHCO_3$ under an Ar atmosphere at the given scan rates; b) Plot of the peak currents i_p against the applied scan rate with a trendline that was used to calculate the number of active sites, which is displayed in the graph.

The following Figure S16 displays the CVs of a) **p-CE**, b) **p-CE2**, c) **o-CE**, d) **o-CE2** and e) **tBu4@GDE** in 0.1 M $KHCO_3$ under an Ar (green line) or CO_2 (orange line) at a scan rate of 100 mV/s. For all complexes a shift to more positive reduction values of the cobalt center is observed in presence of CO_2. Notably, the trend of the observed reduction potentials is similar to the cell voltages observed in ZGE catalysis: the lower the reduction potential of the GDE, the lower is the cell voltage in ZGE catalysis.

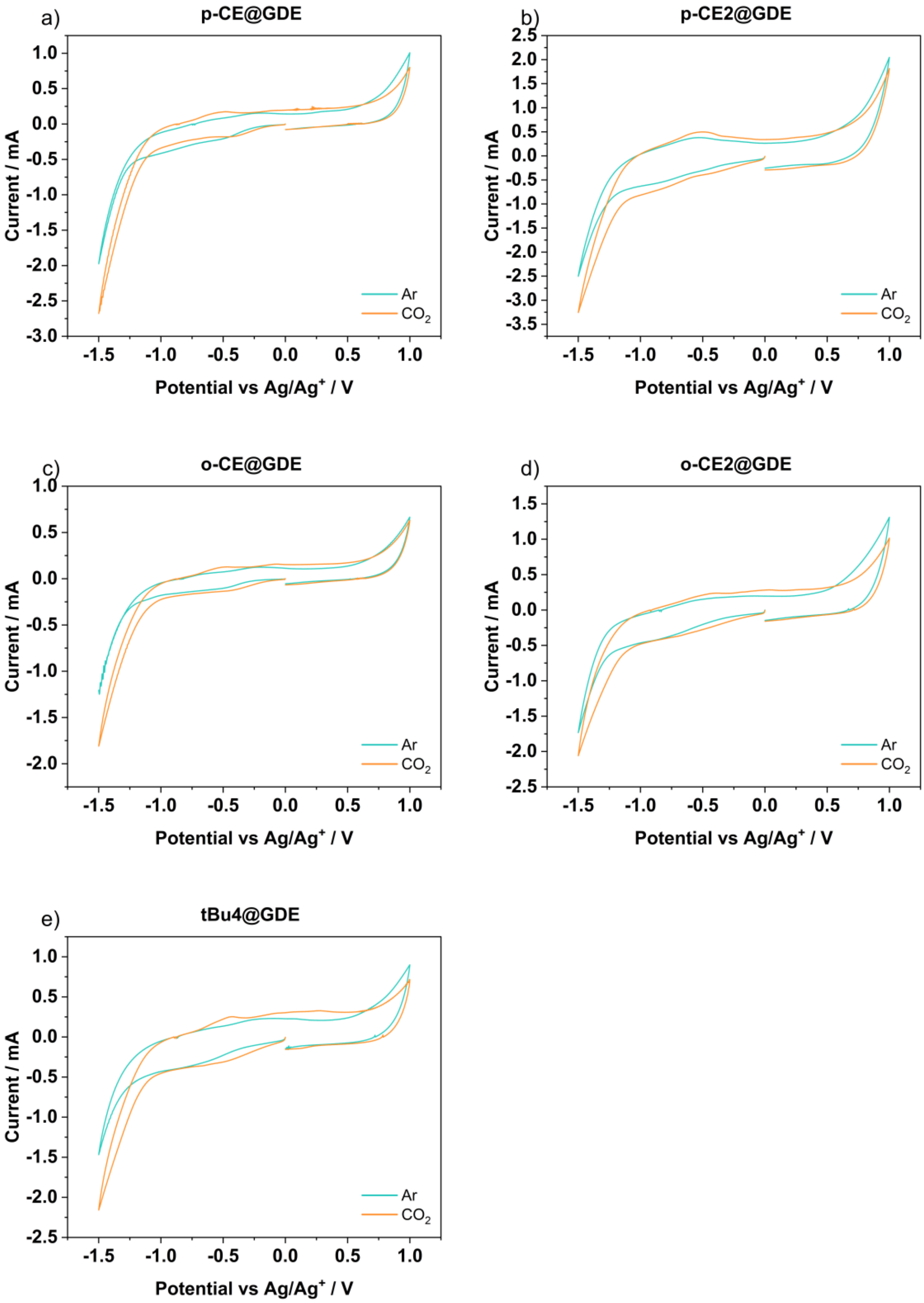

Figure S16.: CVs of a) **p-CE,** b) **p-CE2,** c) **o-CE,** d) **o-CE2** and e) **tBu4@GDE** in 0.1 M $KHCO_3$ under an Ar (green line) or CO_2 (orange line) at a scan rate of 100 mV/s.

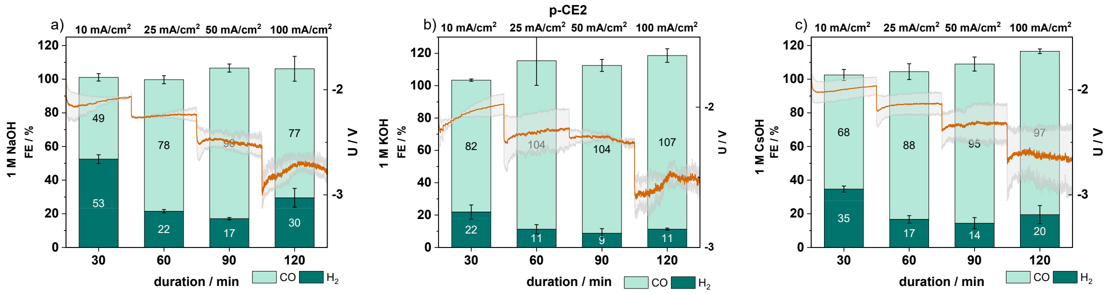

Figure S17. Detailed overview of the achieved FE_{CO} (dark green) and FE_{H2} (light green) pictured as bar chart with the corresponding cell voltage (orange line) achieved at r.t. catalysis for GDE coated with **p-CE2** using the given anolyte: a) 1 M NaOH; b) 1 M KOH; c) 1 M CsOH.

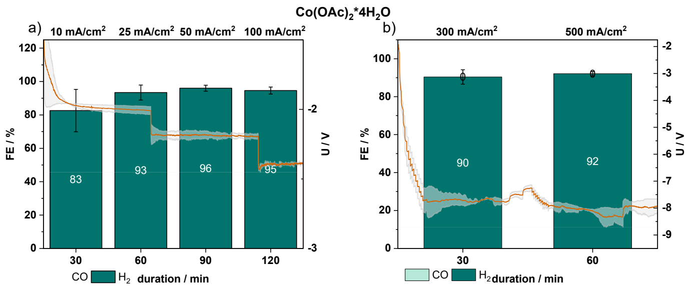

Figure S18. Detailed overview of the achieved FE_{CO} (dark green) and FE_{H2} (light green) pictured as bar chart with the corresponding cell voltage (orange line) achieved with GDEs coated with $Co(OAc)_2*4H_2O$ (equivalent Co loading as for **p-CE2** electrodes) at a) r.t. electrolysis and b) 60°C with 0.1 M CsOH as anolyte.

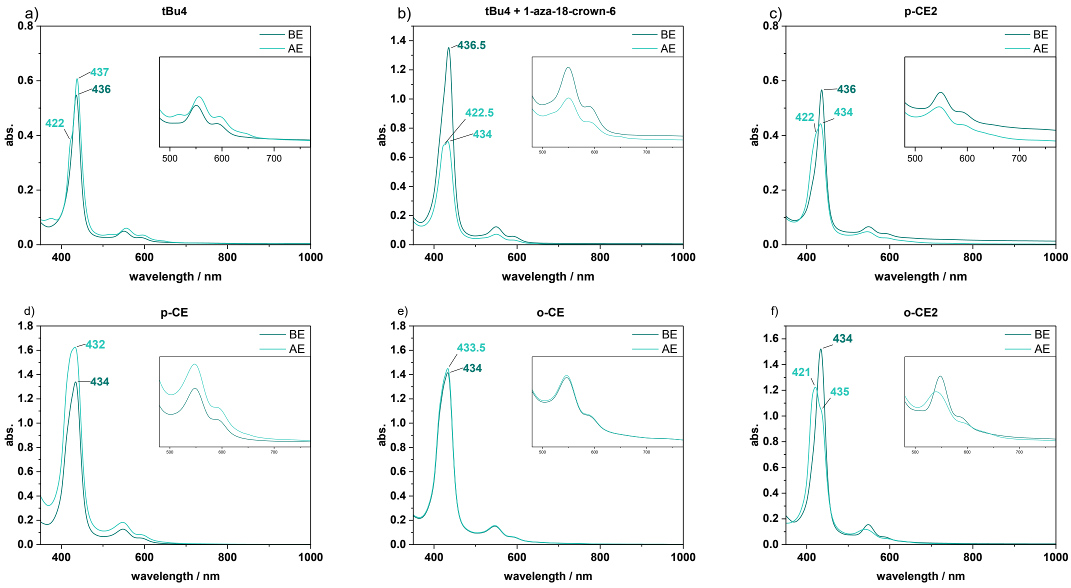

Figure S19. UV/vis spectra of redissolved catalysts which have been extracted from GDEs (before (BE) or after (AE) electrolysis) in DMSO. a) **tBu4**; b) **tBu4** with 2 eq. 1-aza-18-crown-6; c) **p-CE2; d**) **p-CE2**; e) **o-CE**; f) **o-CE2**.

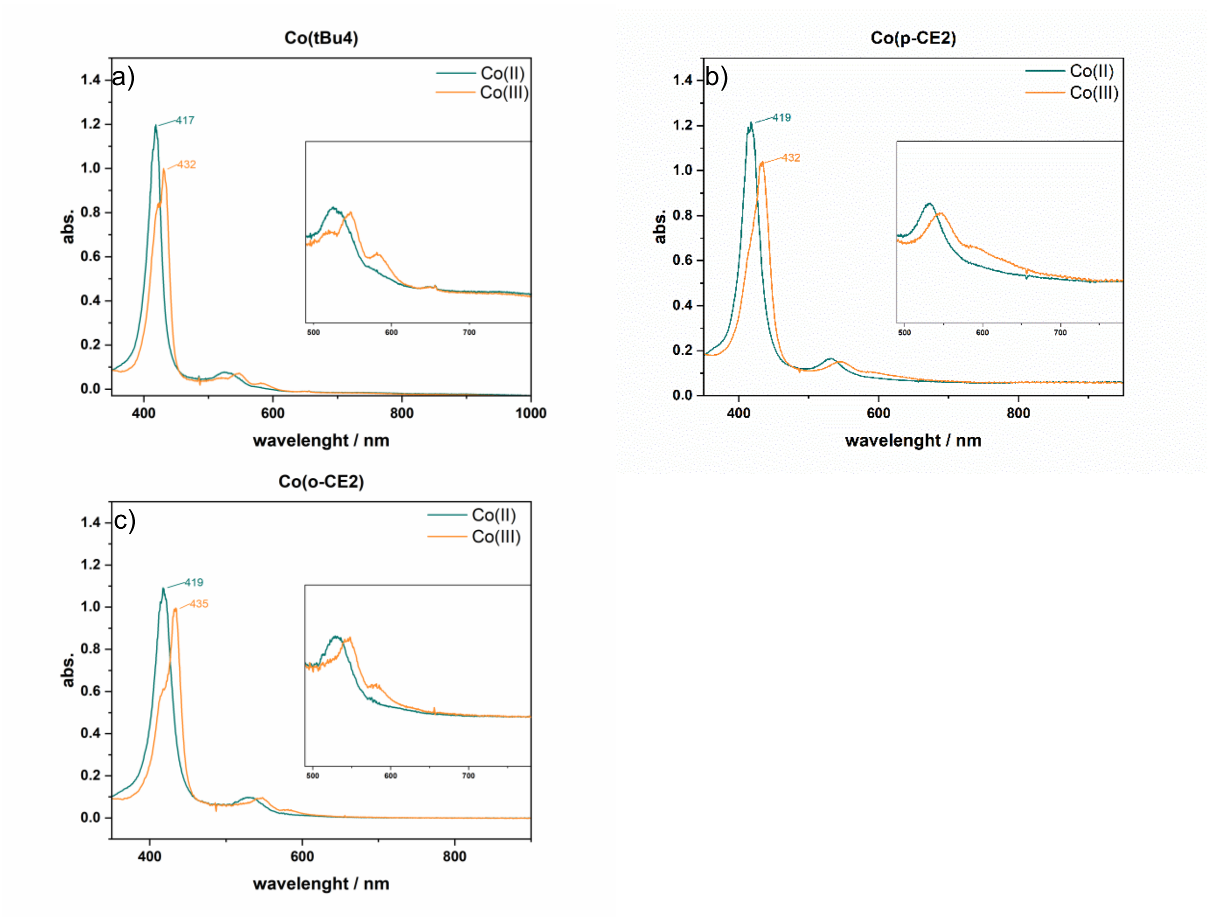

Figure S20. UV/vis/NIR reference spectra obtained in spectroelelectrochemistry experiment with 50µM a) **tBu4**, b) **p-CE2** and c) **o-CE2** in 0.2M $TBAPF_6$ dry DMF under N_2. Co(II) recorded without applied potential and Co(III) recorded at V = 1 V.

As observed in UV/vis spectra shown in Figure S110 the complex is oxidized to a Co(III) species during the preparation process of the electrodes. Thus, it needed to be determined why and when this oxidation process happened. Exemplary following investigations have been performed using the **tBu4** and **p-CE2** complex. As solvents iPrOH, which is used for the ink preparation process, as well as DMSO, which is used for extraction, have been chosen. Firstly the stability of the complexes dissolved in the given solvents has been studied for over 24 h. (Figure S12 and S13) **tBu4** immediately shows a mixture of Co(II) and Co(III) in solution, however after 24 h the more reduced species in predominant in both solvents. In terms of **the p-CE2** the complex oxidizes over the time span of 24 h completely to the Co(III) species in DMSO, wherein the Co(II) complex stays stable in iPrOH. To out rule the possibility of a oxidation induced by other chemicals present in the catalytic Ink UV/vis spectra in presence of the alkaline Binder SustainION XA-9 (abbr. XA-9) were recorded over a time period of 84 h (Figure S14). In case of **p-CE2** in iPrOH the first 24h have been monitored by measurements every 15 min(Figure S17). Herein no oxidation of the complex is visible, thus the observed oxidation of the complex after 84 h has to happen after more than 24 h. The **tBu4** seems to be more prone to oxidation in presence of XA-9 since it shows majorly the Co(III) after 2h od testing. In DMSO the reduced Co(II) species seems to be preferred for both complexes in presence of XA-9. Hence, the observed Co(III) on the GDE surface is unlikely to be induced by the XA-9. In addition, the stability of the complexes in presence of a non-alkaline binder, here Nafion, was also investigated (Figure S15). In presence of Nafion **p-CE2** rapidly oxidizes to its Co(III) form in case of

both tested solvents. In contrast the Co(II) species of **tBu4** is more stable in presence of Nafion when dissolved in iPrOH, in DMSO however it oxidizes as well. Thus XA-9 was kept as binder for following experiments since it is less likely to interact with the complexes. To rule out that the oxidation in presence of XA-9 is promoted by the sonication process UV/vis spectra have been recorded of a **p-CE2-**XA-9 solution after sonication for 30 min as well as of a filtered catalytic ink (Figure S16a). In both spectra no oxidation is observed. Hence, the oxidation needs to take place either during the drop casting process or over the storage time. Therefore, the complex was extracted from a freshly prepared GDE and a UV/vis spectrum was recorded which clearly shows the Co(III) complex. (Figure S16b) Thus, the oxidation of the Co(II) to the Co(III) complex is happening during the actual process of drop casting either induced by the time span or heat.

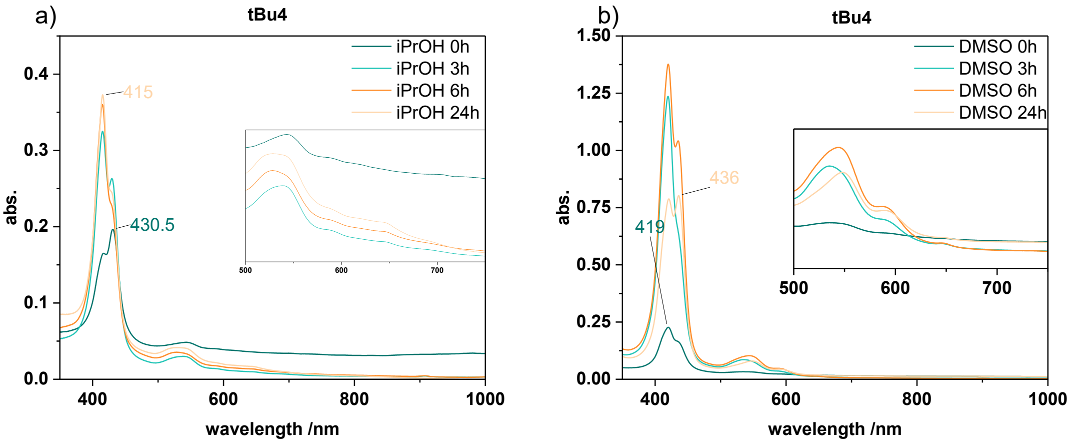

Figure S21. UV/vis spectra recorded of **tBu4** during a 24 h stability test in a) iPrOH and b) DMSO.

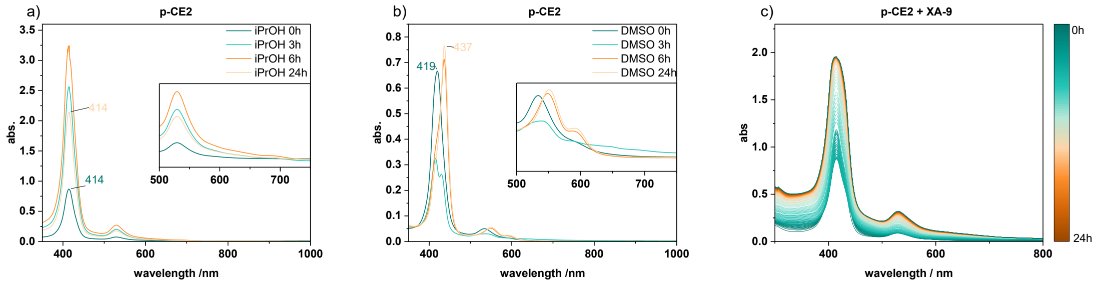

Figure S22. UV/vis spectra recorded of **p-CE2** during a 24 h stability test in a) iPrOH, b) DMSO and c) in presence of XA-9 with a spectrum recorded every 15 min over 24 h.

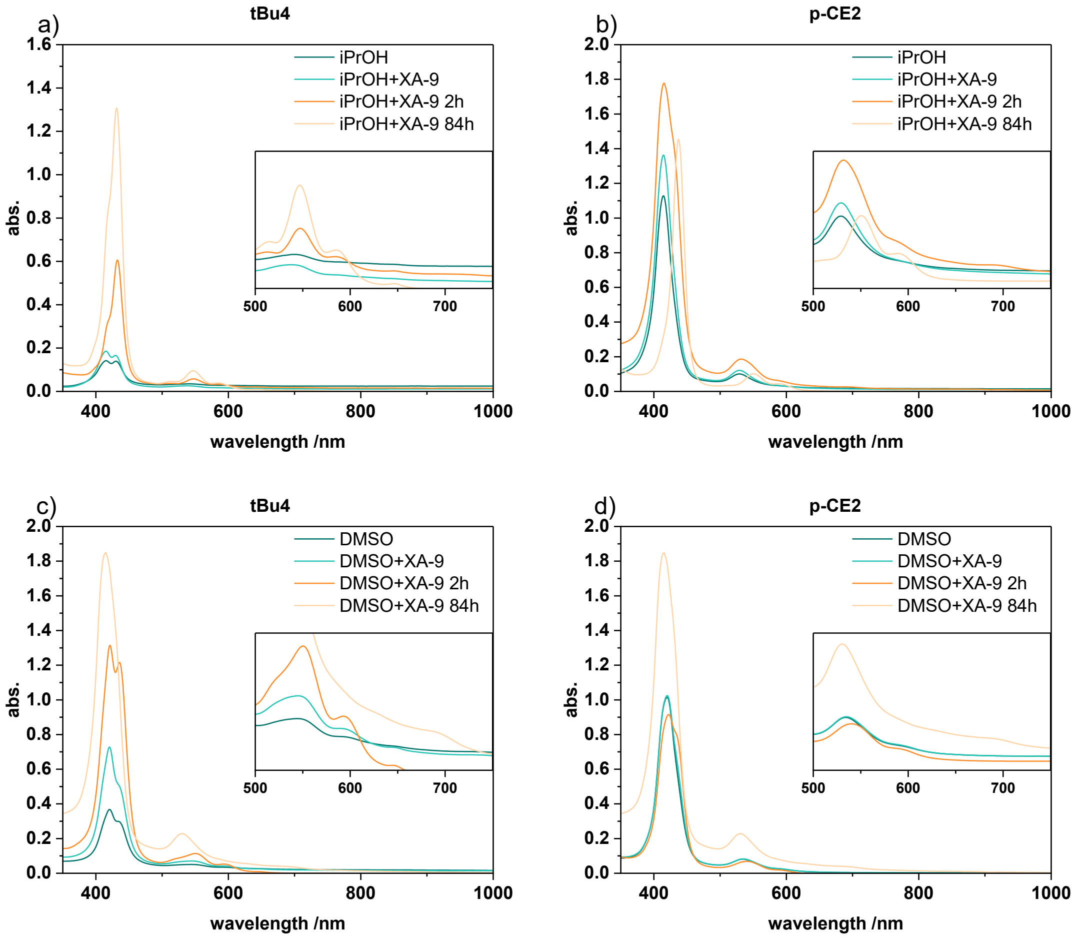

Figure S23. UV/vis spectra of **tBu4** (a and c) and **p-CE2** (b and d) during a 84 h stability test in presence of XA-9 in iPrOH(a and b) or DMSO(c and d).

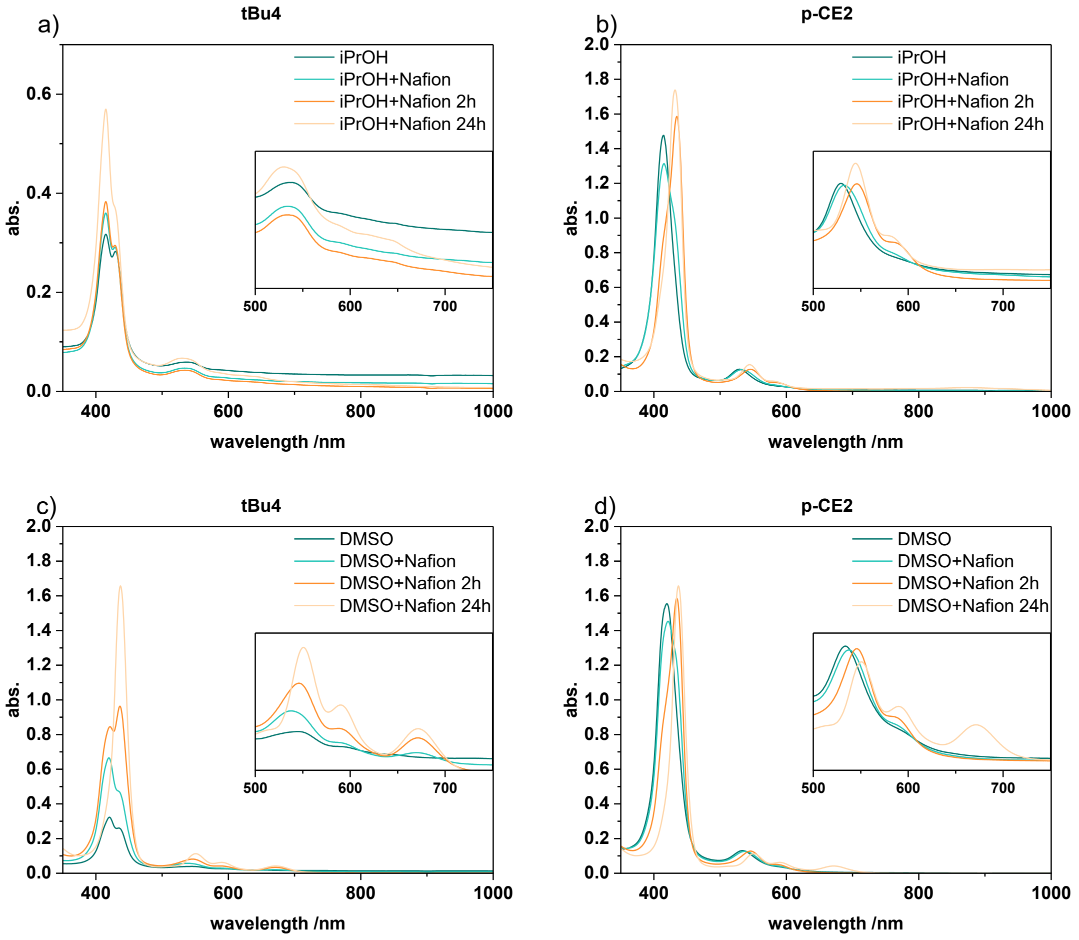

Figure S24. UV/vis spectra of **tBu4** (a and c) and **p-CE2** (b and d) during a 24 h stability test in presence of Nafion in iPrOH(a and b) or DMSO(c and d).

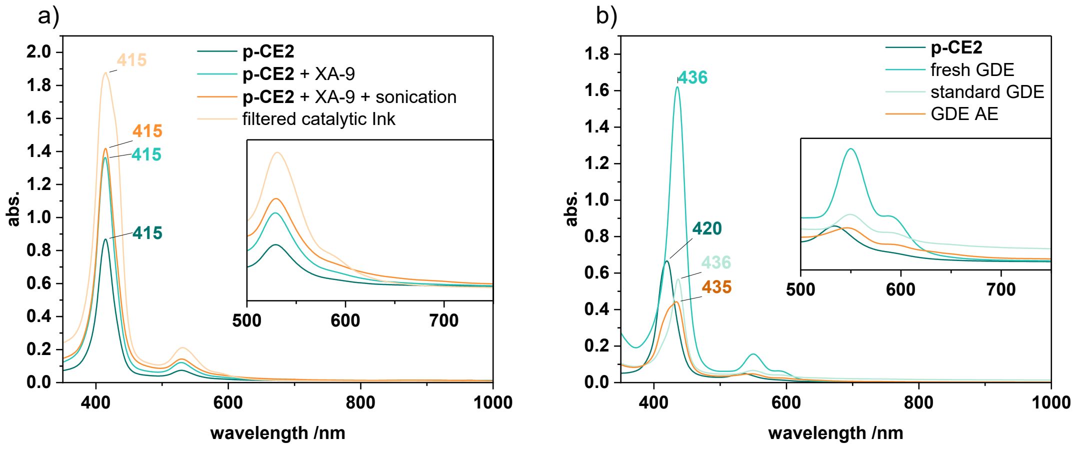

Figure S25. a) Comparison of the UV/vis spectra of a fresh **p-CE2** solution, a complex solution with XA-9 added, a sonicated solution of the complex and XA-9 and a filtered catalytic Ink; b) Comparison of the UV/vis spectra of a fresh **p-CE2** solution, the extracted species of a freshly prepared GDE, a standard GDE and an GDE AE.

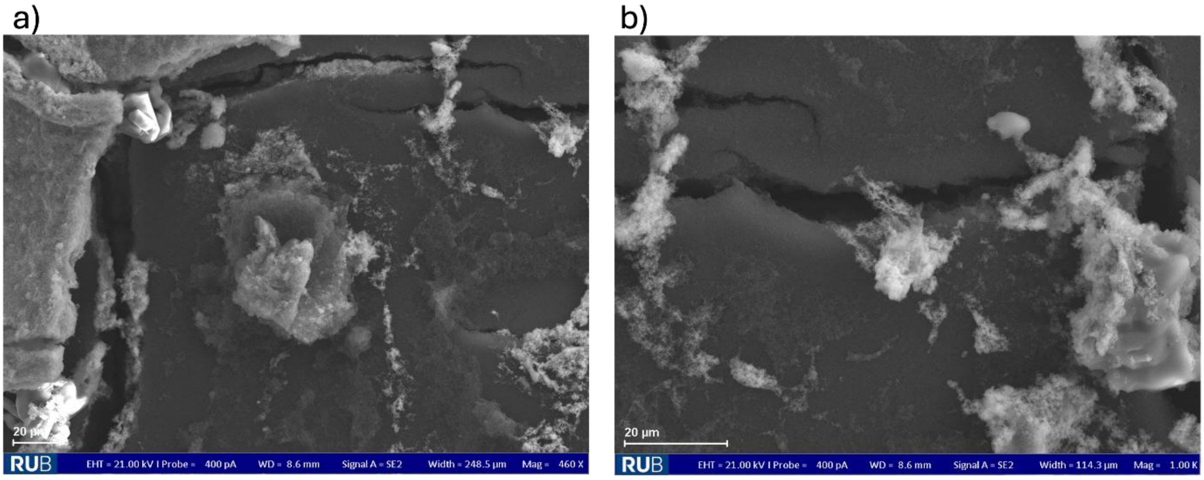

Figure S26. SEM images of a **p-CE2** coated GDE after electrolysis performed at r.t. which has been sonicated in DMSO for further UV/vis analysis at magnifications of a) 460x and b) 1000x.

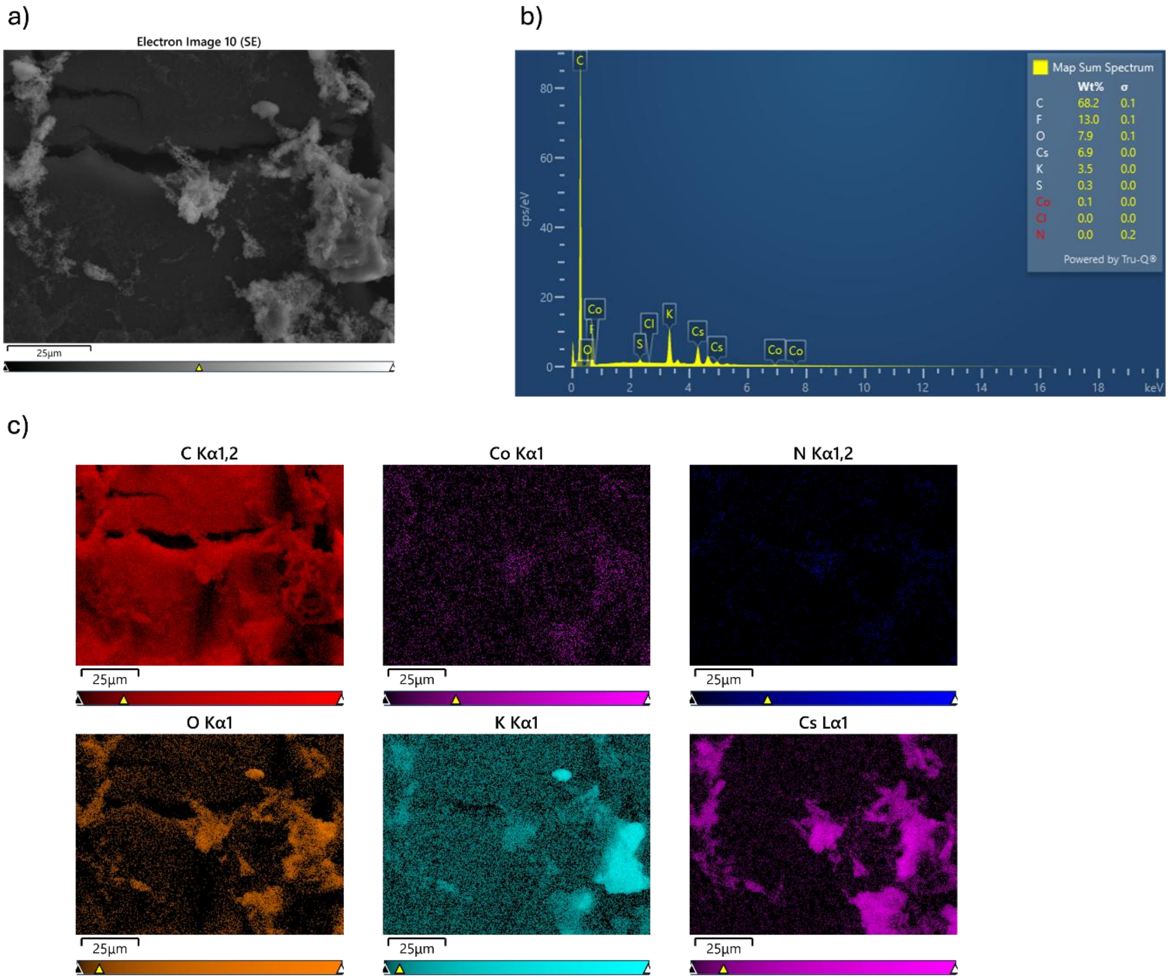

Figure S27. a) SEM image of a **p-CE2** coated GDE after electrolysis performed at r.t. which has been sonicated in DMSO for further UV/vis analysis; b) EDX spectrum of the sample; c) elemental mapping of the sample.

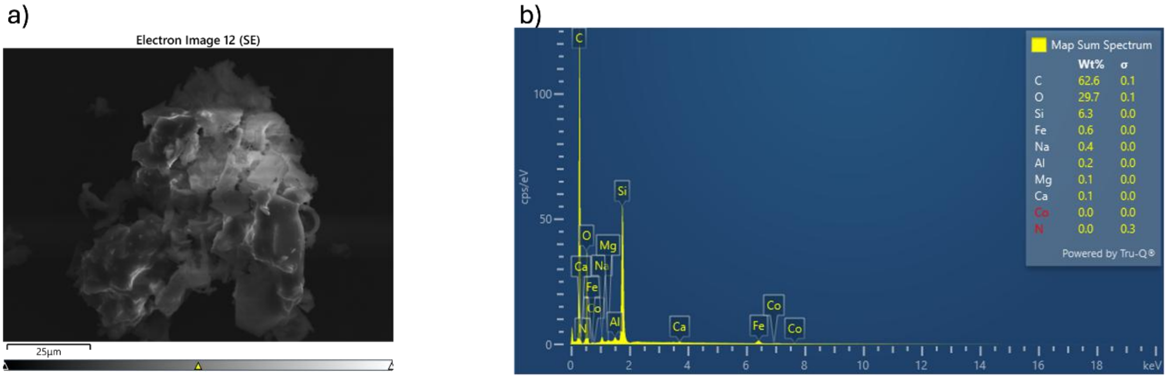

Figure S28. a) SEM image of celite used for filtration of the suspension resulting from sonication in DMSO of a **p-CE2** coated GDE after electrolysis performed at r.t.; b) EDX spectrum of the sample.

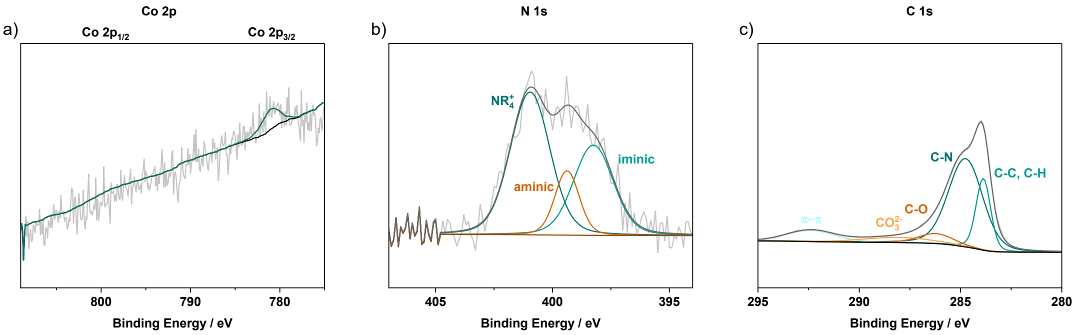

Figure S29. Measured XPS spectra of a GDE coated with **Co(pCE2)** after electrolysis at r.t. and subsequent sonication in DMSO for UV/vis/NIR analysis of the redissolved complex A) Co 2p orbital; B) N 1s orbital; C) C 1s orbital.

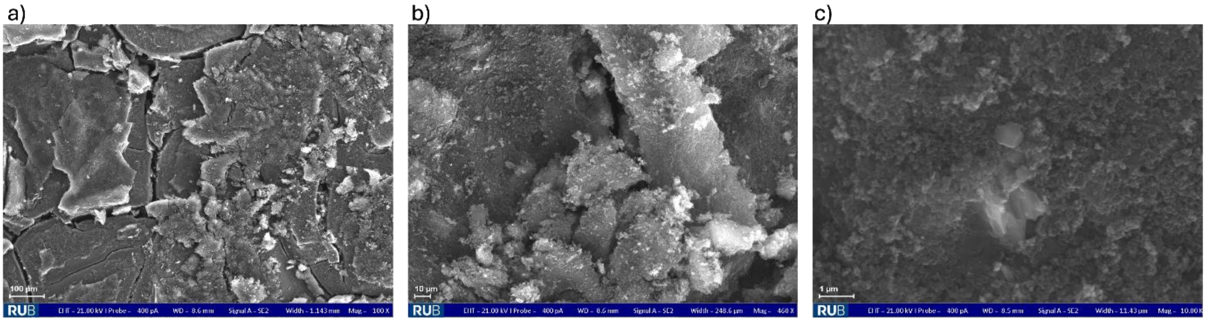

Figure S30. SEM images of a pristine **p-CE2** coated GDE at magnifications of a) 100x; b) 460x and c) 10000x.

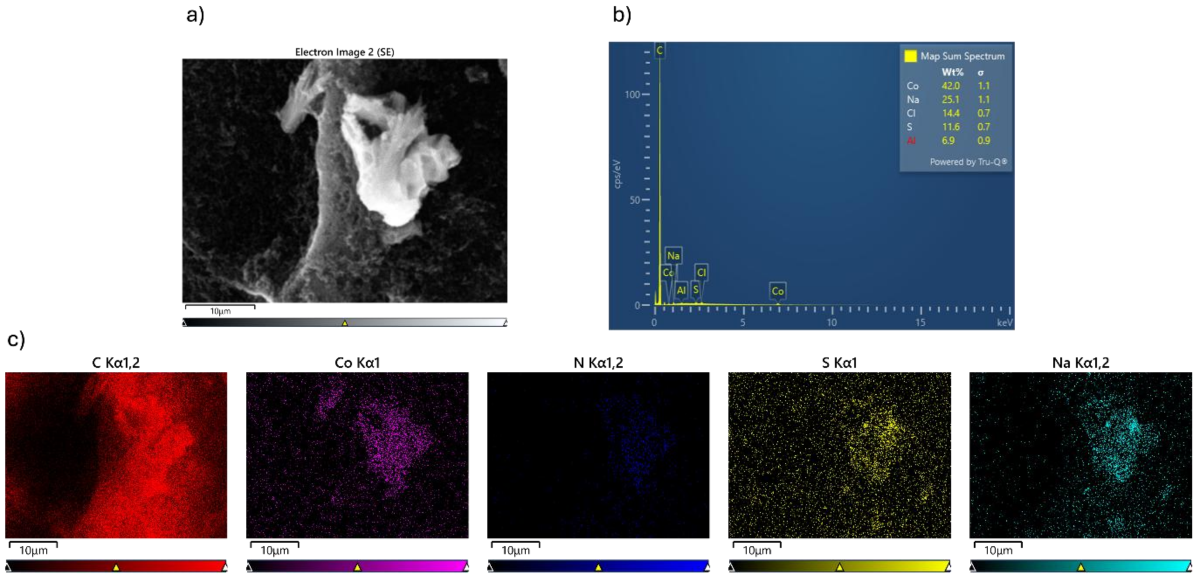

Figure S31. a) SEM image of a pristine **p-CE2** GDE; b) EDX spectrum of the sample; c) elemental mapping of the sample.

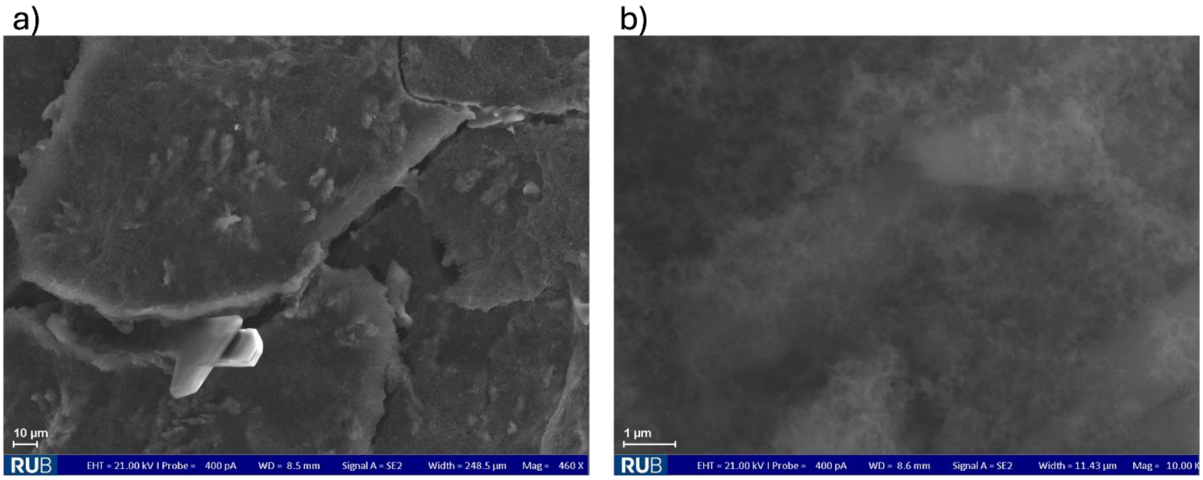

Figure S32. SEM images of a **p-CE2** coated GDE after electrolysis performed at r.t. at magnifications of a) 460x and b) 10000x.

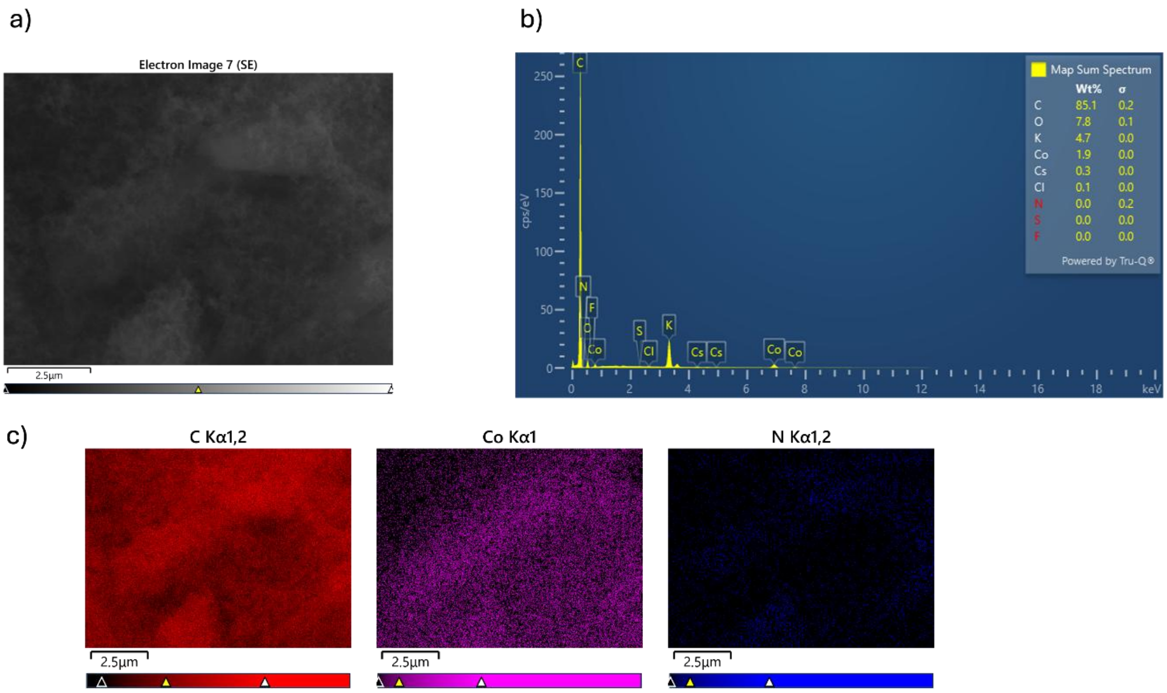

Figure S33. a) SEM image of a **p-CE2** coated GDE after electrolysis performed at r.t.; b) EDX spectrum of the sample; c) elemental mapping of the sample.

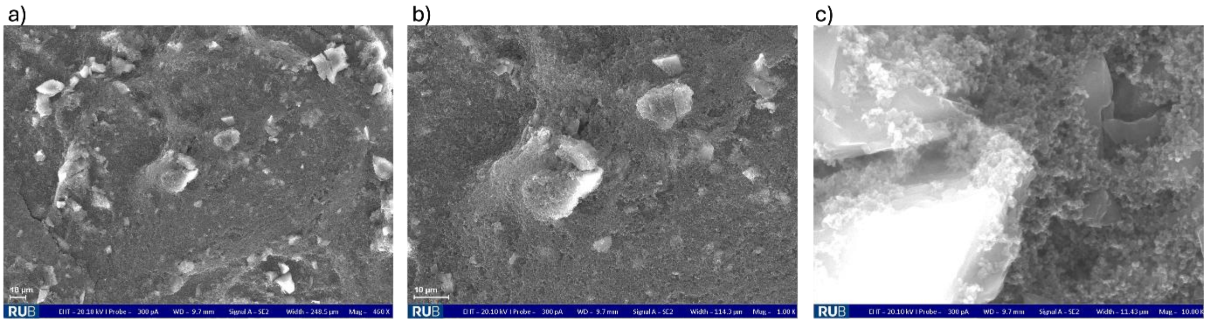

Figure S34. SEM images of a pristine **tBu4** coated GDE at magnifications of a) 460x; b) 1000x and c) 10000x

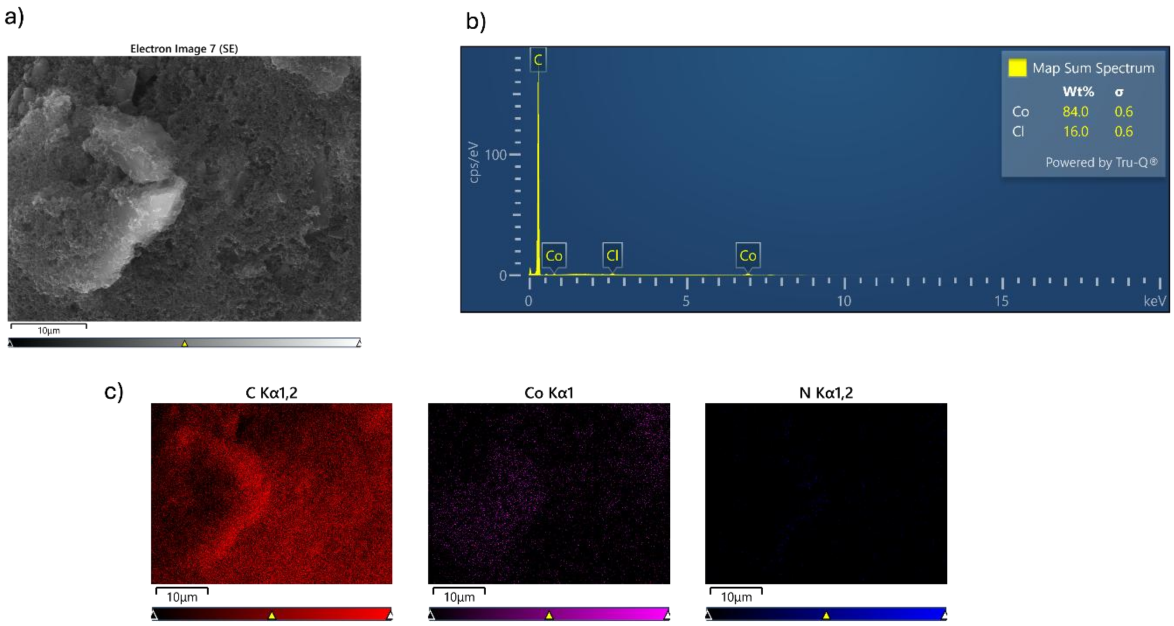

Figure S35. a) SEM image of a pristine **tBu4** coated GDE; b) EDX spectrum of the sample; c) elemental mapping of the sample.

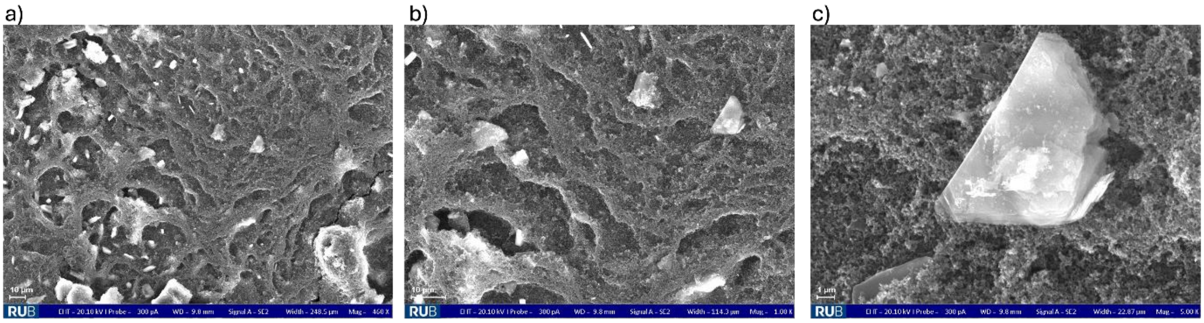

Figure S36. SEM images of a **tBu4** coated GDE after electrolysis performed at r.t. at magnifications of a) 460x; b) 1000x and c) 5000x.

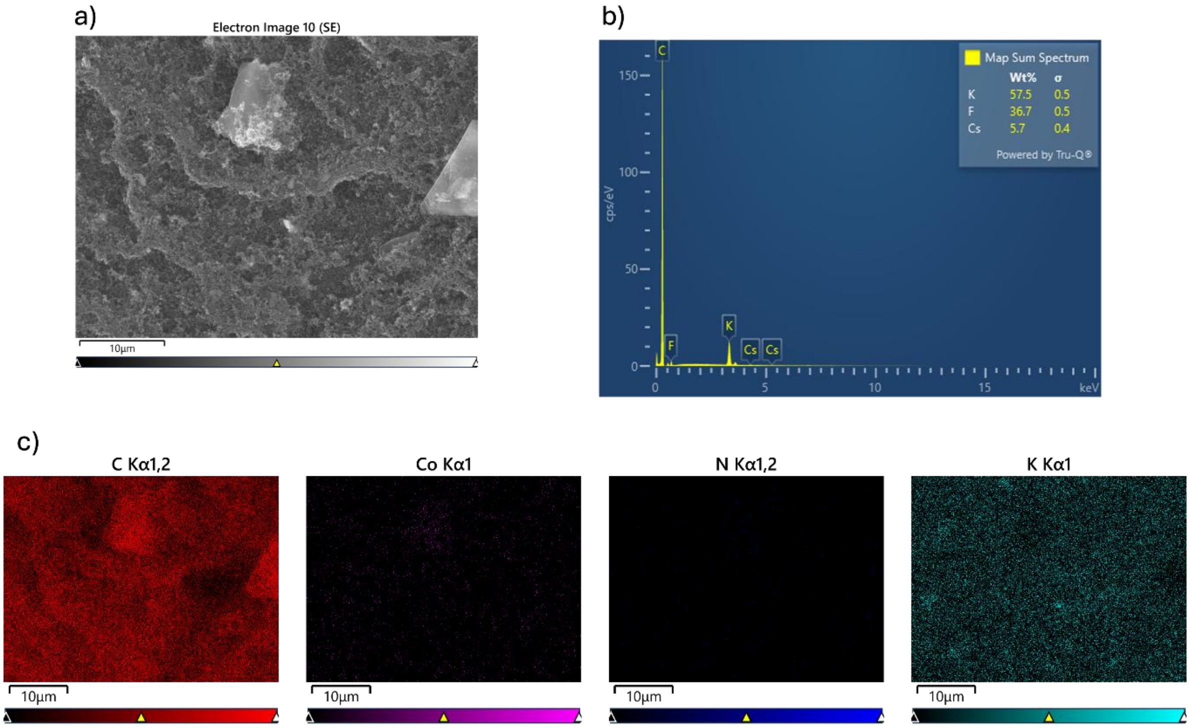

Figure S37. a) SEM image of a pristine **tBu4** coated GDE after electrolysis performed at r.t.; b) EDX spectrum of the sample; c) elemental mapping of the sample.

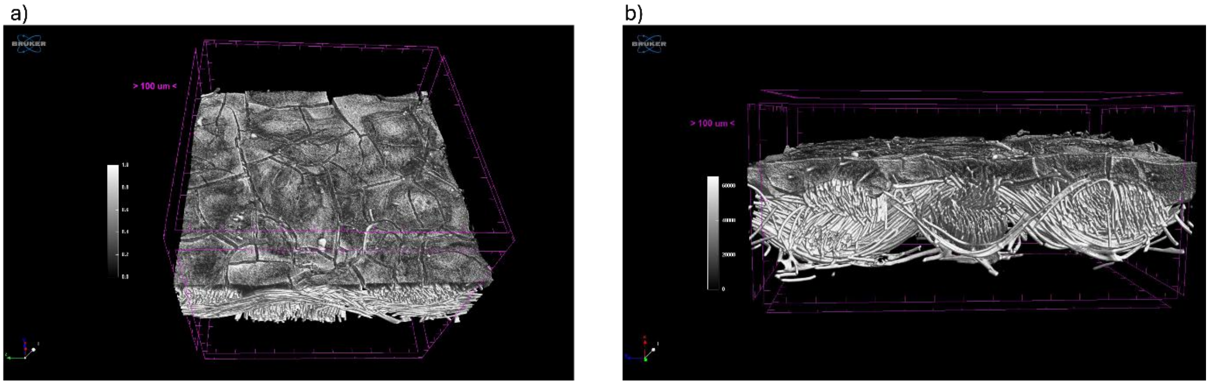

Figure S38. CT images recorded of a pristine **p-CE2** based GDE showing a) the view on the surface and b) a cross-section of the GDE.

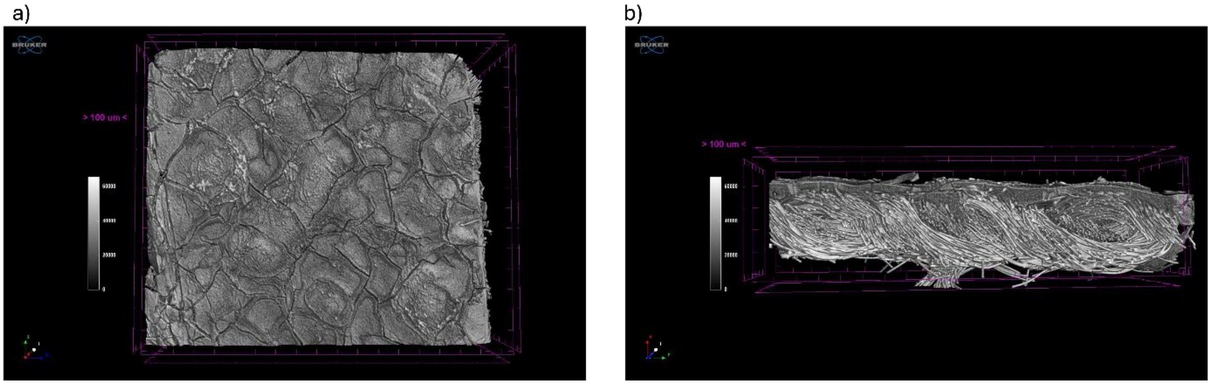

Figure S39. CT images recorded of a **p-CE2** based GDE after electrolysis at r.t. showing a) the view on the surface and b) a cross-section of the GDE.

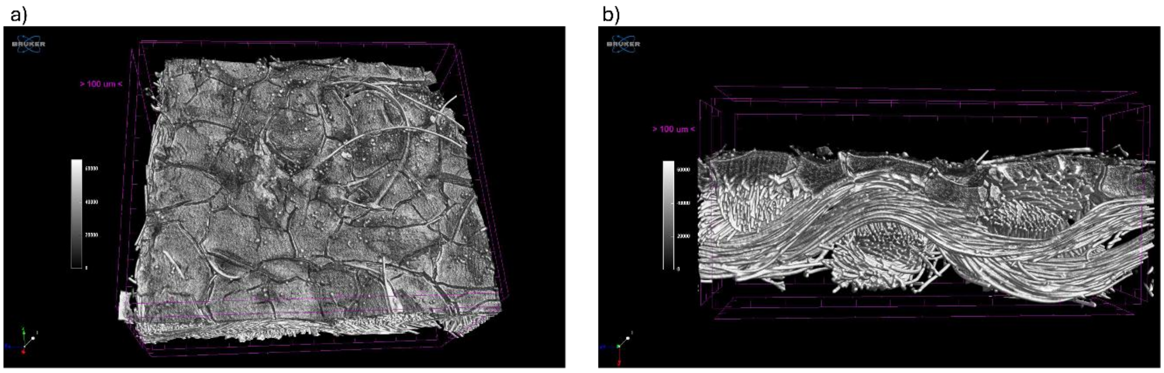

Figure S40. CT images recorded of a pristine **o-CE2** based GDE showing a) the view on the surface and b) a cross-section of the GDE.

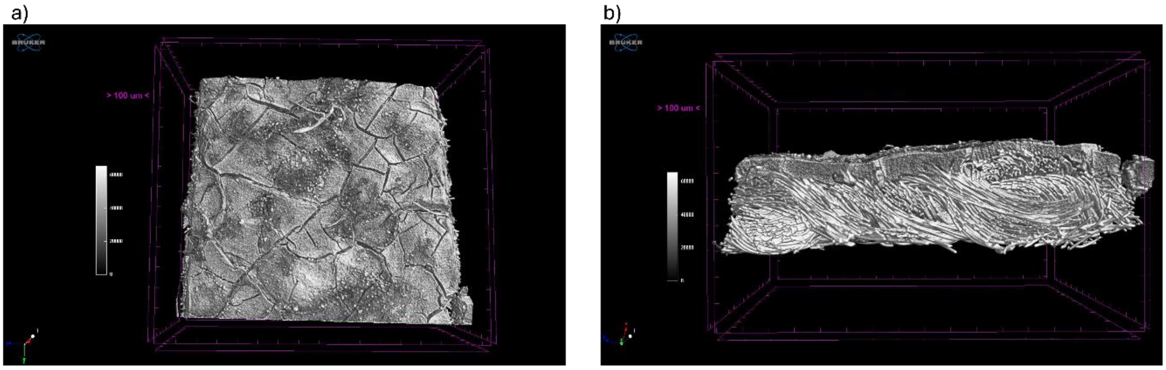

Figure S41. CT images recorded of a **o-CE2** based GDE after electrolysis at r.t. showing a) the view on the surface and b) a cross-section of the GDE.

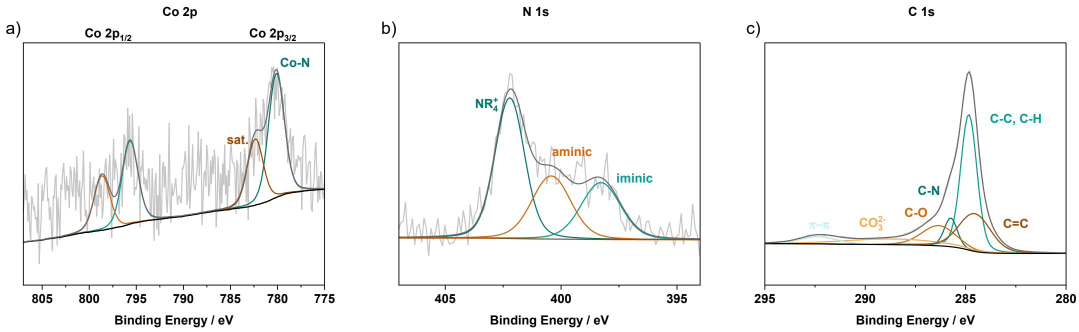

Figure S42. Measured XPS spectra of a pristine GDE coated with **tBu4** a) Co 2p orbital; b) N 1s orbital; c) C 1s orbital.

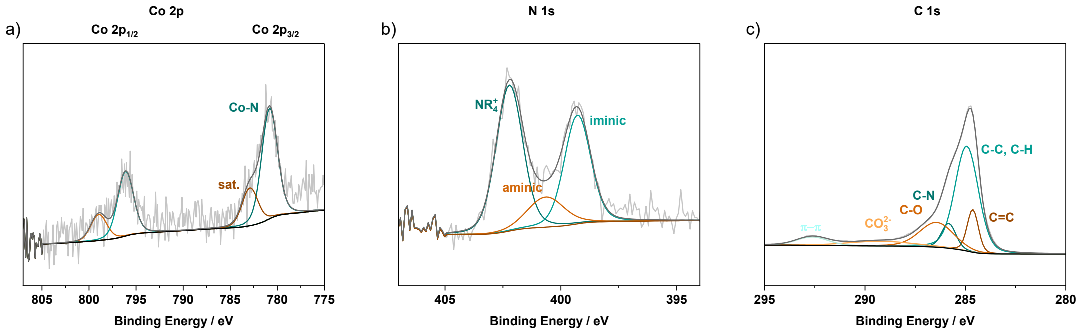

Figure S43. Measured XPS spectra of a pristine GDE coated with **p-CE2** a) Co 2p orbital; b) N 1s orbital; c) C 1s orbital.

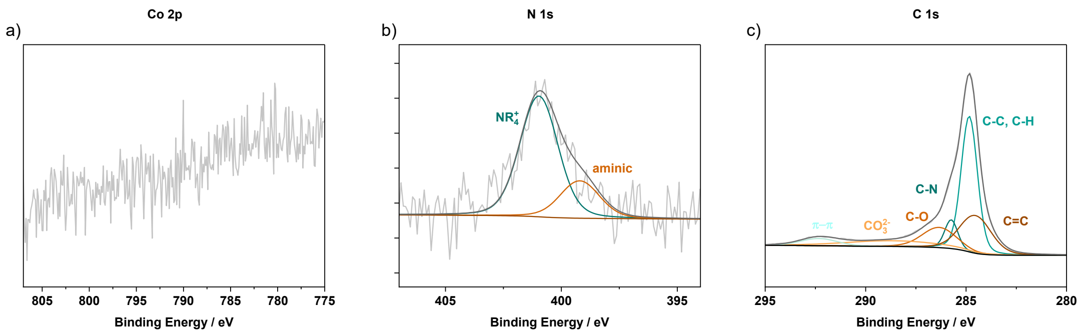

Figure S44. Measured XPS spectra of a GDE coated with **tBu4** after electrolysis at r.t. a) Co 2p orbital; b) N 1s orbital; c) C 1s orbital.

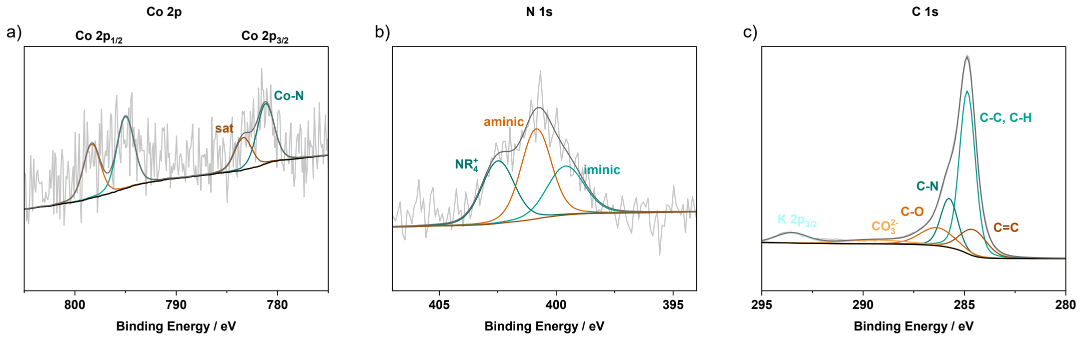

Figure S45. Measured XPS spectra of a GDE coated with **pCE-2** after electrolysis at r.t. a) Co 2p orbital; b) N 1s orbital; c) C 1s orbital.

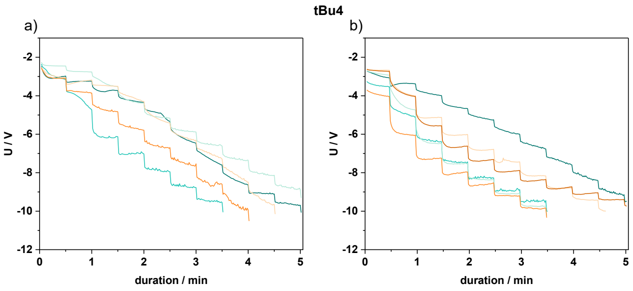

Figure S46. Observed cell voltages when **tBu4** coated GDEs have been tested using 0.1M CsOH as analyte at 60°C with a) no alkali salt addition to the catalytic ink; b) Cs_2CO_3 addition to the catalytic ink which have led to the abortion of electrolysis.

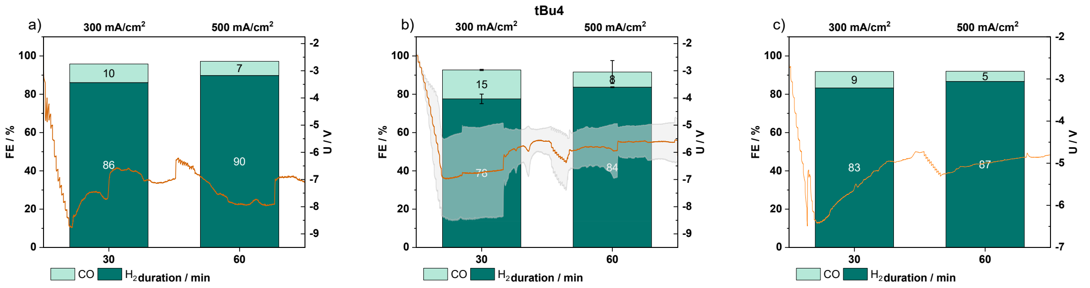

Figure S47. Detailed overview of the achieved FE_{CO} (dark green) and FE_{H2} (light green) pictured as bar chart with the corresponding cell voltage (orange line) achieved with GDEs coated with **tBu4** using 0.1 M CsOH as analyte at 60°C with a) no alkali salt; b) 4 eq. $KHCO_3$ and c) 2 eq. Cs_2CO_3 addition to the catalytic ink which.

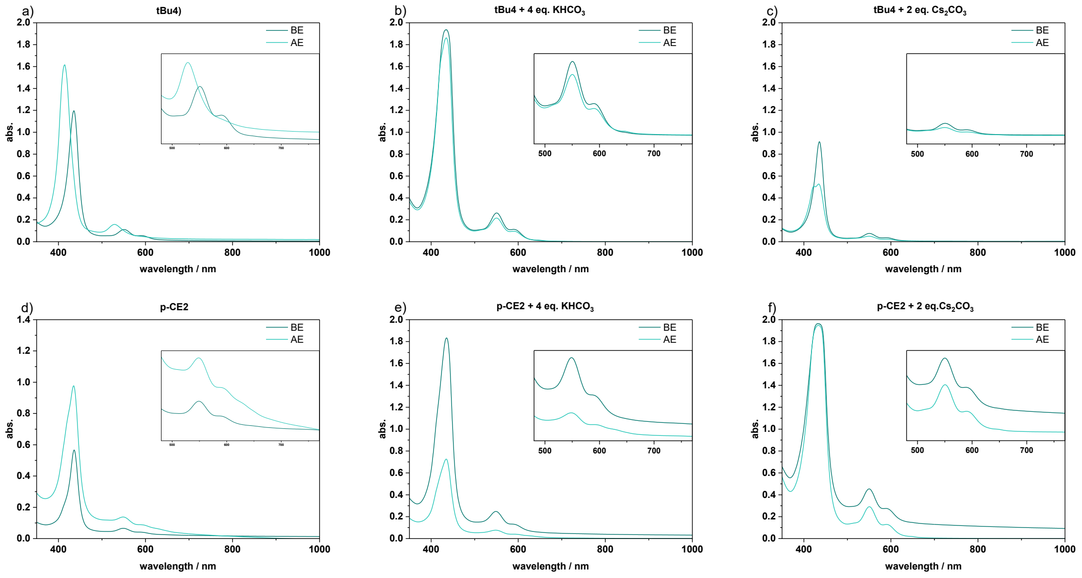

Figure S48. UV/vis spectra in DMSO of redissolved complexes **p-CE2** (A,B) and **tBu4** (C,D) from GDEs in presence of $KHCO_3$ (a,c) or Cs_2CO_3 (b,d).

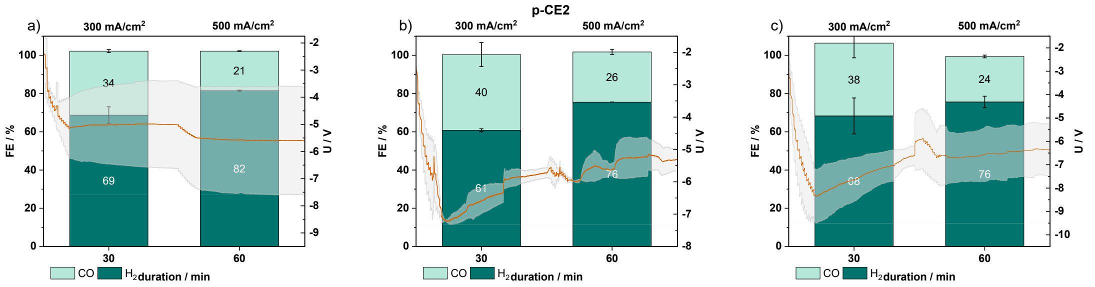

Figure S49. Detailed overview of the achieved FE_{CO} (dark green) and FE_{H2} (light green) pictured as bar chart with the corresponding cell voltage (orange line) achieved with GDEs coated with **p-CE2** using 0.1 M CsOH as anolyte at 60°C with a) no alkali salt; b) 4 eq. $KHCO_3$ and c) 2 eq. Cs_2CO_3 addition to the catalytic ink which..

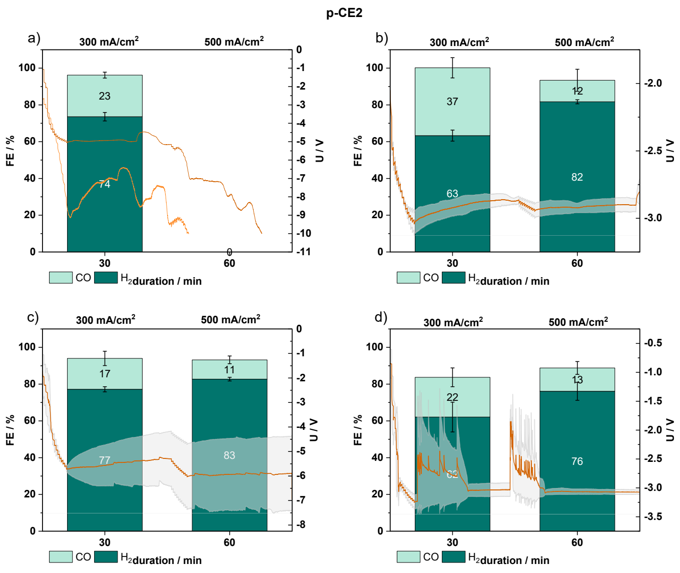

Figure S50. Detailed overview of the achieved FE_{CO} (dark green) and FE_{H2} (light green) pictured as bar chart with the corresponding cell voltage (orange line) achieved with GDEs coated with **p-CE2** at 60 °C using a) 0.1 M KOH; b) 1.0 M KOH; c) 0.1 CsOH and d) 1.0 M CsOH as analyte.

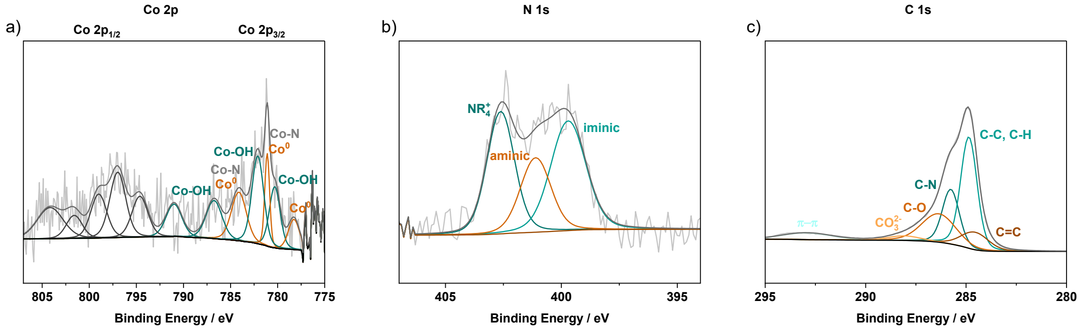

Figure S51. Measured XPS spectra of a GDE coated with **p-CE2** after electrolysis at 60°C using 1 M KOH as analyte a) Co 2p orbital; b) N 1s orbital; c) C 1s orbital.

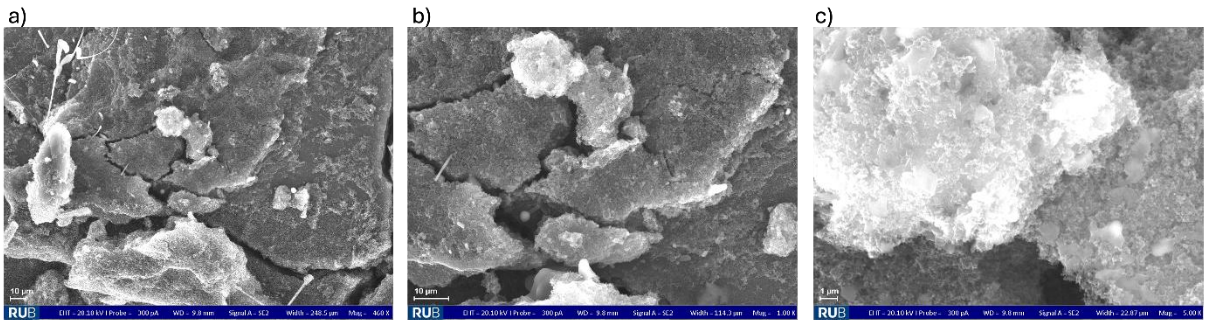

Figure S52. SEM images of a **p-CE2** coated GDE after electrolysis performed at 60 °C using 1 M KOH as analyte at magnifications of a) 460x; b) 1000x and 5000x.

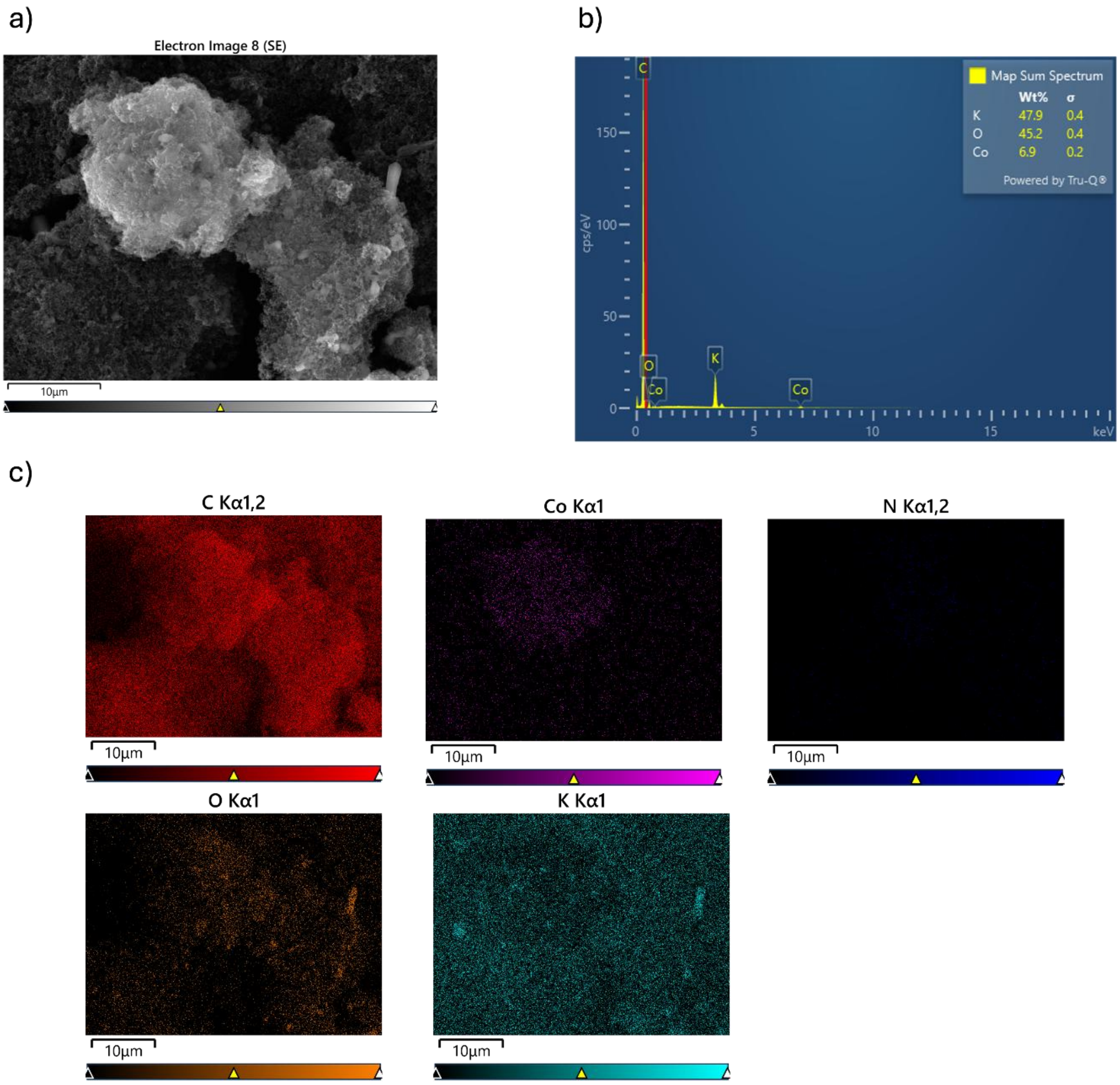

Figure S53. a) SEM image of a **p-CE2** coated GDE after electrolysis performed at 60 °C using 1 M KOH as anolyte; b) EDX spectrum of the sample; c) elemental mapping of the sample.

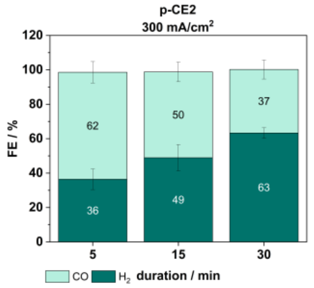

Figure S54. Detailed overview of the achieved FE_{CO} (dark green) and FE_{H2} (light green) achieved with **p-CE2@GDE** after 5, 15 and 30 min of electrolysis at a current density of 300 mA/cm^2 at 60 °C using 1.0 M KOH as anolyte.

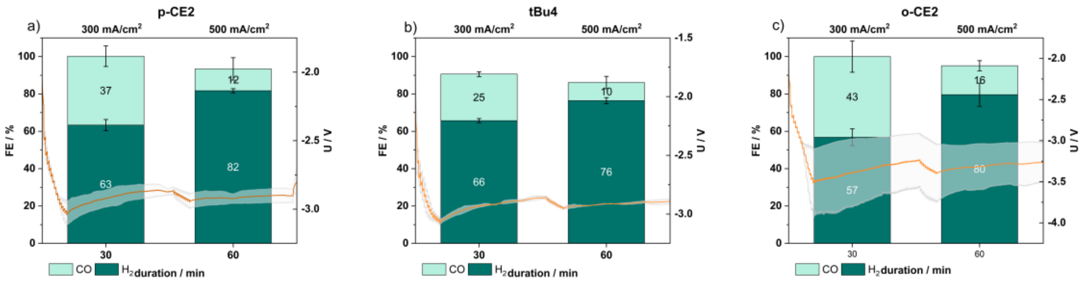

Figure S55. Detailed overview of the achieved FE_{CO} (dark green) and FE_{H2} (light green) pictured as bar chart with the corresponding cell voltage (orange line) achieved with GDEs coated with a) **p-CE2**; b)**tBu4** and c) **o-CE2@GDE** at 60 °C using 1.0 M KOH as anolyte.

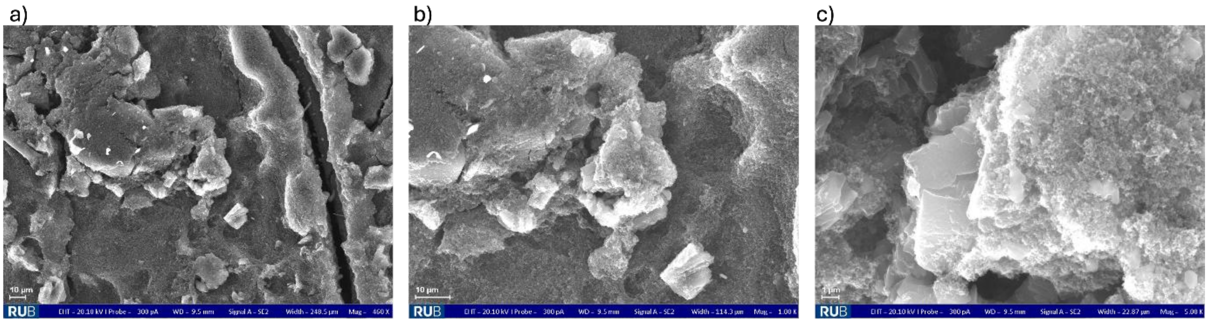

Figure S56. SEM images of a **tBu4** coated GDE after electrolysis performed at 60 °C using 1 M KOH as anolyte at magnifications of a) 460x; b) 1000x and c) 5000x.

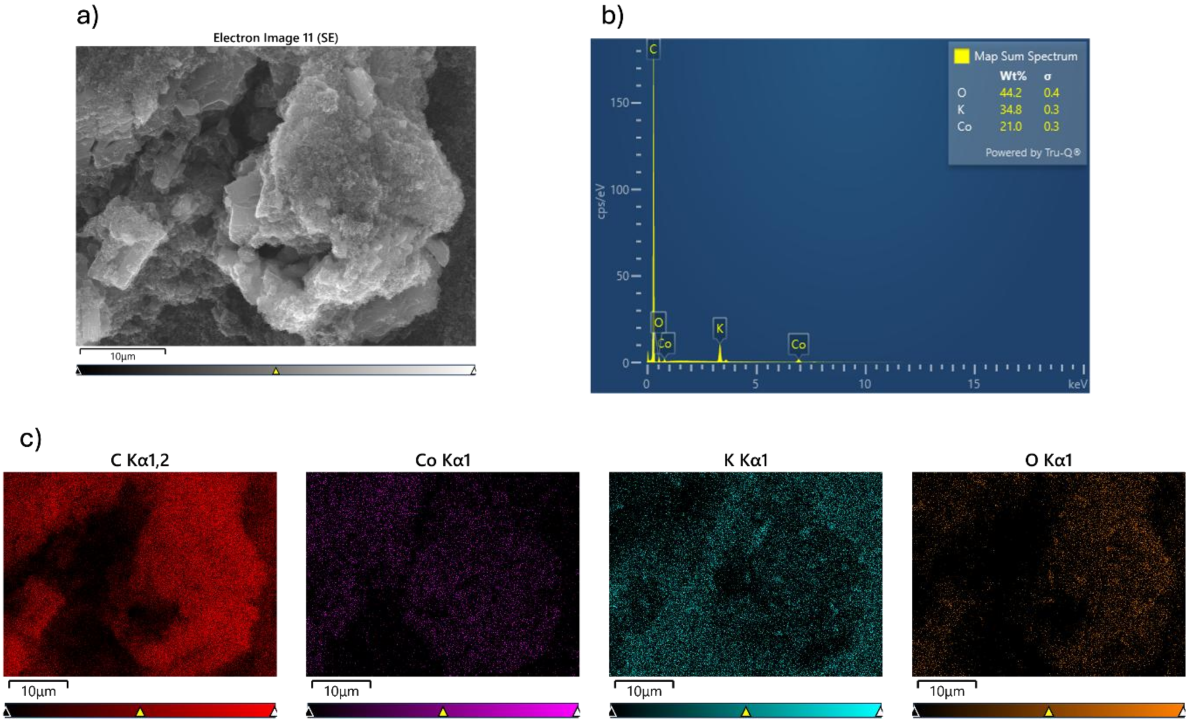

Figure S57. a) SEM image of a pristine **tBu4** coated GDE after electrolysis performed at 60 °C using 1 M KOH as anolyte; b) EDX spectrum of the sample; c) elemental mapping of the sample.

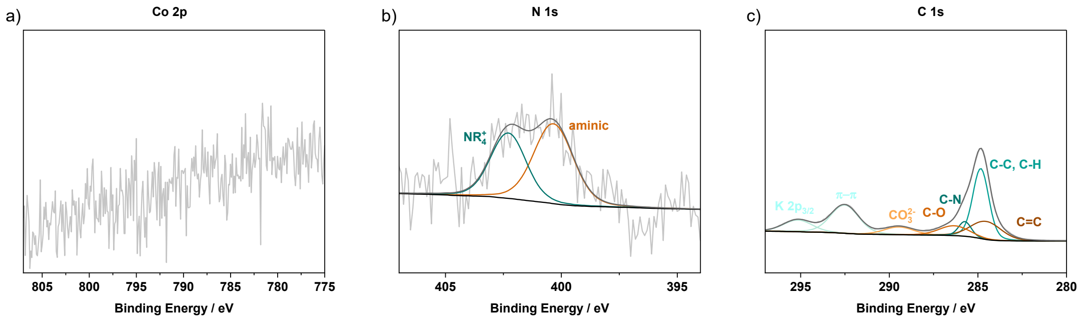

Figure S58. a) Measured XPS spectra of a GDE coated with **tBu4** after electrolysis at 60°C using 1 M KOH as analyte a) Co 2p orbital; b) N 1s orbital; c) C 1s orbital.

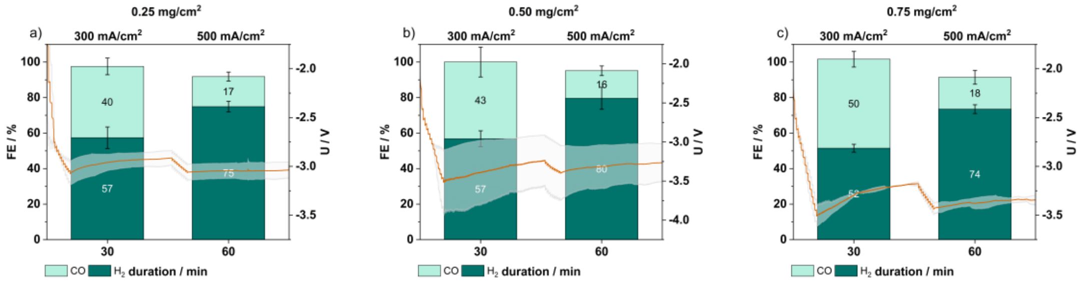

Figure S59. Detailed overview of the achieved FE_{CO} (dark green) and FE_{H2} (light green) and cell voltage (orange line) achieved with **o-CE2@GDE** at loadings of a)0.25 mg/cm^2, b) 0.5 mg/cm^2 and c) 0.75 mg/cm^2 of electrolysis at a current density of 300 mA/cm^2 and 500 mA/cm^2 at 60 °C using 1.0 M KOH as anolyte.

NMR-spectra

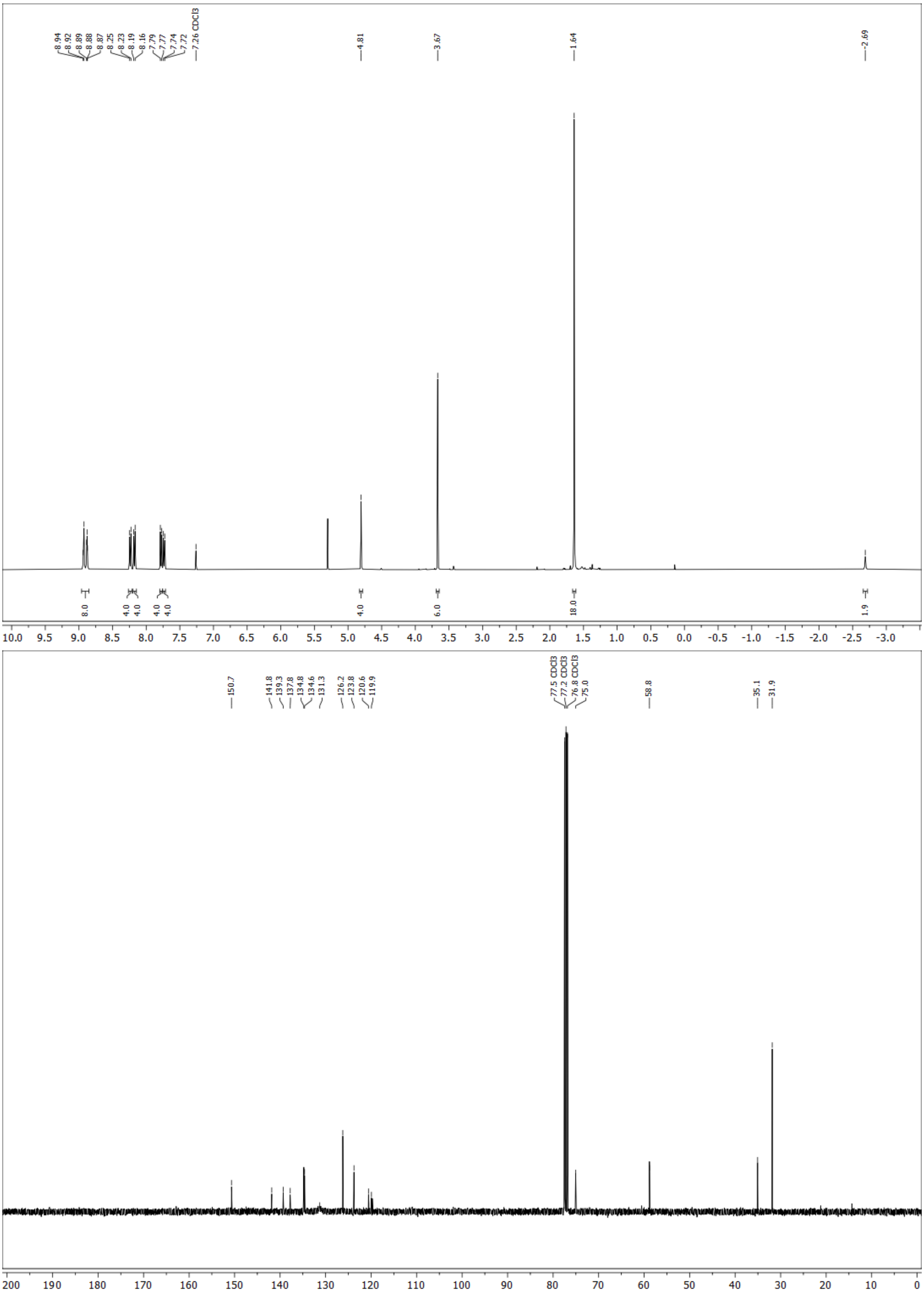

Figure S60. ^{1}H-NMR (top) and ^{13}C-NMR (bottom) of **2.**

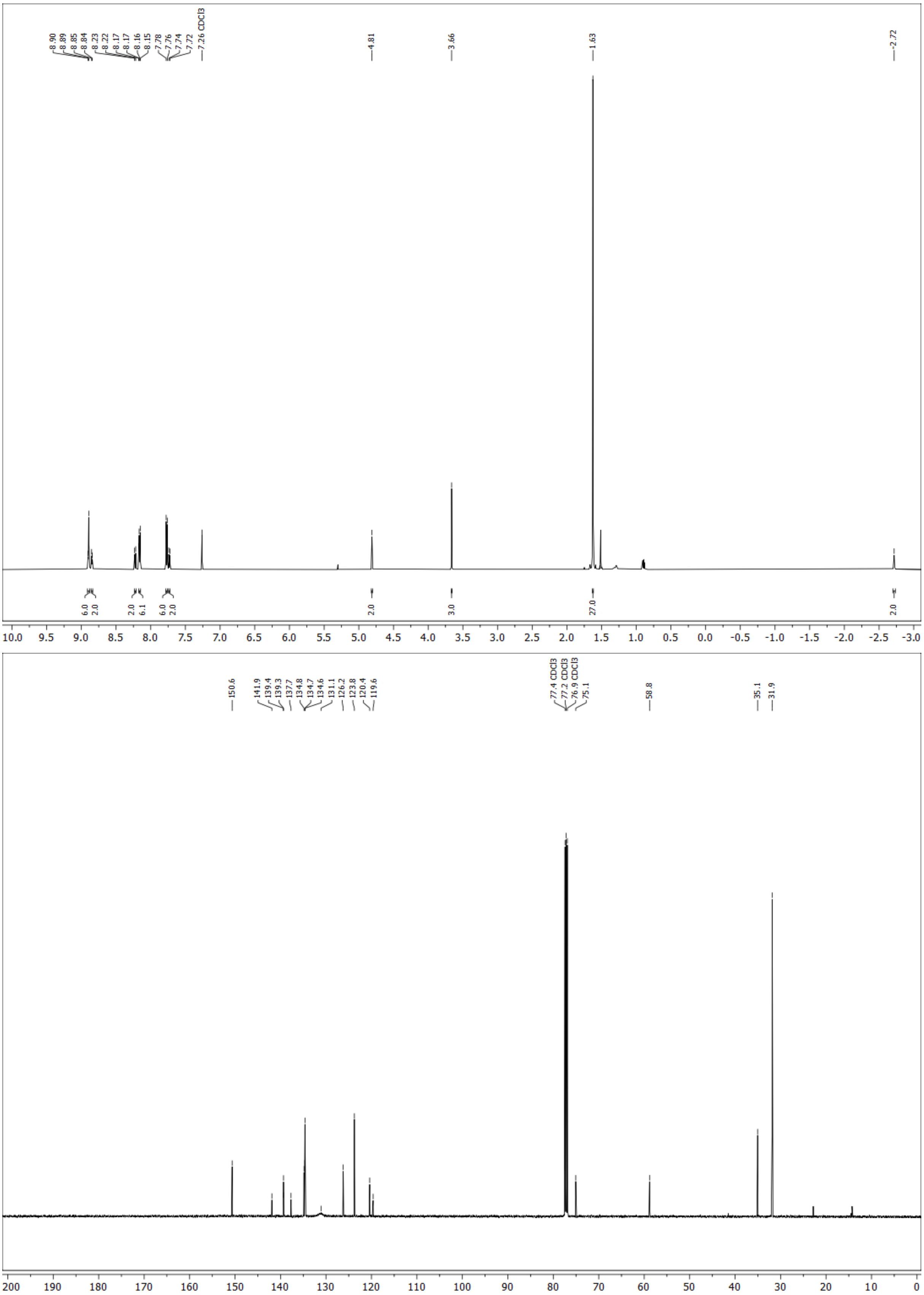

Figure S61. ^{1}H-NMR (top) and ^{13}C-NMR (bottom) of **3**.

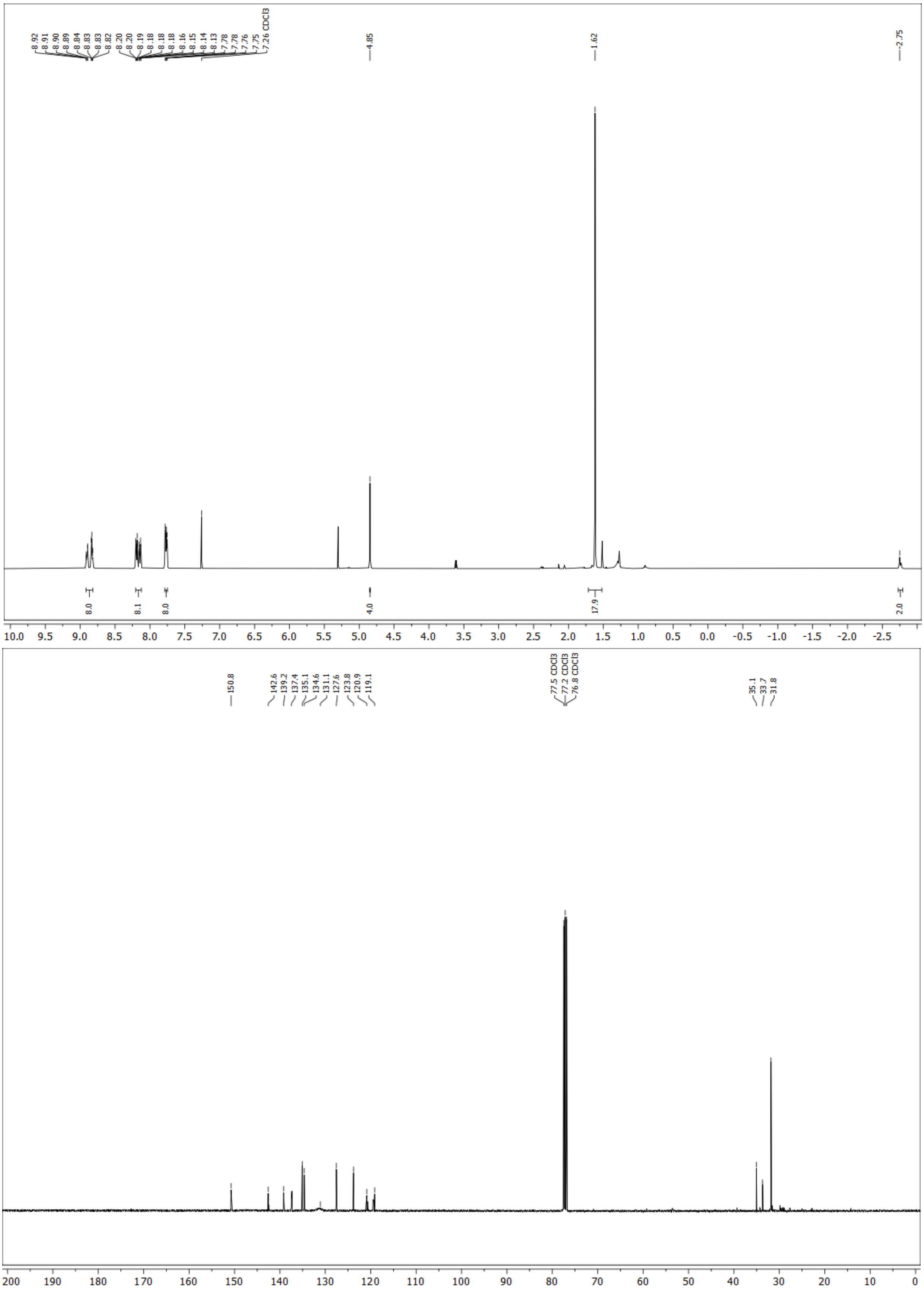

Figure S62. ^{1}H-NMR (top) and ^{13}C-NMR (bottom) of **4**.

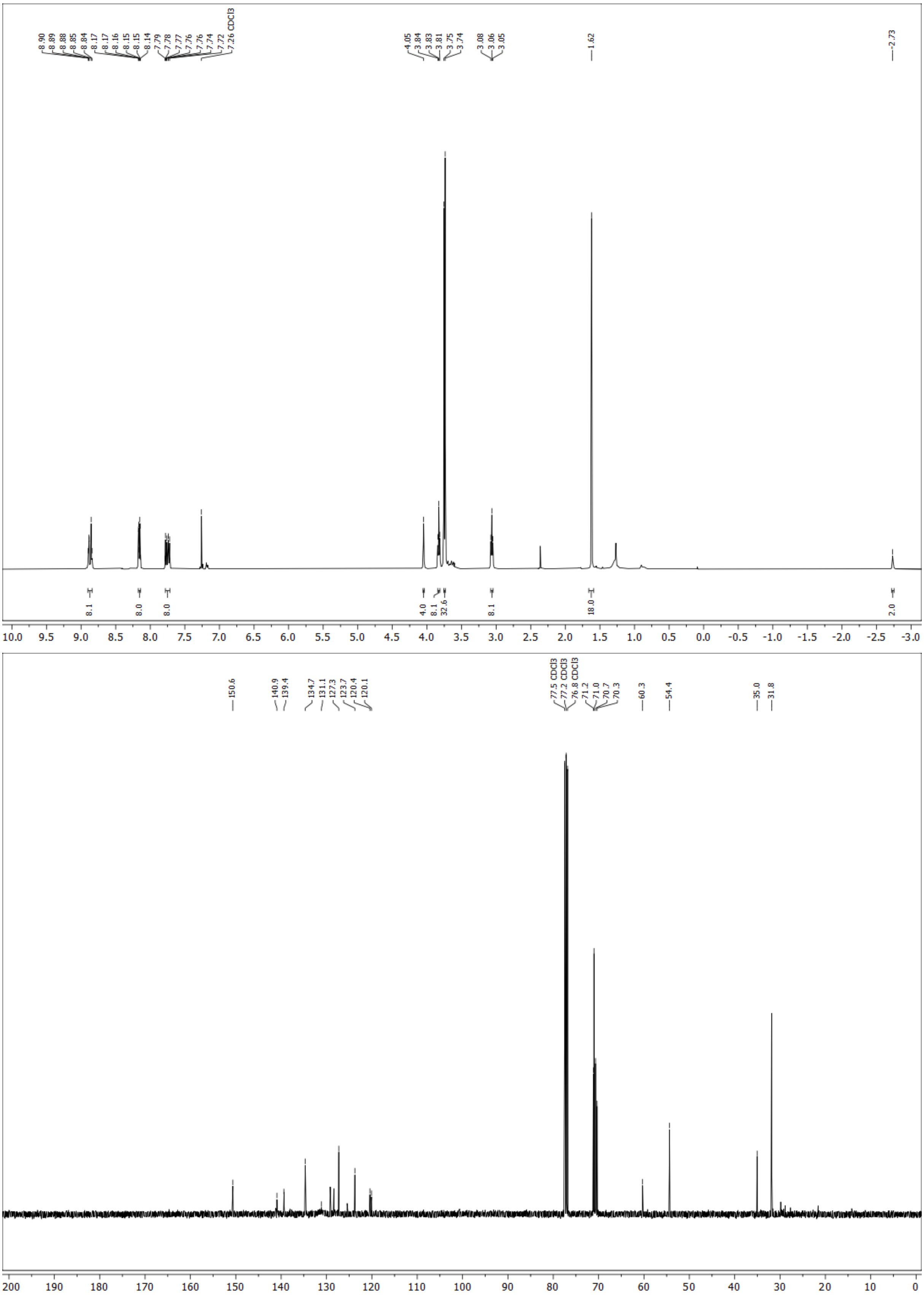

Figure S63. ^{1}H-NMR (top) and ^{13}C-NMR (bottom) of **5**.

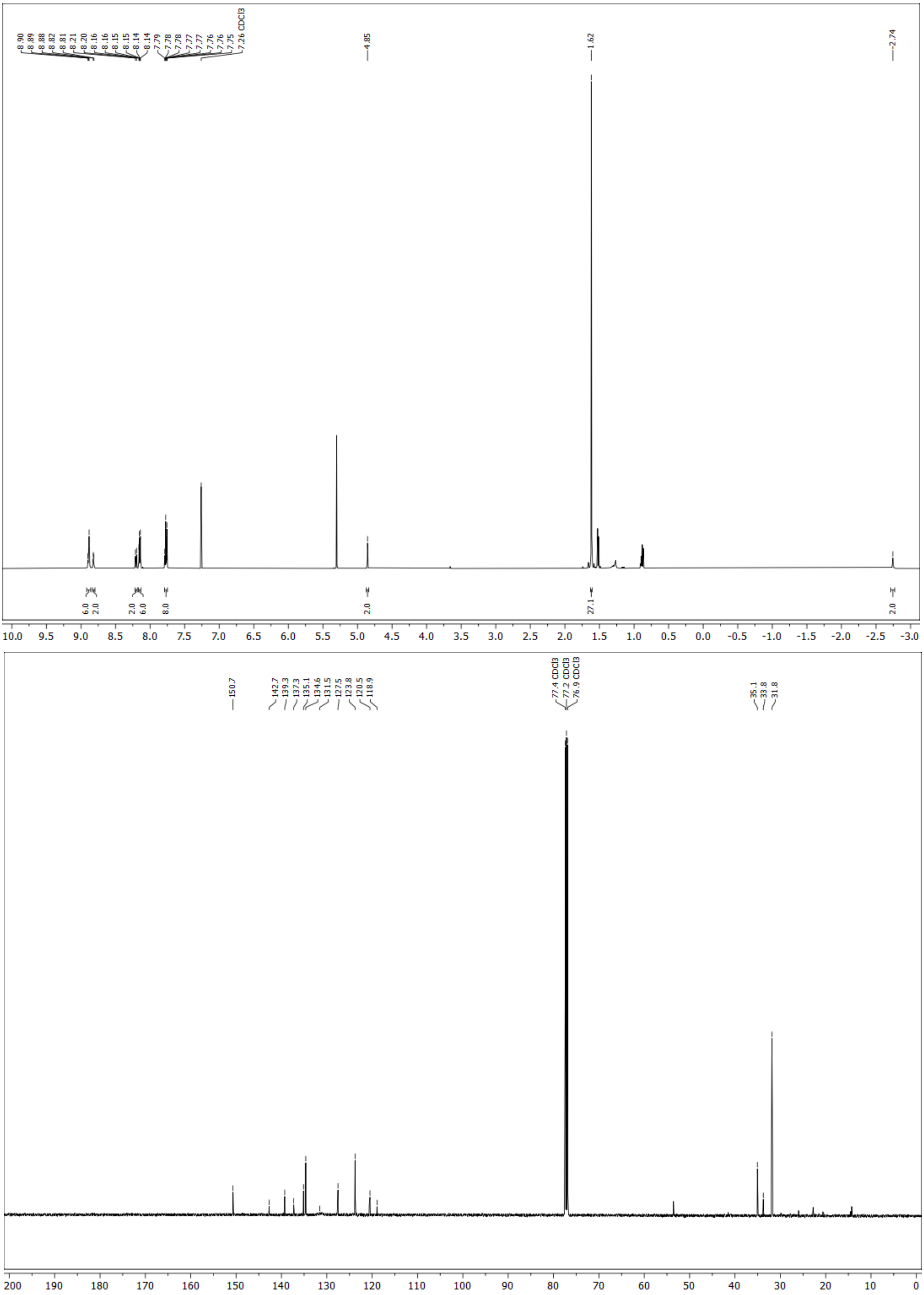

Figure S64. ^{1}H-NMR (top) and ^{13}C-NMR (bottom) of **6**.

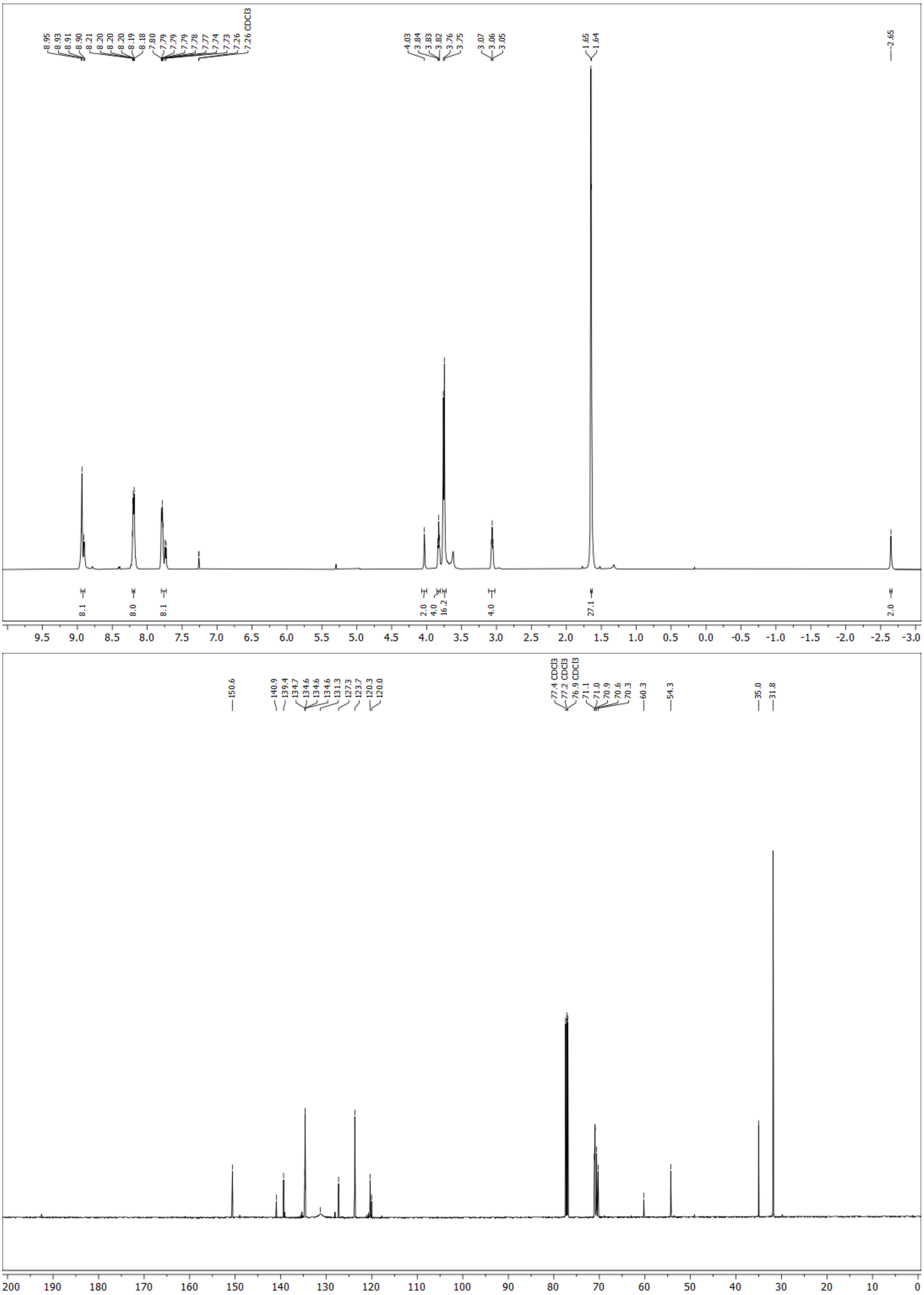

Figure S65. ^{1}H-NMR (top) and ^{13}C-NMR (bottom) of **7.**

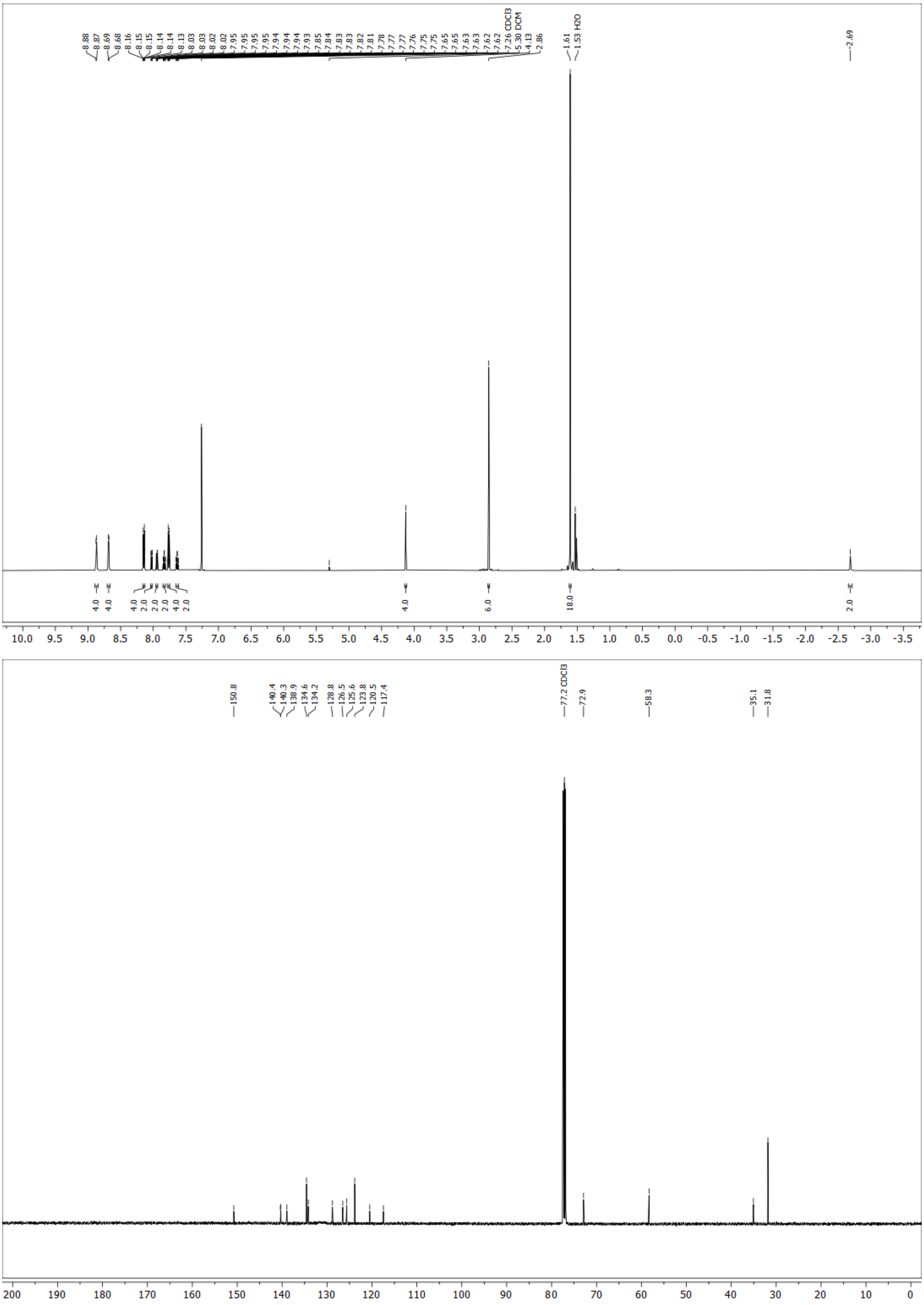

Figure S66. ^{1}H-NMR (top) and ^{13}C-NMR (bottom) of **8**.

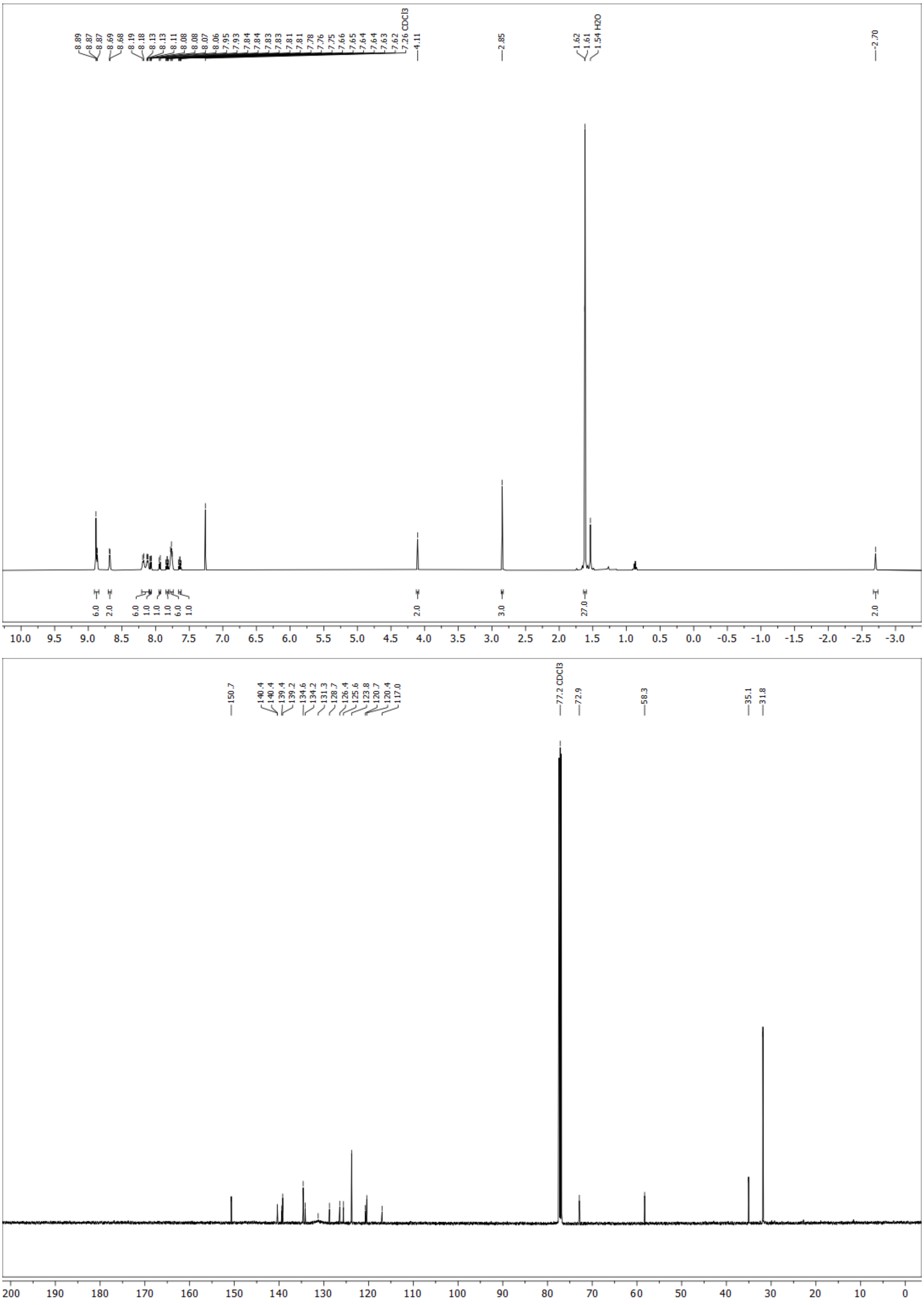

Figure S67. ^{1}H-NMR (top) and ^{13}C-NMR (bottom) of **9**.

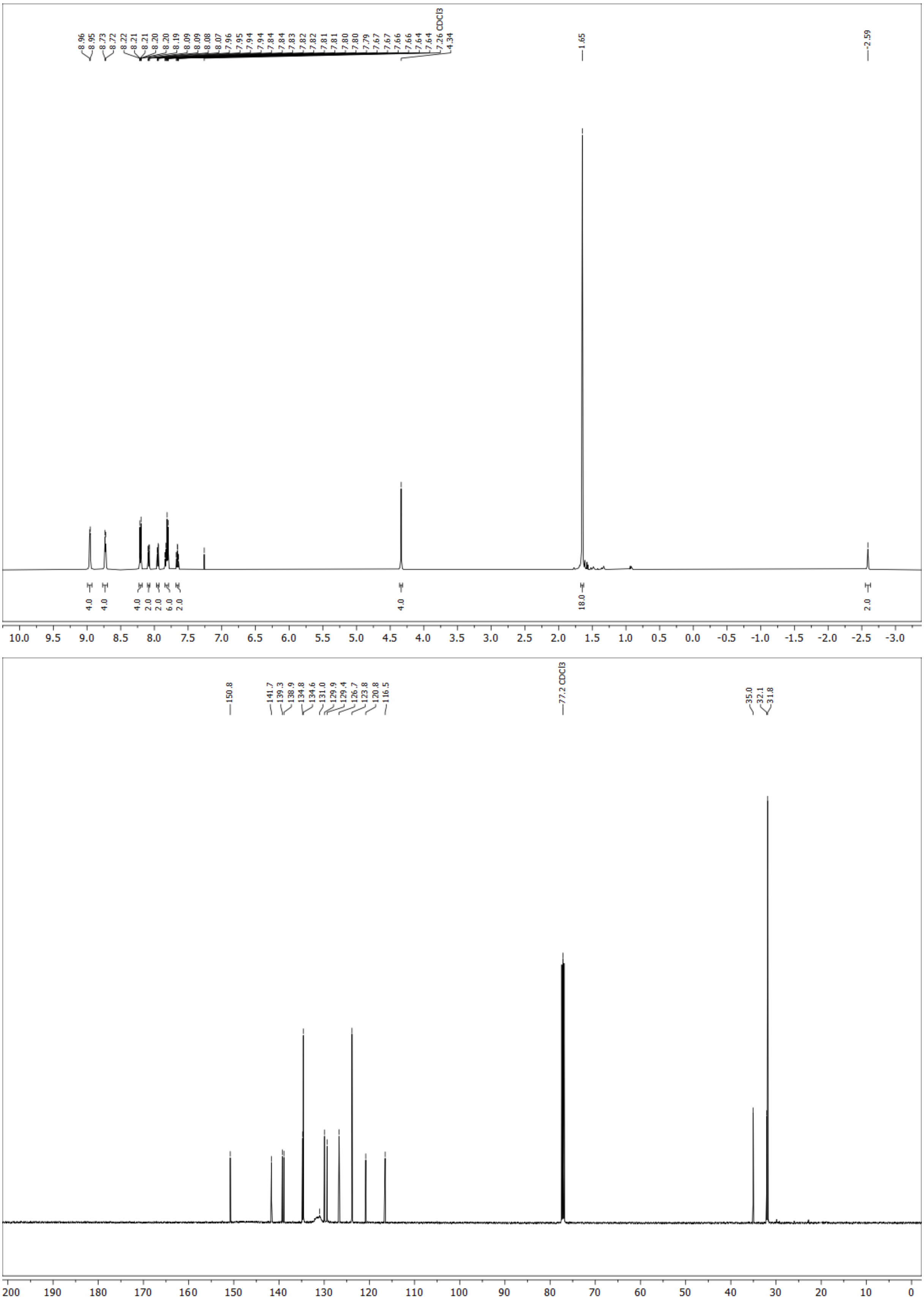

Figure S68. ^{1}H-NMR (top) and ^{13}C-NMR (bottom) of **10**.

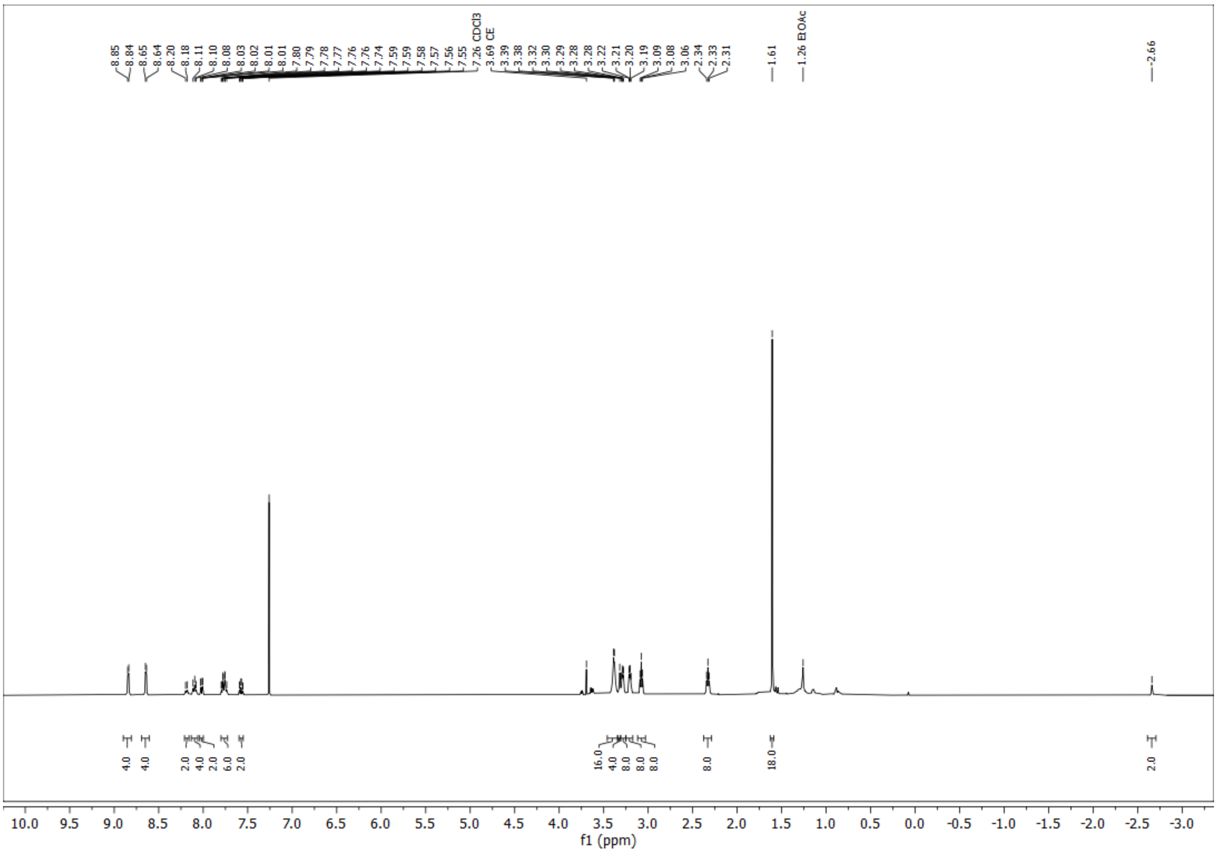

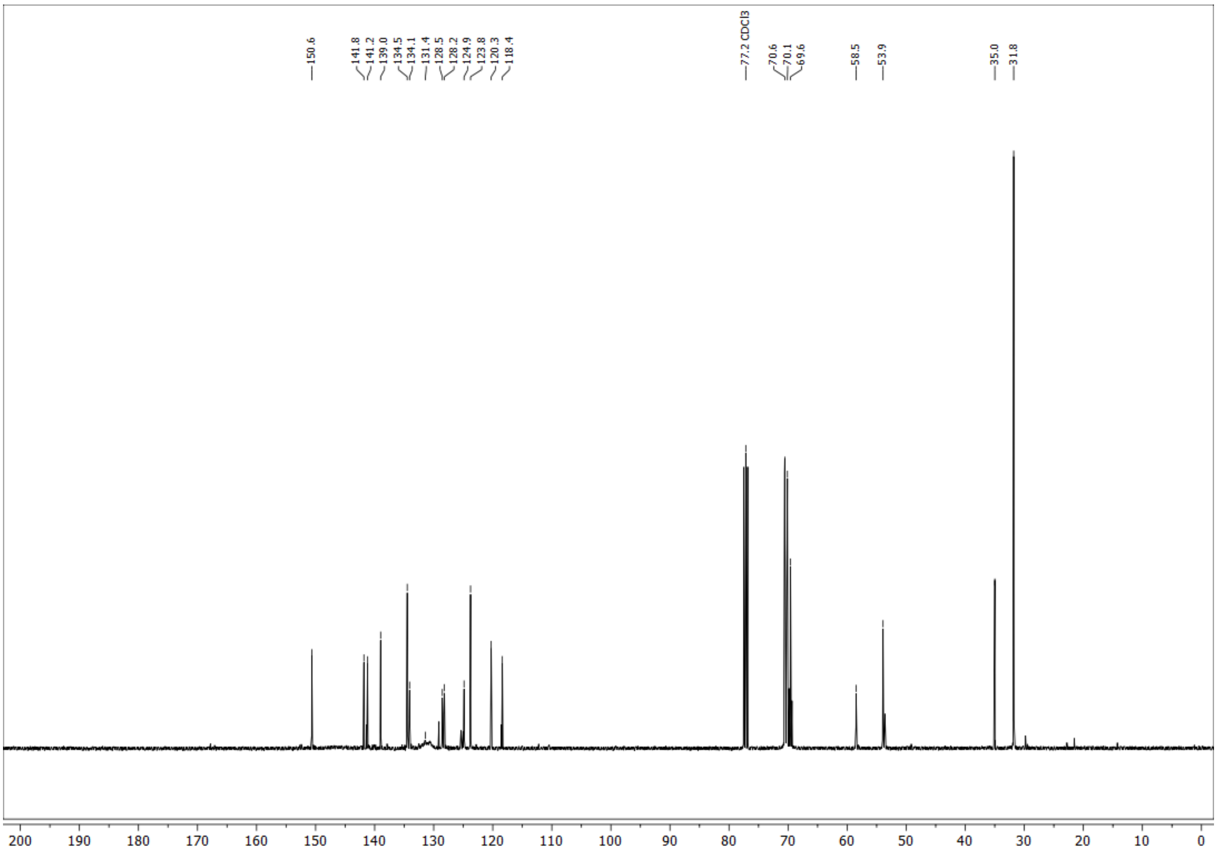

Figure S69. ^{1}H-NMR (top) and ^{13}C-NMR (bottom) of **11**.

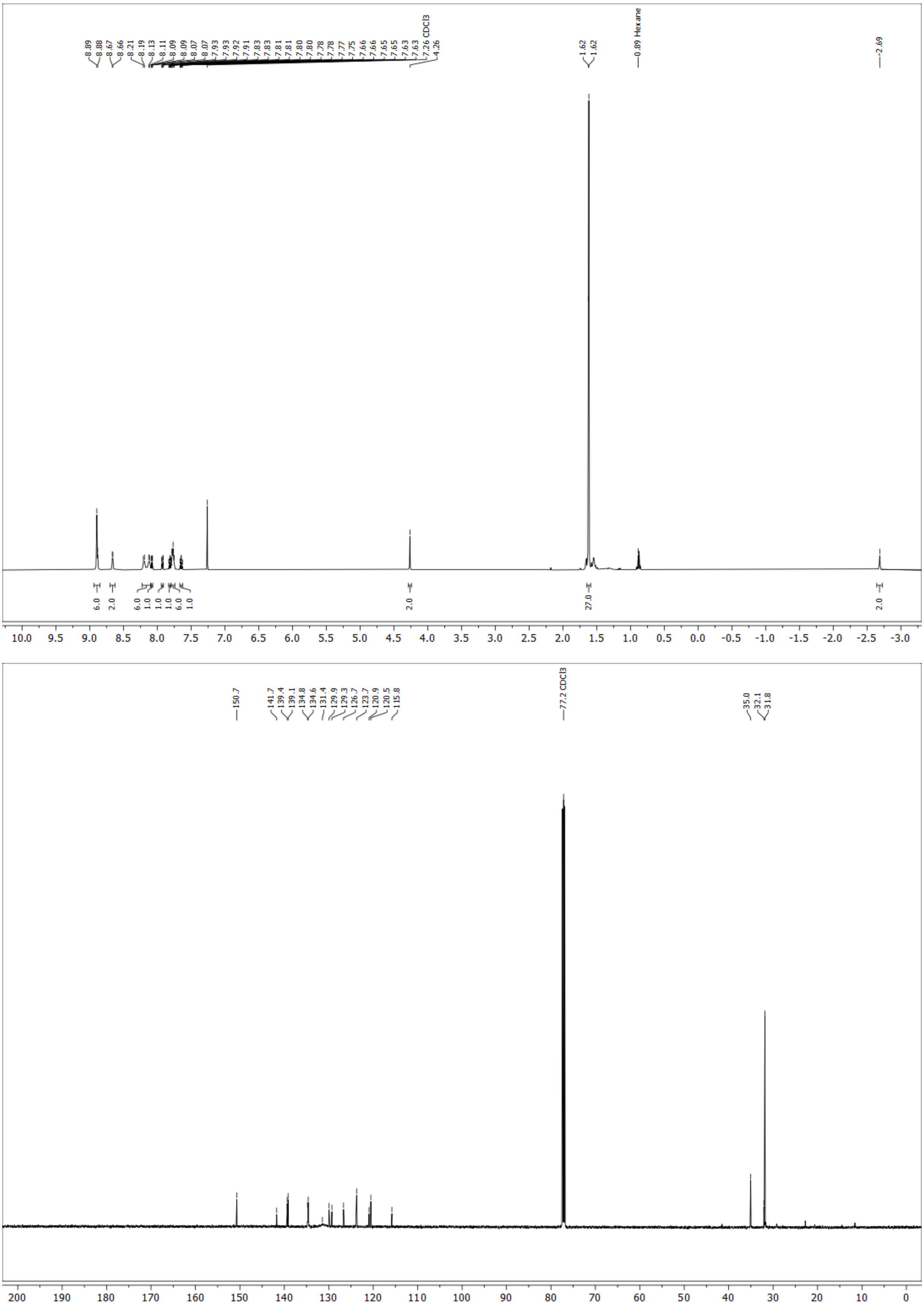

Figure S70. ^{1}H-NMR (top) and ^{13}C-NMR (bottom) of **12**.

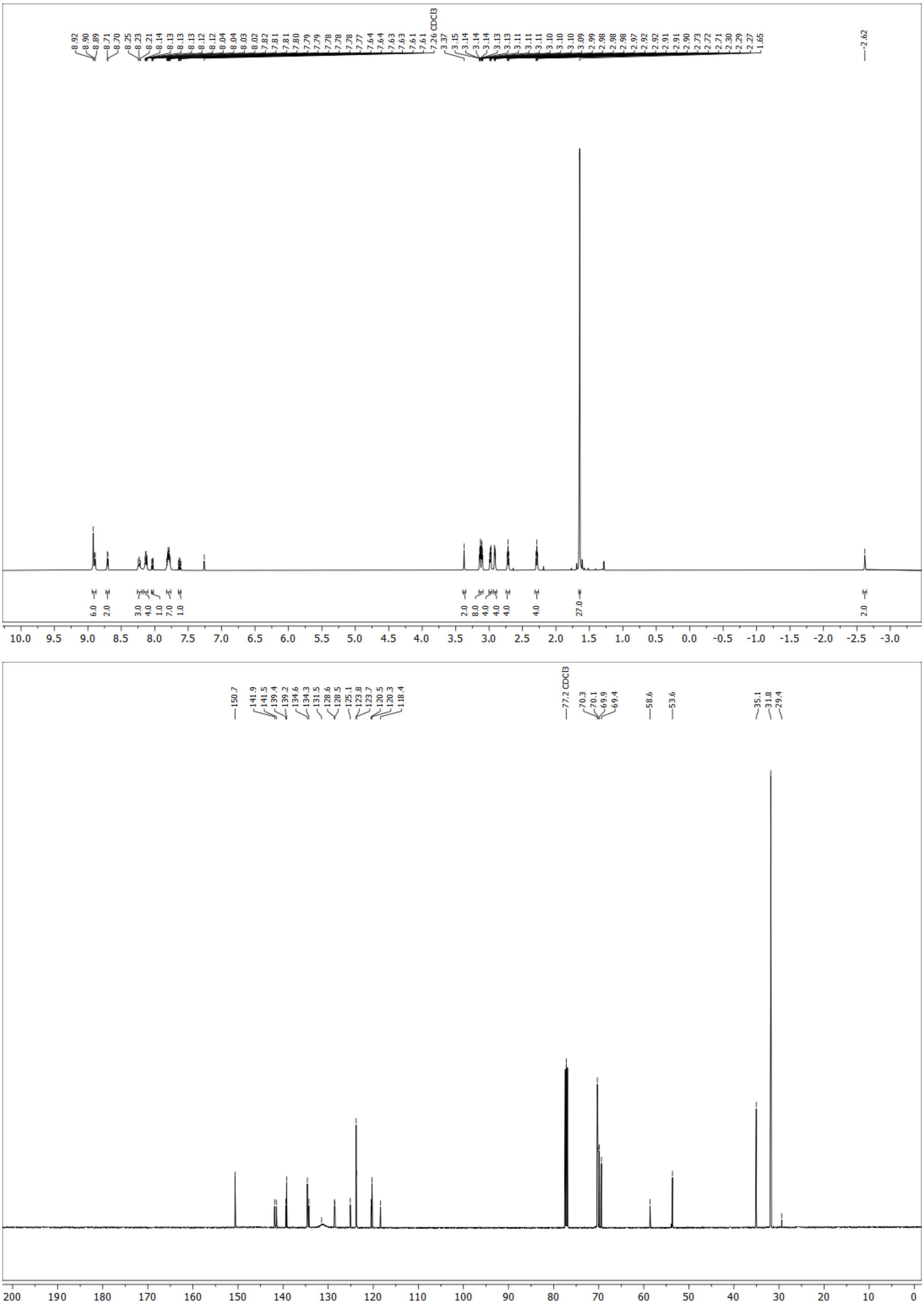

Figure S71. ^{1}H-NMR (top) and ^{13}C-NMR (bottom) of **13**.

ESI-MS-spectra

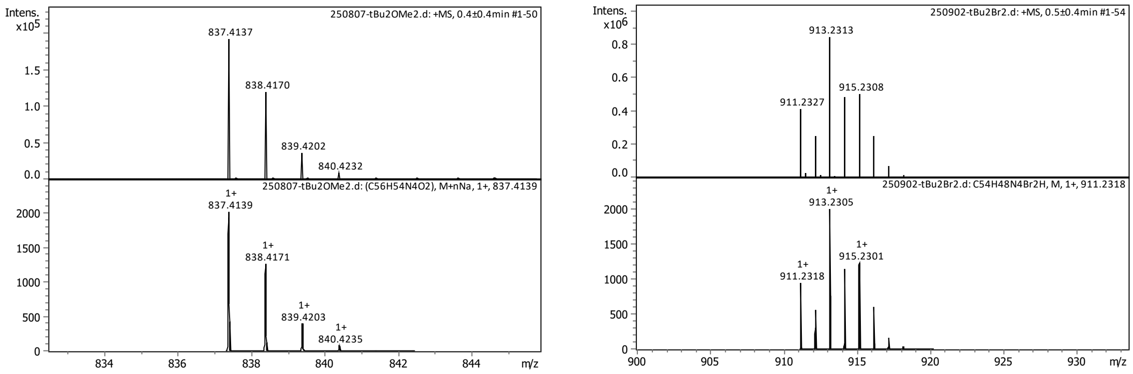

Figure S72. ESI-MS spectra of **2** (left) and **4** (right).

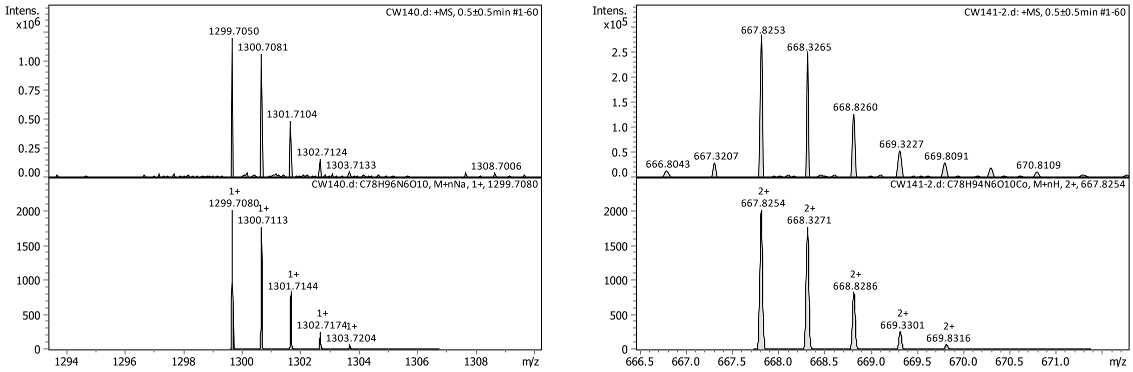

Figure S73. ESI-MS spectra of **5** (left) and **p-CE2** (right).

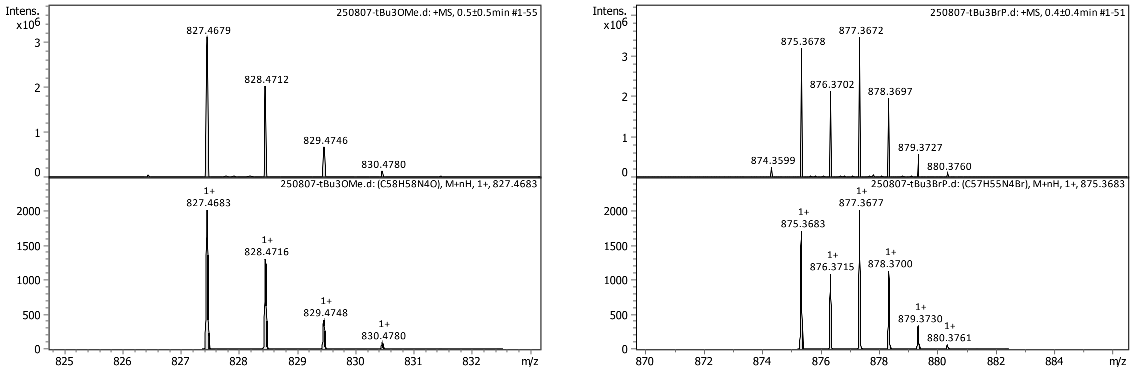

Figure S74. ESI-MS spectra of **3** (left) and **6** (right).

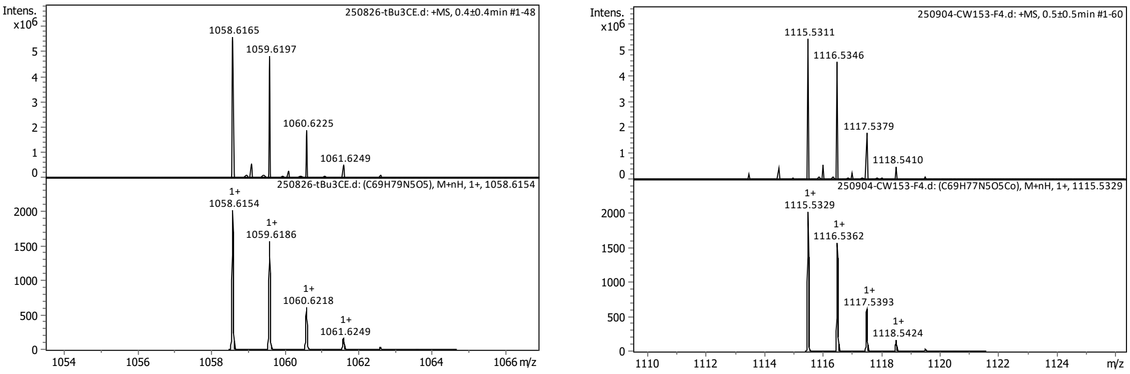

Figure S75. ESI-MS spectra of **7** (left) and **p-CE** (right).

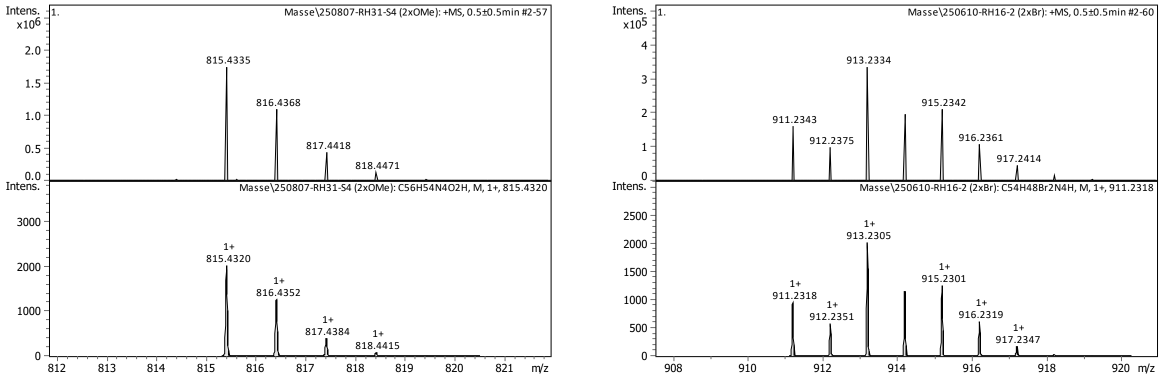

Figure S76. ESI-MS spectra of **8** (left) and **10** (right).

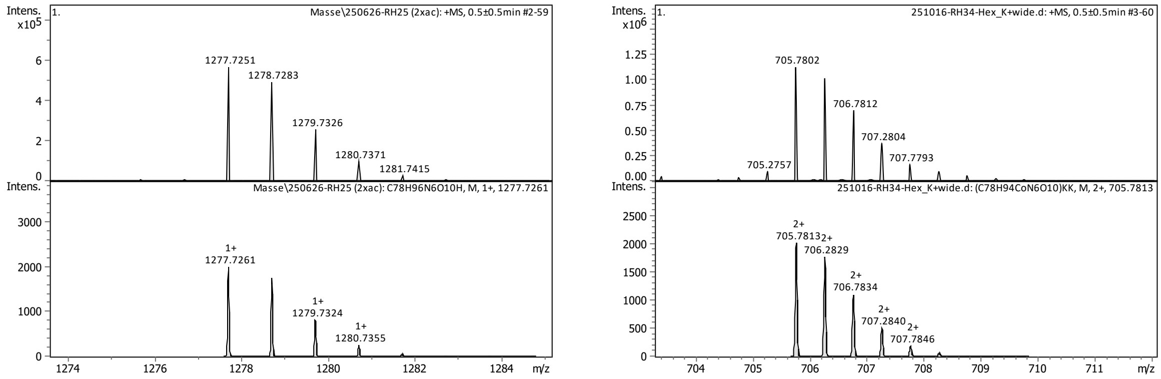

Figure S77. ESI-MS spectra of **11** (left) and **o-CE2** (right).

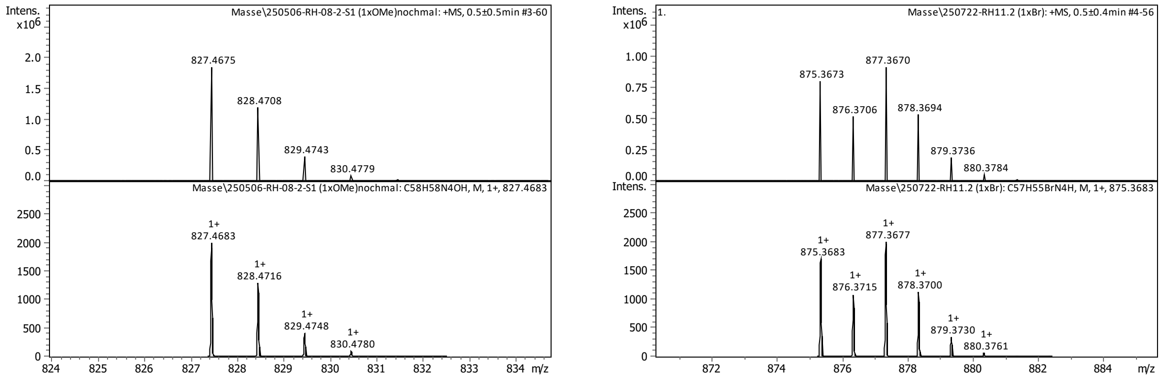

Figure S78. ESI-MS spectra of **9** (left) and **12** (right).

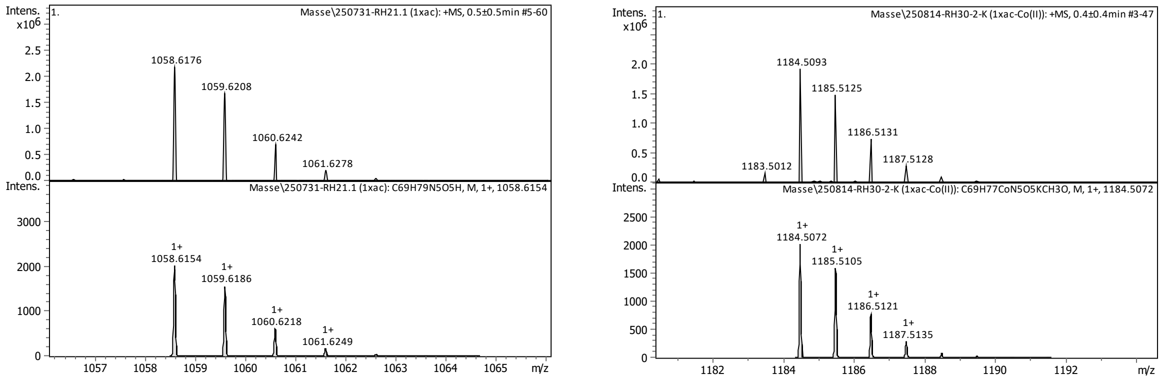

Figure S79. ESI-MS spectra of **13** (left) and **o-CE** (right).

References

[1] Z. Chen, B. Wang, Z. Wang, G. Zhu, J. Sun, “Complex Bioactive Alkaloid-Type Polycycles through Efficient Catalytic Asymmetric Multicomponent Aza-Diels–Alder Reaction of Indoles with Oxetane as Directing Group” *Angew Chem Int Ed* **2013**, *52*, 2027–2031.

[2] E. Jaworska, M. L. Naitana, E. Stelmach, G. Pomarico, M. Wojciechowski, E. Bulska, K. Maksymiuk, R. Paolesse, A. Michalska, “Introducing Cobalt(II) Porphyrin/Cobalt(III) Corrole Containing Transducers for Improved Potential Reproducibility and Performance of All-Solid-State Ion-Selective Electrodes” *Anal. Chem.* **2017**, *89*, 7107–7114.

[3] D. Y. Shin, J. H. Yoon, S. H. Kim, H. Baik, S. J. Lee, “Immobilization of a porphyrinic Mn(III) catalyst on a new type of silica support comprising a three-dimensionally interconnected network with two different sizes of pores” *Catal. Sci. Technol.* **2018**, *8*, 6306–6310.

[4] C. Zhang, H. Long, W. Zhang, “A C84 selective porphyrin macrocycle with an adaptable cavity constructed through alkyne metathesis” *Chem. Commun.* **2012**, *48*, 6172.

[5] M. Wag, K. Torbensen, D. Salvatore, S. Ren, D. Joulié, F. Dumoulin, D. Mendoza, B. Lassalle-Kaiser, U. Ișci, C.P. Berlinguette, M. Robert “CO_2 electrochemical catalytic reduction with a highly active cobalt phthalocyanine” *Nat. Commun.* **2019**, *10*, 3602.

[6] L. Hoof, N. Thissen, K. Pellumbi, K. Junge Puring, D. Siegmund, A. K. Mechler, U.-P. Apfel, “Hidden parameters for electrochemical carbon dioxide reduction in zero-gap electrolyzers” *Cell Reports Physical Science* **2022**, *3*, 100825.
